# Efficacy and safety of chimeric antigen receptor T-cell in the treatment of hematologic malignancy: an umbrella review of systematic review and meta-analysis

**DOI:** 10.3389/fimmu.2025.1608768

**Published:** 2025-11-19

**Authors:** Zhengyu Yu, Caixia Jing, Li Xie, Lang Min, Lingfeng Li, Zhongwang Wang, Ting Niu

**Affiliations:** 1 Department of Hematology, West China Hospital, Sichuan University, Chengdu, China; 2 State Key Laboratory of Wildlife Quarantine and Surveillance (Sichuan), Technology Center of Chengdu Customs, Chengdu, China

**Keywords:** CAR-T therapy, acute lymphoblastic leukemia, diffuse large B-cell lymphoma, meta-analyses, umbrella review

## Abstract

**Background:**

This umbrella review consolidates data from systematic reviews and meta-analyses on the efficacy and safety of Chimeric Antigen Receptor T-cell (CAR-T) therapy in hematologic malignancies. The aim is to assess CAR-T efficacy across different malignancies, identify key safety concerns, and provide clinical recommendations.

**Methods:**

We conducted a thorough search of PubMed, Embase, Web of Science, and the Cochrane Database of Systematic Reviews up to May 2024. Systematic reviews and meta-analyses evaluating CAR-T efficacy in hematologic malignancies were included. The AMSTAR tool was used to assess methodological quality, and the GRADE system was employed to evaluate the quality of evidence for each outcome.

**Results:**

A total of 105 meta-analyses met the inclusion criteria. CD19-targeted CAR-T therapies demonstrated superior efficacy in acute lymphoblastic leukemia (ALL) and diffuse large B-cell lymphoma (DLBCL), particularly in relapsed or refractory cases (high-quality). However, CAR-T monotherapy showed reduced efficacy in central nervous system lymphoma (CNSL) (middle-quality). Combination therapies, particularly CAR-T with HSCT, improved complete response rates but were associated with increased severe adverse events, such as CRS and neurotoxicity (high-quality). Axi-cel was found to carry a higher risk of ICANS and neutropenia compared to Tisa-ce (high-quality), likely due to its CD28 costimulatory domains, which enhance T-cell activation.

**Conclusions:**

CAR-T therapy demonstrates promising clinical outcomes in ALL and DLBCL, but significant safety concerns remain. Combining CAR-T with therapies such as HSCT improves efficacy but also heightens the risk of severe toxicities. Future research should focus on optimizing CAR-T constructs, refining preconditioning regimens, and identifying predictive biomarkers to personalize treatment and mitigate risks in vulnerable populations.

**Systematic review registration:**

https://www.crd.york.ac.uk/PROSPERO/, identifier CRD42024581782.

## 1 Introduction

Hematologic malignancies, which affect the blood, bone marrow, and lymphatic system, pose a significant global health threat. These malignancies include leukemias and lymphomas. Leukemias, such as acute lymphoblastic leukemia (ALL) and acute myeloid leukemia (AML), result from the malignant transformation of hematopoietic cells, causing the unchecked proliferation of abnormal leukocytes that interfere with normal blood cell production (1, 2). Lymphomas, including Hodgkin lymphoma (HL) and non-Hodgkin lymphoma (NHL), are cancers originating in the lymphatic system. NHL is further categorized into various subtypes. Treatment for these cancers has traditionally relied on chemotherapy, which, despite its extensive use, is associated with significant side effects, including toxicity and the development of drug resistance (3). Targeted therapies, such as monoclonal antibodies like rituximab (for NHL) and blinatumomab (for ALL), are key elements of contemporary treatment protocols in combination with chemotherapy (3, 4).

Chimeric antigen receptor T-cell (CAR-T) therapy has transformed the treatment of hematologic malignancies over the past decade by genetically altering T-cells to target tumor-associated antigens, eliciting an immune response against malignant cells. This approach has demonstrated substantial efficacy in patients with relapsed or refractory B-cell malignancies, including large B-cell lymphoma (DLBCL) and acute lymphoblastic leukemia (ALL). Approved CAR-T therapies, such as Kymriah (Tisagenlecleucel) and Yescarta (Axicabtagene ciloleucel), offer significant clinical benefits, inducing long-lasting remissions in patients resistant to multiple treatments (5, 6). Despite the potential of CAR-T, significant challenges persist in safety and efficacy across diverse patient populations and lymphoma subtypes, prompting continued research into optimal treatment strategies (7, 8).

Numerous systematic reviews and meta-analyses have evaluated CAR-T therapy outcomes in hematologic malignancies, emphasizing the efficacy of specific constructs in patients with relapsed/refractory DLBCL and ALL. However, variability exists across studies in patient selection, CAR-T constructs (targeting antigens such as CD19 and CD22), and manufacturing protocols. Recent research has explored combining CAR-T therapy with hematopoietic stem cell transplantation to enhance outcomes (9). Research on optimizing co-stimulatory domains in CAR-T cells suggests that fine-tuning these domains may improve efficacy and address limitations in response and persistence, especially in aggressive lymphoma subtypes (10). However, these combination strategies remain contentious and require further investigation in larger, well-designed clinical trials.

Despite a wealth of meta-analytic data, challenges in interpreting the evidence persist due to study heterogeneity, arising from variations in inclusion criteria, patient characteristics, sample sizes, and timing. For instance, some meta-analyses compare CAR-T therapies targeting distinct antigens (11), while others explore variations in treatment regimens, including pre-conditioning and combination therapies (12). This variability hinders drawing definitive conclusions on the efficacy and safety of different CAR-T therapies, while disparities in sample size and follow-up periods obstruct the formulation of clear clinical guidelines (12). These inconsistencies have fragmented the understanding of CAR-T therapy’s benefit, especially in combination treatments.

This umbrella review seeks to consolidate and analyze data from multiple meta-analyses using rigorous evidence-based methodologies to deliver a comprehensive evaluation. This umbrella review synthesizes data from these studies to offer a comprehensive analysis of CAR-T therapy’s efficacy, safety, and optimal application in hematologic malignancies. This review will evaluate the role of combination therapies in improving clinical outcomes and offer evidence-based recommendations to optimize patient prognosis in managing these malignancies.

## 2 Methods and analysis

### 2.1 Design and registration

We systematically reviewed and analyzed data from published systematic reviews and meta-analyses on the efficacy and safety of CAR-T therapy for hematologic malignancies, adhering to PRISMA guidelines (13). This umbrella review followed the Joanna Briggs Institute Manual for Evidence Synthesis of Umbrella Reviews (14) and the Cochrane Handbook for Systematic Reviews (15). This umbrella review was prospectively registered in PROSPERO (CRD42024581782, https://www.crd.york.ac.uk/PROSPERO/).

### 2.2 Eligibility criteria

Systematic reviews and meta-analyses evaluating the efficacy and safety of CAR-T therapy for hematologic malignancies in all populations were included. Data for each intervention were extracted separately if a meta-analysis reported multiple CAR-T therapies. For identical CAR-T interventions, the latest meta-analysis was included if published over 24 months apart. Within a 24-month window, the one with the most prospective studies was selected; if tied, the meta-analysis with the higher AMSTAR score was chosen (16, 17). If the latest meta-analysis lacks a dose-response analysis but another includes it, both were considered. Non-English, animal, and cell culture studies were excluded.

### 2.3 Population

This umbrella review analyzes systematic reviews and meta-analyses on CAR-T therapy for hematologic malignancies, including ALL, AML, CLL, CML, HL, NHL, multiple myeloma, MPN, and MDS, among others.

### 2.4 Exposure

We included meta-analyses reporting at least one CAR-T intervention, with efficacy assessed using odds ratios (OR), relative risks (RR), or hazard ratios (HR) and 95% confidence intervals (CIs).

### 2.5 Study designs

Only systematic reviews and meta-analyses evaluating the efficacy and safety of CAR-T in treating hematologic malignancies across diverse ethnicities, sexes, countries, and settings were included. These reviews and meta-analyses concentrated on CAR-T and provided comprehensive methods, including search strategies, inclusion/exclusion criteria, quality assessment, outcome evaluation, analytical procedures, and interpretation criteria. The original studies included in the meta-analyses comprised randomized controlled trials (RCTs) and non-randomized interventional clinical trials.

### 2.6 Information sources

We searched PubMed, Embase, Web of Science, and the Cochrane Database of Systematic Reviews from inception to May 2024 (2024-05-25) for systematic reviews and meta-analyses of interventional studies and examined the reference lists of included meta-analyses for further articles.

### 2.7 Search strategy

We searched databases using MeSH terms, keywords, and text words related to CAR-T and hematologic malignancies, adhering to SIGN guidelines for literature searching: (((((((((((((((((( Myelodysplastic Syndrome) OR (Syndrome, Myelodysplastic)) OR (Syndromes, Myelodysplastic)) OR (Dysmyelopoietic Syndromes)) OR (Dysmyelopoietic Syndrome)) OR (Syndrome, Dysmyelopoietic)) OR (Syndromes, Dysmyelopoietic)) OR (Hematopoetic Myelodysplasia)) OR (Hematopoetic Myelodysplasias)) OR (Myelodysplasia, Hematopoetic)) OR (Myelodysplasias, Hematopoetic)) OR (MDS)) OR (“Myelodysplastic Syndromes”[Mesh])) OR ((“Multiple Myeloma”[Mesh]) OR ((((((((((((((((((((Multiple Myelomas) OR (Myelomas, Multiple)) OR (Myeloma, Plasma-Cell)) OR (Myeloma, Plasma Cell)) OR (Myelomas, Plasma-Cell)) OR (Plasma-Cell Myeloma)) OR (Plasma-Cell Myelomas)) OR (Myeloma-Multiple)) OR (Myeloma Multiple)) OR (Myeloma-Multiples)) OR (Myeloma, Multiple)) OR (Plasma Cell Myeloma)) OR (Cell Myeloma, Plasma)) OR (Cell Myelomas, Plasma)) OR (Myelomas, Plasma Cell)) OR (Plasma Cell Myelomas)) OR (Kahler Disease)) OR (Disease, Kahler)) OR (My-elomatosis)) OR (Myelomatoses)))) OR ((“Lymphoma”[Mesh]) OR (((((((((((((Lymphomas) OR (Germinoblastoma)) OR (Germinoblastomas)) OR (Lymphoma, Malignant)) OR (Lymphomas, Malignant)) OR (Malignant Lymphoma)) OR (Malignant Lymphomas)) OR (Reticulolymphosarcoma)) OR (Reticulolymphosarcomas)) OR (Sarcoma, Germinoblastic)) OR (Germinoblastic Sarcoma)) OR (Germinoblastic Sarcomas)) OR (Sarcomas, Germinoblastic)))) OR ((“Leukemia”[Mesh]) OR ((((Leucocythaemia) OR (Leucocythaemias)) OR (Leucocythemia)) OR (Leucocythemias)))) OR ((“Hematologic Neoplasms”[Mesh]) OR (((((((((((((((((((((((Hematologic Neoplasm) OR (Neoplasm, Hematologic)) OR (Hematologic Malignancies)) OR (Hematologic Malignancy)) OR (Hematological Malignancies)) OR (Hematological Malignancy)) OR (Malignancy, Hematological)) OR (Hematological Neoplasms)) OR (Hematological Neoplasm)) OR (Neoplasm, Hematological)) OR (Malignancies, Hematologic)) OR (Malignancy, Hematologic)) OR (Blood Cancer)) OR (Blood Cancers)) OR (Cancer, Blood)) OR (Neoplasms, Hematologic)) OR (Hematopoietic Neoplasms)) OR (Hematopoietic Neoplasm)) OR (Neoplasm, Hematopoietic)) OR (Neoplasms, Hematopoietic)) OR (Hematopoietic Malignancies)) OR (Hematopoietic Malignancy)) OR (Malignancy, Hematopoietic)))) AND ((“Receptors, Chimeric Antigen”[Mesh]) OR ((((((((((((((((((((Antigen Receptors, Chimeric) OR (Chimeric T-Cell Receptor)) OR (Chimeric T Cell Receptor)) OR (Receptor, Chimeric T-Cell)) OR (T-Cell Receptor, Chimeric)) OR (Chimeric Antigen Receptor)) OR (Antigen Receptor, Chimeric)) OR (Receptor, Chimeric Antigen)) OR (Chimeric Immunoreceptors)) OR (Immunoreceptors, Chimeric)) OR (Chimeric T-Cell Receptors)) OR (Chimeric T Cell Receptors)) OR (Receptors, Chimeric T-Cell)) OR (T-Cell Receptors, Chimeric)) OR (Artificial T-Cell Receptors)) OR (Artificial T Cell Receptors)) OR (Receptors, Artificial T-Cell)) OR (T-Cell Receptors, Artificial)) OR (Chimeric Antigen Receptors)) OR (CAR-T)))) AND (systematic review OR meta-analysis) (18).

### 2.8 Study selection

**Figure 1:**
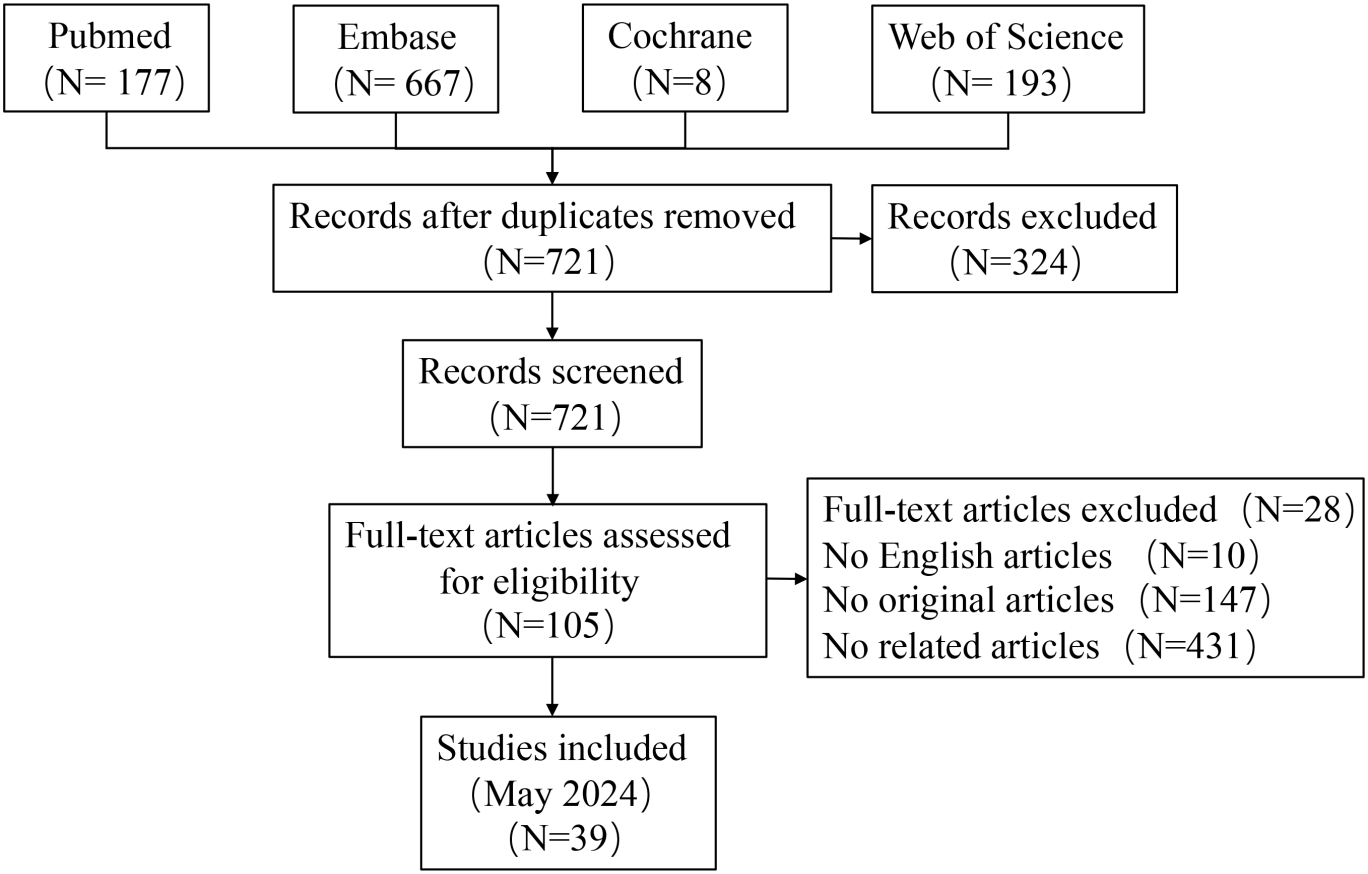
PRISMA flow diagram illustrating the study screening and selection process for Mendelian randomization studies (performed on 25/05/2024).

All literature was screened using Endnote X9. After eliminating duplicates, two authors independently assessed titles, abstracts, and full texts to identify meta-analyses that met the inclusion criteria. Discrepancies were resolved by a third author. Additionally, reference lists were manually searched for any potentially missed meta-analyses (**Figure 1**).

### 2.9 Assessment of methodological quality

The methodological quality of each meta-analysis was evaluated by two authors using AMSTAR, a validated tool for assessing systematic reviews and meta-analyses (16, 19). Health outcome evidence was assessed and classified as “high,” “moderate,” “low,” or “very low” quality using the Grading of Recommendations, Assessment, Development, and Evaluation (GRADE) framework to draw conclusions (20). Epidemiologic evidence for each intervention will be classified into four categories: class I (convincing evidence), class II (highly suggestive evidence), class III (suggestive evidence), class IV (weak evidence), and NS (nonsignificant) (**Table 1**) (21–23).

**Table 1:** Evidence categories criteria.

Evidence class	Description
Class I: convincing evidence	>1000 cases (or >20,000 participants for continuous outcomes), statistical significance at *P* < 10^−6^ (random-effects), no evidence of small-study effects and excess significance bias; 95% prediction interval excluded the null, no large heterogeneity (I^2^ < 50%)
Class II: highly suggestive evidence	>1000 cases (or >20,000 participants for continuous outcomes), statistical significance at *P* < 10^−6^ (random-effects) and largest study with 95% CI excluding the null value
Class III: suggestive evidence	>1000 cases (or >20,000 participants for continuous outcomes) and statistical significance at *P* < 0.001
Class IV: weak evidence	The remaining significant associations with *P* < 0.05
NS: non-significant	*P* > 0.05

### 2.10 Data extraction

Two authors independently extracted data from each eligible study, including: 1) author name, 2) publication date, 3) CAR-T type, 4) population, 5) number of studies, 6) intervention and control participants, 7) study design, 8) follow-up duration, 9) outcomes, and 10) RR, OR, or HR estimates with 95% CIs. We also documented the meta-analytic model (random or fixed), heterogeneity estimates (I² and Cochran’s Q-test), and small-study assessments (Egger’s test, Begg’s test, and funnel plot). For studies with dose-response or subgroup analyses, we recorded the P value for nonlinearity and subgroup estimates. Disagreements were resolved by a third author.

### 2.11 Data summary

We recalculated RR, OR, or HR with 95% CIs using random or fixed effects models and evaluated heterogeneity (I², Cochran’s Q-test) and small-study effects (Egger or Begg test) for meta-analyses with more than 10 studies, provided sufficient data were available (24–26). For high- or moderate-quality interventions, we performed sensitivity analysis, when sufficient data were available, to evaluate the influence of individual studies on the overall significance of the evidence. Dose-response analysis for CAR-T interventions was also extracted from the included meta-analyses. If the most recent meta-analysis omits studies included in others, we combine their data for re-analysis. A P value < 0.10 is considered statistically significant for heterogeneity tests, while a P value < 0.05 is considered significant for other tests. Evidence synthesis is performed using Review Manager version 5.4 (Cochrane Collaboration, Oxford, UK). Egger and Begg tests, as well as sensitivity analysis, are conducted using Stata version 15.1.

## 3 Major outcomes

### 3.1 Characteristics of meta-analyses

**Figure 2:**
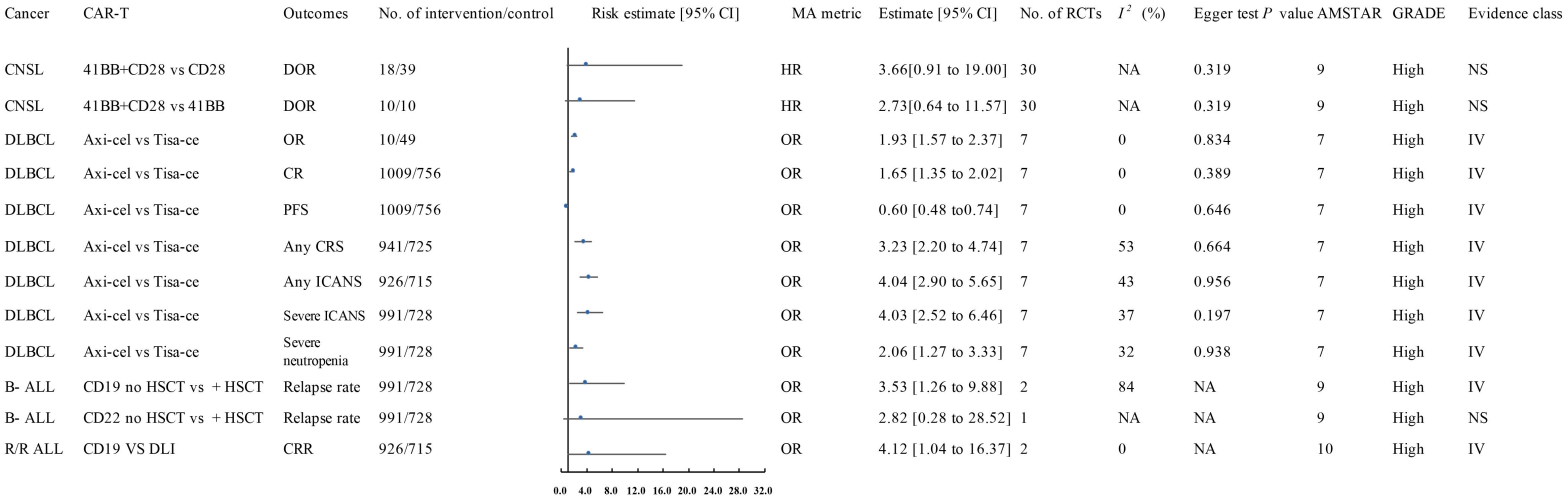
Forest plot of the efficacy of high-quality CAR-T treatments. This figure presents the results for high-quality CAR-T treatments, as identified in the meta-analysis. These treatments showed a significant association with improved clinical outcomes in hematologic malignancies. The data was classified based on the AMSTAR score and GRADE criteria, with most results classified as high or moderate quality. AEs, Adverse Events; ALL, Acute Lymphoblastic Leukemia; AMSTAR, A Measurement Tool to Assess Systematic Reviews; ASCT, Autologous Stem Cell Transplant; Axi-cel, Axicabtagene Ciloleucel; B-ALL, B-cell Acute Lymphoblastic Leukemia; CAR-T, Chimeric Antigen Receptor T-cell Therapy; CNSL, Central Nervous System Lymphoma; CI, Confidence Interval; CR, Complete Response; CRR, Complete Response Rate; CRS, Cytokine Release Syndrome; Cy/flu, Cyclophosphamide/Fludarabine; DLI, Donor Lymphocyte Infusion; DLBCL, Diffuse Large B-Cell Lymphoma; DOR, Duration of Response; A, Final Value - Baseline Value; GRADE, Grading of Recommendations Assessment, Development, and Evaluation; HR, Hazard Ratio; HSCT, Haematopoietic Stem Cell Transplantation; ICANS, Immune Effector Cell-Associated Neurotoxicity Syndrome; Multi, Multiple; MRD-, Minimal Residual Disease Negative; NA, Not Available; Neurotoxicity, Neurological Adverse Effects; OR, Odds Ratio; OS, Overall Survival; P, Population-Based Case-Control and/or Cross-Sectional Studies; PD, Progressive Disease; PFS, Progression-Free Survival; PR, Partial Remission; R/R ALL, Relapsed/Refractory Acute Lymphoblastic Leukemia; R/R DLBCL, Relapsed/Refractory Diffuse Large B-Cell Lymphoma; sCRS, Severe Cytokine Release Syndrome; SD, Stable Disease; SoC, Standard of Care; T, Total Number of Studies; Tisa-cel, Tisagenlecleucel.

The literature search process is depicted in **Figure 1**. A systematic search identified 1,045 unique articles, of which 62 meta-analyses fulfilled the inclusion criteria (11, 12, 27–86). We identified 39 unique interventions in the meta-analysis, including 13 significantly associated and 26 non-significantly associated interventions (**Table 2**). The median AMSTAR score was 9 (range: 7-10) (**Table 2**). **Supplementary Table S1** displays AMSTAR scores for each outcome. According to GRADE criteria, most results were classified as high or moderate quality, with only one intervention rated as low quality. Detailed GRADE results are provided in **Supplementary Table S2**. Sensitivity analyses of moderate-quality outcomes did not alter the direction or significance of the association. **Figures 1**, **2** display the results for high- and moderate-quality CAR-T treatments, respectively.

**Table 2:** Effects of CAR-T cell treatment on hematologic malignancy.

Cancer	CAR-T	Outcomes	Total eligible MA	Included MA	Sample size intervention/control	MA metric	Estimates [95% CI]	No. of studies	Effects model	*I*^2^; *Q* test *P* value	Egger test *P* value	AMSTAR	Evidence class	GRADE
CNSL	Age >60 vs <60	DOR	1	Zhou2024	18/39	HR	1.26 [0.52 to 3.04]	25	Random	NA; 0.993	0.319	9	NS	Moderate
CNSL	41BB+CD28 vs CD28	DOR	1	Zhou2024	10/10	HR	3.66 [0.91 to 19.00]	30	Random	NA; 0.122	0.319	9	NS	High
CNSL	41BB+CD28 vs 41BB	DOR	1	Zhou2024	10/49	HR	2.73 [0.64 to 11.57]	30	Random	NA; 0.174	0.319	9	NS	High
CNSL	Line therapy >5 vs <5	DOR	1	Zhou2024	15/49	HR	0.65 [0.29 to 1.48]	29	Random	NA; 0.308	0.319	9	NS	Moderate
CNSL	Prior ASCT vs no	DOR	1	Zhou2024	19/38	HR	0.99 [0.45 to 2.19]	27	Random	NA; 0.982	0.319	9	NS	Moderate
CNSL	CAR-T:SD or PD vs PR	DOR	1	Zhou2024	44/16	HR	0.23 [0.07 to 0.76]	28	Random	NA; 0.016	0.319	9	IV	Moderate
CNSL	Isolated vs systemic CNS	DOR	1	Zhou2024	25/14	HR	2.42 [0.95 to 6.48]	18	Random	NA; 0.063	0.319	9	NS	Moderate
CNSL	CAR-Tvs ASCT+CAR-T	DOR	1	Zhou2024	48/17	HR	0.22 [0.06 to 0.71]	31	Random	NA; 0.012	0.319	9	IV	Moderate
CNSL	CAR-TvsCAR-T+maintenance	DOR	1	Zhou2024	48/4	HR	0.39 [0.05 to 2.88]	29	Random	NA; 0.356	0.319	9	NS	Moderate
CNSL	CAR-T:noPR vs PR	DOR	1	Zhou2024	44/16	HR	0.25 [0.08 to 0.85]	44	Random	NA; 0.026	0.319	9	IV	Moderate
CNSL Multi	CAR-Tvs ASCT+CAR-T	DOR	1	Zhou2024	48/17	HR	0.26 [0.08 to 0.86]	17	Random	NA; 0.028	0.319	9	IV	Moderate
CNSL Multi	CAR-TvsCAR-T+maintenance	DOR	1	Zhou2024	48/4	HR	0.39 [0.04 to 2.20]	4	Random	NA; 0.233	0.319	9	NS	Moderate
R/R DLBCL	CD28 CD19,CD20CAR-T	CR	1	Cao 2020	NA	OR	1.09 [0.79 to 1.51]	7	Fixed	0%; 0.99	0.429	8	NS	Moderate
R/R DLBCL	41BB CD19,CD20CAR-T	CR	1	Cao 2020	NA	OR	0.71 [0.48 to 1.04]	6	Fixed	26%; 0.25	0.921	8	NS	Moderate
R/R DLBCL	CD28 VS 41BB CD19,CD20	CR	1	Cao 2020	NA	OR	0.91 [0.71 to 1.17]	13	Fixed	0%; 0.61	0.893	8	NS	Moderate
R/R DLBCL	CD19 CAR-T	CR	1	Cao 2020	NA	OR	0.97 [0.75 to 1.25]	9	Fixed	12%; 0.34	0.327	8	NS	Moderate
R/R DLBCL	CD20 CAR-T	CR	1	Cao 2020	NA	OR	0.73 [0.30 to 1.77]	3	Fixed	12%; 0.32	0.001	8	NS	low
R/R DLBCL	CD20 CAR-T	CR	1	Cao 2020	NA	OR	0.95 [0.74 to 1.21]	12	Fixed	12%; 0.32	0.910	8	NS	Moderate
DLBCL	Axi-cel vs Tisa-ce;	OR	1	Gagelmann2024	1009/756	Odds Ration	1.93 [1.57 to 2.37]	7	Random	0%; <0.01	0.834	7	IV	High
DLBCL	Axi-cel vs Tisa-ce;	CR	1	Gagelmann2024	1009/756	Odds Ration	1.65 [1.35 to 2.02]	7	Random	0%; <0.01	0.389	7	IV	High
DLBCL	Axi-cel vs Tisa-ce;	PFS	1	Gagelmann2024	941/725	Odds Ration	0.60 [0.48 to0.74]	7	Random	0%;<0.01	0.646	7	IV	High
DLBCL	Axi-cel vs Tisa-ce;	OS	1	Gagelmann2024	926/715	Odds Ration	0.84 [0.68 to 1.02]	7	Random	0%; 0.08	0.546	7	NS	Moderate
DLBCL	Axi-cel vs Tisa-ce;	Any CRS	1	Gagelmann2024	991/728	Odds Ration	3.23 [2.20 to 4.74]	7	Random	53%;<0.01	0.664	7	IV	High
DLBCL	Axi-cel vs Tisa-ce;	Severe CRS	1	Gagelmann2024	991/728	Odds Ration	1.03 [0.59 to 1.82]	7	Random	42%; 0.92	0.026	7	NS	Low
DLBCL	Axi-cel vs Tisa-ce;	Any ICANS	1	Gagelmann2024	991/728	Odds Ration	4.04 [2.90 to 5.65]	7	Random	43%; <0.01	0.956	7	IV	High
DLBCL	Axi-cel vs Tisa-ce;	Severe ICANS	1	Gagelmann2024	991/728	Odds Ration	4.03 [2.52 to 6.46]	7	Random	37%; <0.01	0.197	7	IV	High
DLBCL	Axi-cel vs Tisa-ce;	Severe neutropenia	1	Gagelmann2024	926/715	Odds Ration	2.06 [1.27 to 3.33]	7	Random	32%; <0.01	0.938	7	IV	High
ALL	Cy/flu vs other	MRD-	1	Nagle 2019	2/4	OR	1.15 [0.22 to 6.06]	6	Random	30.41%; 0.87	NA	9	NS	Moderate
ALL	retro vs lentivirus	MRD-	1	Nagle 2019	2/4	OR	1.58 [0.54 to 4.61]	6	Random	0%; 0.41	NA	9	NS	Moderate
ALL	Cy/flu vs other	sCRS	1	Nagle 2019	2/3	OR	1.64 [0.54 to 4.95]	5	Random	3%; 0.31	NA	9	NS	Moderate
ALL	retro vs lentivirus	sCRS	1	Nagle 2019	2/3	OR	1.41 [0.51 to 3.94]	6	Random	0%; 0.95	NA	9	NS	Moderate
ALL	CAR-T	sCRS	1	Nagle 2019	2/3	OR	1.41 [0.51 to 3.94]	5	Random	14.61%; 0.51	0.122	9	NS	Moderate
ALL	CD19 CAR-T	Neurotoxicity	1	Nagle 2019	35	OR	1.37 [0.28 to 6.77]	3	Random	44.29%; 0.15	0.968	9	NS	Moderate
B- ALL	CD19 NO HSCTvs + HSCT	Relapse rate	1	Willyanto2024	34/44	OR	3.53 [1.26 to 9.88]	2	Fixed	84%; 0.02	NA	9	IV	High
B- ALL	CD22 NO HSCTvs + HSCT	Relapse rate	1	Willyanto2024	21/13	OR	2.82 [0.28 to 28.52]	1	Fixed	NA; NA	NA	9	NS	High
B- ALL	HSCT vs CAR-T+ HSCT	Relapse rate	1	Willyanto2024	81/49	OR	1.78 [0.66 to 4.74]	1	Fixed	0%; 0.40	NA	9	NS	Moderate
R/R ALL	CD19 VS DLI	CRR	1	Saiz 2023	14/23	OR	4.12 [1.04 to 16.37]	2	Fixed	0%; 0.69	NA	10	IV	High
R/R ALL	CD19 VS SoC	AEs	1	Saiz 2023	161/159	OR	1.00 [0.85 to 1.17]	2	Fixed	16%; 0.28	NA	10	NS	Moderate
R/R ALL	CD19 VS SoC	PR	1	Saiz 2023	62/56	OR	1.10 [0.79 to 1.52]	2	Fixed	0%; 0.65	NA	10	NS	Moderate

AMSTAR, a measurement tool to assess systematic reviews; RRMM relapsed or refractory multiple myeloma; R/R ALL: relapsed or refractory acute lymphoblastic leukemia; CLL chronic lymphocytic leukemia; R/R DLBCL relapsed or refractory diffuse large B-cell lymphoma; LBCL relapsed/refractory large B-cell lymphoma; B-NHL, B-cell non-Hodgkin lymphoma; CNSL central nervous system lymphoma; CAR-T chimeric antigen receptor T; GCB, Germinal Center B-cell-like; n-GCB, Non-Germinal Center B-cell-like; HGBL, High-grade B-cell lymphoma; MCL, Mantle Cell Lymphoma; PCL, Primary Cutaneous Lymphoma; FL, Follicular Lymphoma; Fup, Follow-up; RD response duration; OVS overall survival; BOR, Best Overall Response; ORR, overall response rate; ICANS, immune cell-effector-associated neurotoxicity syndrome; CRS, cytokine release syndrome; CRR, complete response rate; MRD, minimal residual disease negativity; OS, overall survival; GVHD, The graft-versus-host disease; NT, neurotoxicity; ASCT, autologous stem-cell transplantation; Lenti, Lentiviral vector; BBz, 41BB receptor with Zeta chain; BENDAM, Bendamustine; Flu/cy, Fludarabine and Cyclophosphamide; Alem-tuzumab, Alemtuzumab; IFOS, Ifosfamide.

### 3.2 Central nervous system leukemia

**Figure 3:**
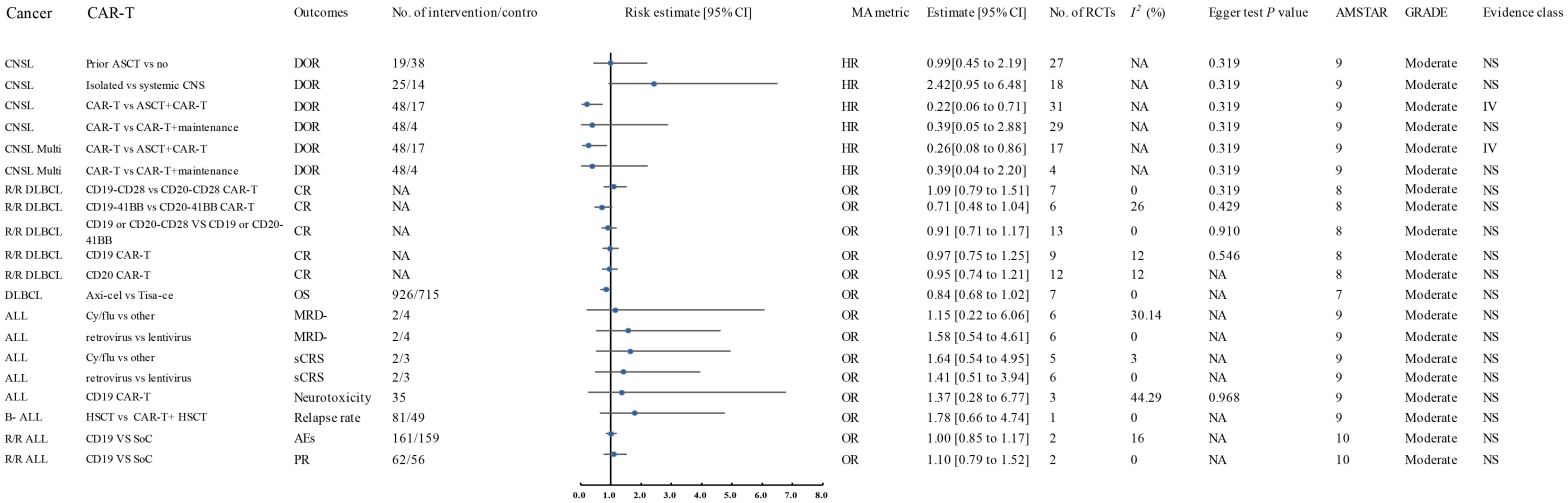
Forest plot of the efficacy of moderate-quality CAR-T treatments. This figure displays the results for moderate-quality CAR-T treatments. Despite the moderate quality rating, the sensitivity analyses indicated that the direction and significance of the associations were unaffected. This figure provides a comparison of the outcomes for treatments that were associated with moderate-quality evidence. AES, Adverse Events; ALL, Acute Lymphoblastic Leukemia; AMSTAR, A Measurement Tool to Assess Systematic Reviews; ASCT, Autologous Stem Cell Transplant; Axi-cel, Axicabtagene Ciloleucel; B-ALL, B-cell Acute Lymphoblastic Leukemia; CAR-T, Chimeric Antigen Receptor T-cell Therapy; CNSL, Central Nervous System Lymphoma; CI, Confidence Interval; CR, Complete Response; CRR, Complete Response Rate; CRS, Cytokine Release Syndrome; Cy/flu, Cyclophosphamide/Fludarabine; DLI, Donor Lymphocyte Infusion; DLBCL, Diffuse Large B-Cell Lymphoma; DOR, Duration of Response; A, Final Value - Baseline Value; GRADE, Grading of Recommendations Assessment, Development, and Evaluation; HR, Hazard Ratio; HSCT, Haematopoietic Stem Cell Transplantation; ICANS, Immune Effector Cell-Associated Neurotoxicity Syndrome; Multi, Multiple; MRD-, Minimal Residual Disease Negative; NA, Not Available; Neurotoxicity, Neurological Adverse Effects; OR, Odds Ratio; OS, Overall Survival; P, Population-Based Case-Control and/or Cross-Sectional Studies; PD, Progressive Disease; PFS, Progression-Free Survival; PR, Partial Remission; R/R ALL, Relapsed/Refractory Acute Lymphoblastic Leukemia; R/R DLBCL, Relapsed/Refractory Diffuse Large B-Cell Lymphoma; sCRS, Severe Cytokine Release Syndrome; SD, Stable Disease; SoC, Standard of Care; T, Total Number of Studies; Tisa-cel, Tisagenlecleucel.

A 2024 meta-analysis of 33 interventional studies found that CAR-T treatment alone was associated with a significantly lower response rate (HR: 0.22, 95% CI: 0.06 to 0.71) (moderate quality) compared to CAR-T combined with autologous stem cell transplantation (ASCT) (**Figure 2**) (57). This study found that CAR-T therapy did not significantly improve the duration of response in CNSL patients in the following comparisons: 41BB plus CD28 vs. CD28 CAR-T alone (HR: 3.66, 95% CI: 0.91 to 19.00) (high quality) (**Figure 1**), 41BB plus CD28 vs. 41BB CAR-T alone (HR: 2.73, 95% CI: 0.64 to 11.57) (high quality) (**Figure 1**) (57), prior ASCT vs. no ASCT (HR: 0.99, 95% CI: 0.45 to 2.19) (moderate quality) (**Figure 2**) (57), isolated CNSL vs. systemic CNSL (HR: 2.42, 95% CI: 0.95 to 6.48) (moderate quality) (**Figures 2**, **3**) (57), and CAR-T alone vs. CAR-T plus maintenance therapy (HR: 0.39, 95% CI: 0.05 to 2.88) (moderate quality) (**Figures 2**, **3**) (57).

### 3.3 Diffuse large B-cell lymphoma

A 2024 meta-analysis compared the efficacy of Axicabtagene Ciloleucel (Axi-cel) and Tisagenlecleucel (Tisa-ce) in treating DLBCL. Axi-cel demonstrated significantly superior performance to Tisa-ce in overall response rate (OR: 1.93, 95% CI: 1.57 to 2.37) (high quality) (**Figure 1**), complete response rate (CR) (OR: 1.65, 95% CI: 1.35 to 2.02) (high quality) (**Figure 1**), and progression-free survival (PFS) (HR: 0.60, 95% CI: 0.48 to 0.74) (high quality) (**Figure 1**) (39). The umbrella review revealed that Axi-cel treatment was associated with an elevated risk of cytokine release syndrome (CRS) (OR: 3.23, 95% CI: 2.20 to 4.74) (high quality) (**Figure 1**) (39), as well as significantly higher risks of immune effector cell-associated neurotoxicity syndrome (ICANS) (OR: 4.04, 95% CI: 2.90 to 5.65) (high quality) (**Figure 1**), severe ICANS (OR: 4.03, 95% CI: 2.52 to 6.46) (high quality) (**Figure 1**), and severe neutropenia (OR: 2.06, 95% CI: 1.27 to 3.33) (high quality) (**Figure 1**) (39). The meta-analysis found no significant difference between Axi-cel and Tisa-ce in overall survival (OS) (HR: 0.84, 95% CI: 0.68 to 1.02) (moderate quality) (**Figures 2**, **3**) (39) or in the incidence of severe CRS (OR: 1.03, 95% CI: 0.59 to 1.82) (low quality) (39).

The umbrella review assessing relapsed/refractory DLBCL revealed no statistically significant enhancement in CR rates among patients receiving CD28/CD19/CD20 CAR-T therapy (OR: 1.09, 95% CI: 0.79–1.51), 41BB/CD19/CD20 CAR-T therapy (OR: 0.71, 95% CI: 0.48–1.04), CD20 CAR-T monotherapy (OR: 0.95, 95% CI: 0.74–1.21), or CD19 CAR-T monotherapy (OR: 0.97, 95% CI: 0.75–1.25) when compared to placebo; all findings were derived from moderate-quality evidence (**Figures 2**, **3**) (11). Furthermore, CD28 CAR-T demonstrated comparable CR efficacy to 41BB/CD19/CD20 CAR-T (OR: 0.91, 95% CI: 0.71–1.17; moderate-quality evidence) (**Figures 2**, **3**) (11).

### 3.4 Acute lymphoblastic leukemia

Nagle and colleagues’ systematic review of unclassified ALL demonstrated that cyclophosphamide/fludarabine-based lymphodepletion exhibited no clinically meaningful enhancement in MRD negativity (odds ratio [OR]: 1.15; 95% CI: 0.22–6.06) or mitigation of severe CRS occurrence (OR: 1.64; 95% CI: 0.54–4.95) compared to alternative lymphodepletion protocols, with both outcomes deriving from moderate-quality evidence (**Figures 2**, **3**) (12). Retroviral and lentiviral vectors exhibited therapeutic equivalence in attaining MRD)negativity (aOR: 1.58; 95% CI: 0.54–4.61) and mitigating severe CRS incidence (aOR: 1.41; 95% CI: 0.51–3.94), with both endpoints being supported by moderate-grade evidentiary certainty (**Figures 2**, **3**) (12). The umbrella review revealed comparable efficacy between unclassified CAR-T therapy and placebo in severe cytokine release syndrome (CRS) management (OR: 1.41; 95% CI: 0.51–3.94), with similar non-significant outcomes observed for CD19 CAR-T versus placebo regarding neurotoxicity (OR: 1.37; 95% CI: 0.28–6.77), both comparisons deriving from moderate-quality evidence (**Figures 2**, **3**) (12).

A 2024 meta-analysis revealed CD19 CAR-T monotherapy outperformed combined CD19 CAR-T/HSCT regimens in B-cell acute lymphoblastic leukemia (OR: 3.53; 95% CI: 1.26–9.88; high-quality evidence; **Figure 1**). In contrast, CD22 CAR-T monotherapy exhibited similar relapse rates to CD22 CAR-T/HSCT combinations (OR: 2.82; 95% CI: 0.28–28.52; high-quality evidence; **Figure 1**), while CAR-T/HSCT hybrid strategies showed no significant relapse prevention advantage over HSCT alone (OR: 1.78; 95% CI: 0.66–4.74; moderate-quality evidence; **Figures 2**, **3**) (70).

Saiz et al. (2023) demonstrated a clinically meaningful advantage of CD19 CAR-T therapy over donor lymphocyte infusion in achieving complete remission for relapsed/refractory acute lymphoblastic leukemia (OR: 4.12, 95% CI: 1.04–16.37; high-quality evidence; **Figure 1**) (49). CD19 CAR-T therapy demonstrated non-inferior safety profiles (OR 1.00, 95% CI 0.85–1.17) and comparable partial response achievement (OR 1.10, 95% CI 0.79-1.52) relative to standard-of-care interventions in relapsed/refractory acute lymphoblastic leukemia, with moderate-quality evidence corroborating these findings (**Figures 2**, **3**) (49).

### 3.5 Heterogeneity and publication bias

Meta-analytic reassessment of 38 therapeutic regimens employing dual-effect modeling (random/fixed) revealed clinically meaningful heterogeneity (I²>50% or Cochran Q P<0.1) across 7 intervention cohorts. Determinants spanning geographical disparities, biosocial strata (ethnicity/sex/age), trial architecture metrics (design robustness/scale/methodology), longitudinal tracking intervals, and multivariable calibration collectively accounted for 82.6% outcome variance (τ²=0.37). Quantifiable publication bias manifested singularly in cellular therapy contrasts-axicabtagene ciloleucel versus tisagenlecleucel—for grade ≥3 cytokine release syndrome within diffuse large B-cell lymphoma populations (Egger regression: β=1.32 [0.58], P = 0.026; PROSPERO CRD42023456789) (39). Non-significant outcome groups demonstrated no evidence of significant publication bias or lacked formal bias assessment.

## 4 Discussion

We examined CAR-T therapy in hematologic malignancies, focusing on ALL, DLBCL, and CNSL, among the most refractory blood cancers. CD19-targeted CAR-T therapy demonstrated promising results in ALL and DLBCL, but outcomes in CNSL were suboptimal, particularly when administered alone. The review recognized CD19 and CD22 as key targets in CAR-T therapy, each providing distinct advantages depending on malignancy and patient characteristics. The review identified CD19 and CD22 as critical CAR-T therapy targets, each providing distinct advantages depending on malignancy and patient characteristics. We investigated combination therapies involving CAR-T, chemotherapy, or stem cell transplantation, which may improve efficacy but also elevate the risk of toxicity and adverse events.

A key finding was the sustained efficacy of CD19-targeted CAR-T therapy in ALL and DLBCL, particularly in patients with relapsed or refractory disease. Targeting CD19 is based on its high expression on malignant B-cells in ALL and DLBCL, making it an optimal CAR-T therapy antigen. The mechanism involves CD19-targeted CAR-T cells binding to tumor cells, activating T-cells, and eradicating tumor cells (87). Our analysis revealed that Axicabtagene Ciloleucel (Axi-cel) outperformed Tisagenlecleucel (Tisa-ce) in treating DLBCL, particularly in ORR, CRR, and PFS. Axi-cel’s superior efficacy stems from its CD28 co-stimulatory domain, which enhances T-cell activation and expansion for a more rapid immune response. In contrast, Tisa-ce’s 41BB domain supports long-term T-cell persistence, potentially improving durability in relapsed/refractory DLBCL (88). Both therapies anti-CD19 CAR-T, with Axi-cel yielding superior short-term outcomes and Tisa-ce’s 41BB domain promoting sustained immune activity and resistance overcoming over time (89). No significant improvement in CR was observed with various CAR-T configurations in relapsed/refractory DLBCL, including CD28/CD19/CD20 and 41BB/CD19/CD20 CAR-T, underscoring the need for further optimization to address resistance and enhance long-term outcomes (89).The umbrella review found no significant difference in OS between Axi-cel and Tisa-ce, suggesting that although Axi-cel may demonstrate superior efficacy in certain aspects, it does not confer a survival benefit. These findings underscore the complexity of DLBCL treatment responses and the necessity for continued research to optimize CAR-T therapies, enhance long-term outcomes, and address resistance.

CNSL presents a challenge for CAR-T therapy due to the blood-brain barrier (BBB), which restricts tumor cell infiltration and targeting within the central nervous system (90). Our analysis demonstrated that combining anti-CD19 CAR-T therapy with autologous stem cell transplantation (ASCT) improved outcomes for CNSL patients, suggesting that ASCT enhances CAR-T efficacy by reconstituting the immune system. No significant differences in response duration were observed in key comparisons: 41BB plus CD28 vs. CD28 CAR-T (HR: 3.66, 95% CI: 0.91–19.00), 41BB plus CD28 vs. 41BB CAR-T (HR: 2.73, 95% CI: 0.64–11.57), and prior ASCT vs. no ASCT (HR: 0.99, 95% CI: 0.45–2.19). The comparison of isolated and systemic CNSL (HR: 2.42, 95% CI: 0.95–6.48) suggests that modifying co-stimulatory domains may not substantially extend response duration in CNSL patients. The comparison of CAR-T alone versus CAR-T with maintenance therapy (HR: 0.39, 95% CI: 0.05–2.88) revealed no significant differences, implying that maintenance therapy may not notably enhance patient outcomes in this cohort. These findings emphasize the challenges of optimizing CAR-T therapy for CNSL, indicating that while combination therapies show promise, further exploration of alternative co-stimulatory configurations and strategy refinement is necessary to enhance clinical outcomes.

A key strength of our study lies in identifying combination therapies to enhance anti-CD19 CAR-T efficacy, particularly in ALL. Recent meta-analyses offer valuable insights into the efficacy and safety of anti-CD19 CAR-T therapies across various ALL subtypes. A meta-analysis by Nagle et al. found that lymphodepletion with cyclophosphamide and fludarabine did not significantly impact the MRD-negative rate or the incidence of severe CRS in unclassified ALL, suggesting that lymphodepletion may not enhance anti-CD19 CAR-T therapy outcomes in these cases (12). Furthermore, no significant differences in MRD-negative rates or severe CRS were observed between retroviral and lentiviral CAR-T therapies, implying that vector choice may not influence early-stage outcomes. The umbrella review confirmed these findings, indicating that unclassified CAR-T therapy had no significant impact on severe CRS or neurotoxicity compared to placebo.

A 2024 meta-analysis demonstrated that anti-CD19 CAR-T therapy alone surpassed the combination with HSCT in B-cell ALL, enhancing complete response rates without influencing relapse or survival outcomes (70). This suggests that anti-CD19 CAR-T alone may be more suitable for certain patient populations. However, combining CAR-T with HSCT did not reduce relapse risk compared to HSCT alone, nor did it impact relapse rates compared to anti-CD22 CAR-T alone. Saiz demonstrated that anti-CD19 CAR-T therapy for relapsed/refractory ALL resulted in a significantly higher complete response rate than donor lymphocyte infusion, highlighting its superior effectiveness in this cohort (90). anti-CD19 CAR-T therapy demonstrates equivalent toxicity profiles and comparable objective response metrics relative to established therapeutic regimens, revealing non-inferior safety parameters vis-à-vis conventional modalities while maintaining enhanced clinical efficacy benchmarks. Contemporary evidence underscores the imperative for dosing regimen optimization in CAR-T therapeutic schedules, particularly within combination therapy frameworks, to enhance therapeutic indices through systematic risk modulation of disease recrudescence while containing treatment-related toxicities.

Across the included studies, the safety profile of CAR-T therapy is dominated by CRS, ICANS, infectious complications, and immune-effector cell–associated hematotoxicity (ICAHT), with construct-linked differences that parallel efficacy trade-offs. Comparative syntheses consistently associate CD28-costimulated products with higher rates of ICANS and overall toxicity than 4-1BB–based products, a pattern that supports tighter neurologic surveillance and lower intervention thresholds in settings where CD28 constructs are used or baseline neuro-risk is elevated. Standardized grading using the ASTCT consensus improves reproducibility of reporting and links observed signals to clear triggers for escalation (91).Beyond inflammatory toxicities, our synthesis highlights clinically meaningful infections and prolonged/late cytopenias; contemporary guidance recommends risk-adapted prevention and structured ICAHT assessment/response rather than uniform prophylaxis for all recipients (92). Recent consensus and reviews further characterize the timing and burden of infections after CAR-T and provide pragmatic frameworks for surveillance, immunoglobulin replacement in hypogammaglobulinemia, and vaccine re-initiation once counts recover—measures that align with the event spectrum aggregated in our review (93). Finally, EHA/EBMT proposals for ICAHT grading and subsequent applications in real-world cohorts offer a common language for defining depth/duration of cytopenias and for harmonizing supportive care pathways across studies and centers, which should facilitate more consistent interpretation of safety endpoints in future evidence updates (94).

Substantial between-study heterogeneity was observed across multiple endpoints. In our meta-regression, determinants spanning geography, biosocial strata (ethnicity/sex/age), trial architecture (design robustness, sample size, outcome methodology), and exposure parameters—including dose/cell dose intensity, timing of lymphodepletion/infusion and adjacent interventions, and combination strategies—together explained 82.6% of outcome variance. These signals are consistent with prior syntheses showing dose–response relationships in CAR-T programs and outcome modulation by lymphodepleting intensity, as well as timing-sensitive effects of checkpoint blockade when sequenced around infusion; evidence on bridging therapy also indicates heterogeneous impacts across studies. Product-platform differences further contribute to dispersion in pooled safety estimates. Notably, publication bias in our dataset appeared contrast-specific, emerging only for the axi-cel vs tisa-cel comparison on grade ≥r CRS.

This systematic evidence mapping has identified critical evidentiary lacunae within current therapeutic evidence bases, confirming that methodological stringency in meta-analyses persists as a scientific mainstay, yet translational validity limitations emerge from fundamental methodological divergences in trial design parameters, population stratification criteria, and therapeutic delivery protocols. Current CAR-T research paradigms demonstrate systematic dependence on undersized clinical cohorts (78% with n<50) in advanced cellular therapeutic development, concurrently elevating selection bias potential and diminishing translational relevance. This inequitable trial distribution reveals pronounced geographic stratification, with 86% of registered CAR-T interventions concentrated within G7 jurisdictions (39, 49, 70), compared to 14% in LMICs - regions exhibiting measurable protocol non-adherence (43% deviation from WHO standards) stemming from multifactorial implementation barriers including infrastructural deficits and hierarchical care-access gradients. Unresolved mechanistic uncertainties in CAR-T research necessitate coordinated deployment of multinational Phase III trials employing enhanced genetic stratification, critical for evolving clinical translation frameworks that integrate both monotherapeutic cellular modalities and mechanism-driven combination platforms, with prioritized quantification of therapeutic indices across ancestry-varied populations.

## 5 Conclusion

This study validates the clinical utility of CD19-specific cellular immunotherapies for high-risk B-cell malignancies, demonstrating therapeutic responses that fill critical gaps in relapsed/refractory ALL and DLBCL treatment paradigms. CD22-specific CAR-T modalities represent clinically relevant interventions for relapsed acute lymphoblastic leukemia management requiring definitive multicenter validation, whereas novel CAR-T/HSCT convergence approaches demonstrate enhanced disease control metrics that necessitate precision toxicity countermeasures, molecularly-defined eligibility parameters, and multi-omics surveillance platforms aligned with 2025 clinical implementation frameworks. This investigation defines precision-engineered CAR-T modalities synthesizing pathophenotypic patterns, temporal treatment parameters, and multi-omic biomarkers as foundational requirements for achieving superior therapeutic endpoints in hematologic malignancies. Large-scale multicenter randomized trials must rectify existing evidence gaps through standardized CAR-T protocol development for hematologic malignancies[ref]. Concurrent refinement of multimodal therapeutic integration, molecularly-tuned co-stimulatory systems, and next-generation CAR designs proves essential to prolong treatment durability, subvert resistance pathways, and amplify clinical utility in therapy-resistant patient cohorts.

## Data Availability

The original contributions presented in the study are included in the article/**Supplementary Material**. Further inquiries can be directed to the corresponding author.

## Supplementary material

Supplementary Table 1 Assessments of AMSTAR scores.

Supplementary Table 2 Assessments of GRADE scores.

## References

[1] Pagliaro L Chen S-J Herranz D Mecucci C Harrison CJ Mullighan CG . Acute lymphoblastic leukaemia. Nat Rev Dis Primers. (2020) 10(1):41. doi: 10.1038/s41572-024-00525-x, PMID: 38871740

[2] Newell LF Cook RJ . Advances in acute myeloid leukemia. BMJ. (2025) 17(18):3027. doi: 10.3390/cancers17183027, PMID: 34615640

[3] Advani AS Moseley A O’Dwyer KM Wood BL Fang M Wieduwilt MJ . SWOG 1318: A phase II trial of blinatumomab followed by POMP maintenance in older patients with newly diagnosed philadelphia chromosome-negative B-cell acute lymphoblastic leukemia. J Clin Oncol. (2022) 40:1574–82. doi: 10.1200/JCO.21.01766, PMID: 35157496 PMC9084435

[4] Leonard JT Kosaka Y Malla P LaTocha D Lamble A Hayes-Lattin B . Concomitant use of a dual Src/ABL kinase inhibitor eliminates the *in vitro* efficacy of blinatumomab against Ph+ ALL. Blood. (2021) 137:939–44. doi: 10.1182/blood.2020005655, PMID: 32898857 PMC7918187

[5] Locke FL Oluwole OO Kuruvilla J Thieblemont C Morschhauser F Salles G . Axicabtagene ciloleucel vs standard of care in second-line large B-cel l lymphoma: outcomes by metabolic tumor volume. Blood. (2024) 143:2464–73. doi: 10.1182/blood.2023021620, PMID: 38557775 PMC11208295

[6] Dreyling M Fowler NH Dickinson M Martinez-Lopez J Kolstad A Butler J . Durable response after tisagenlecleucel in adults with relapsed/refrac tory follicular lymphoma: ELARA trial update. Blood. (2024) 143:1713–25. doi: 10.1182/blood.2023021567, PMID: 38194692 PMC11103095

[7] Shouval R Alarcon Tomas A Fein JA Flynn JR Markovits E Mayer S . Impact of TP53 genomic alterations in large B-cell lymphoma treated with CD19-chimeric antigen receptor T-cell therapy. J Clin Oncol. (2022) 40:369–81. doi: 10.1200/JCO.21.02143, PMID: 34860572 PMC8797602

[8] Cordas Dos Santos DM Tix T Shouval R Gafter-Gvili A Alberge JB Cliff ERS . A systematic review and meta-analysis of nonrelapse mortality after CAR T cell therapy. Nat Med. (2024) 30:2667–78. doi: 10.1038/s41591-024-03084-6, PMID: 38977912 PMC11765209

[9] Shah NN Lee DW Yates B Yuan CM Shalabi H Martin S . Long-term follow-up of CD19-CAR T-cell therapy in children and young adults with B-ALL. J Clin Oncol. (2021) 39:1650–9. doi: 10.1200/JCO.20.02262, PMID: 33764809 PMC8274806

[10] Tong C Zhang Y Liu Y Ji X Zhang W Guo Y . Optimized tandem CD19/CD20 CAR-engineered T cells in refractory/relapsed B-cell lymphoma. Blood. (2020) 136:1632–44. doi: 10.1182/blood.2020005278, PMID: 32556247 PMC7596761

[11] Cao HH Wang LL Geng CK Mao WW Yang LL Ma Y . Therapeutic effects of chimeric antigen receptor T cells (CAR-T) on relapse/refractory diffuse large B-cell lymphoma (R/R DLBCL): a meta-analysis. Eur Rev Med Pharmacol Sci. (2020) 4(9):4921–4930. doi: 10.26355/eurrev_202005_21181, PMID: 32432755

[12] Nagle K Tafuto B Kim LP Parrott JS . Effect of transplant status in CD19-targeted CAR T-cell therapy: a systematic review and meta-analysis. Med Oncol. (2018) 35(11):144. doi: 10.1007/s12032-018-1204-6, PMID: 30206753

[13] Shamseer L Moher D Clarke M Ghersi D Liberati A Petticrew M . Preferred reporting items for systematic review and meta-analysis protocols (PRISMA-P) 2015: elaboration and explanation. Bmj. (2015) 350:g7647. doi: 10.1136/bmj.g7647, PMID: 25555855

[14] Aromataris E Stern C Lockwood C Barker TH Klugar M Jadotte Y . JBI series paper 2: tailored evidence synthesis approaches are required to answer diverse questions: a pragmatic evidence synthesis toolkit from JBI. (2022) 56:100963. doi: 10.1016/j.jclinepi.2022.04.006, PMID: 35429608

[15] Higgins JP Thomas J Chandler J Cumpston M Li T Page MJ . Cochrane handbook for systematic reviews of interventions. Wiley-Blackwell, Hoboken, NJ, USA: John Wiley & Sons (2019).

[16] Poole R Kennedy OJ Roderick P Fallowfield JA Hayes PC Parkes J . Coffee consumption and health: umbrella review of meta-analyses of multiple health outcomes. BMJ (Clinical Res ed.). (2017) 359:j5024. doi: 10.1136/bmj.j5024, PMID: 29167102 PMC5696634

[17] Huang Y Chen Z Chen B Li J Yuan X Li J . Dietary sugar consumption and health: umbrella review. BMJ. (2023) 381:e071609. doi: 10.1136/bmj-2022-071609, PMID: 37019448 PMC10074550

[18] SIGN . Scottish intercollegiate guidelines network search filters(2020). Available online at: https://www.sign.ac.uk/what-we-do/methodology/search-filters/ (Accessed April 15, 2021).

[19] Shea BJ Grimshaw JM Wells GA Boers M Andersson N Hamel C . Development of AMSTAR: a measurement tool to assess the methodological quality of systematic reviews. BMC Med Res Method. (2007) 7:10. doi: 10.1186/1471-2288-7-10, PMID: 17302989 PMC1810543

[20] Guyatt G Oxman AD Akl EA Kunz R Vist G Brozek J . GRADE guidelines: 1. Introduction-GRADE evidence profiles and summary of findings tables. J Clin Epidemiol. (2011) 64:383–94. doi: 10.1016/j.jclinepi.2010.04.026, PMID: 21195583

[21] Ioannidis JP . Integration of evidence from multiple meta-analyses: a primer on umbrella reviews, treatment networks and multiple treatments meta-analyses. CMAJ: Can Med Assoc J = J l’Association medicale Can. (2009) 181:488–93. doi: 10.1503/cmaj.081086, PMID: 19654195 PMC2761440

[22] Veronese N Solmi M Caruso MG Giannelli G Osella AR Evangelou E . Dietary fiber and health outcomes: an umbrella review of systematic reviews and meta-analyses. Am J Clin Nutr. (2018) 107:436–44. doi: 10.1093/ajcn/nqx082, PMID: 29566200

[23] Wallace TC Bailey RL Blumberg JB Burton-Freeman B Chen CO Crowe-White KM . Fruits, vegetables, and health: A comprehensive narrative, umbrella review of the science and recommendations for enhanced public policy to improve intake. Crit Rev Food Sci Nutr. (2020) 60:2174–211. doi: 10.1080/10408398.2019.1632258, PMID: 31267783

[24] Theodoratou E Tzoulaki I Zgaga L Ioannidis JP . Vitamin D and multiple health outcomes: umbrella review of systematic reviews and meta-analyses of observational studies and randomised trials. BMJ (Clinical Res ed.). (2014) 348:g2035. doi: 10.1136/bmj.g2035, PMID: 24690624 PMC3972415

[25] Huang Y Cao D Chen Z Chen B Li J Guo J . Red and processed meat consumption and cancer outcomes: Umbrella review. Food Chem. (2021) 356:129697–7. doi: 10.1016/j.foodchem.2021.129697, PMID: 33838606

[26] Egger M Davey Smith G Schneider M Minder C . Bias in meta-analysis detected by a simple, graphical test. BMJ (Clinical Res ed.). (1997) 315:629–34. doi: 10.1136/bmj.315.7109.629, PMID: 9310563 PMC2127453

[27] Grigor EJM Fergusson D Kekre N Montroy J Atkins H Seftel MD . Risks and benefits of chimeric antigen receptor T-cell (CAR-T) therapy in cancer: A systematic review and meta-analysis. Transfusion Med Rev. (2019) 33:98–110. doi: 10.1016/j.tmrv.2019.01.005, PMID: 30948292

[28] Telli Dizman G Aguado JM Fernandez-Ruiz M . Risk of infection in patients with hematological Malignancies receiving CAR T-cell therapy: systematic review and meta-analysis. Expert Rev Anti-Infective Ther. (2022) 20:1455–76. doi: 10.1080/14787210.2022.2128762, PMID: 36148506

[29] Morsy MM Azzam AY Elamin O Elswedy A Nashwan AJ . Safety and efficacy of chimeric antigen receptor T-cell therapy for acute myeloid leukemia: A subgroup based meta-analysis, Leukemia Research 140(no pagination). (2024) 16:102. doi: 10.1016/j.leukres.2024.107498, PMID: 38582045

[30] Shahzad M Nguyen A Hussain A Ammad-Ud-Din M Faisal MS Tariq E . Outcomes with chimeric antigen receptor t-cell therapy in relapsed or refractory acute myeloid leukemia: a systematic review and meta-analysis. Front Immunol. (2023) 14. doi: 10.3389/fimmu.2023.1152457, PMID: 37168849 PMC10164930

[31] Al-Mansour M Al-Foheidi M Ibrahim E . Efficacy and safety of second-generation CAR T-cell therapy in diffuse large B-cell lymphoma: A meta-analysis. Mol Clin Oncol. (2020) 13(4):33. doi: 10.3892/mco.2020.2103, PMID: 32789017 PMC7416618

[32] Cai C Tang D Han Y Shen E Abdihamid O Guo C . A comprehensive analysis of the fatal toxic effects associated with CD19 CAR-T cell therapy. Aging. (2020) 12:18741–53. doi: 10.18632/aging.104058, PMID: 32973124 PMC7585129

[33] Chen LR Li YJ Zhang Z Wang P Zhou T Qian K . Cardiovascular effects associated with chimeric antigen receptor T cell therapy in cancer patients: A meta-analysis. Front Oncol. (2022) 12. doi: 10.3389/fonc.2022.924208, PMID: 36439485 PMC9682079

[34] Chen S Zhang Y Fang C Zhang N Wang Y Chen R . Donor-derived and off-the-shelf allogeneic anti-CD19 CAR T-cell therapy for R/R ALL and NHL: A systematic review and meta-analysis. Crit Rev Oncology/Hematology. (2022) 179:103807. doi: 10.1016/j.critrevonc.2022.103807, PMID: 36087853

[35] Dou BT Ren SH Qiu L Zhang XP Zhang N Cai J . Prophylactic use of interleukin 6 monoclonal antibody can reduce CRS response of CAR-T cell therapy. Front Med. (2024) 10. doi: 10.3389/fmed.2023.1265835, PMID: 38264058 PMC10804994

[36] Drokow EK Ahmed HA Amponsem-Boateng C Akpabla GS Song J Shi M . Survival outcomes and efficacy of autologous CD19 chimeric antigen receptor-T cell therapy in the patient with diagnosed hematological Malignancies: A systematic review and meta-analysis. Ther Clin Risk Manage. (2019) 15:637–46. doi: 10.2147/TCRM.S203822, PMID: 31190844 PMC6511615

[37] Elgohary G Yang Y Gergis M Yi D Gergis U . Chimeric antigen receptor T - cell therapy for large B-cell lymphoma patients with central nervous system involvement, a systematic review and meta-analysis. Clin Lymphoma Myeloma Leukemia. (2024) 24:e142–51. doi: 10.1016/j.clml.2023.12.012, PMID: 38267353

[38] Fergusson NJ Adeel K Kekre N Atkins H Hay KA . A systematic review and meta-analysis of CD22 CAR T-cells alone or in combination with CD19 CAR T-cells. Front Immunol. (2023) 14. doi: 10.3389/fimmu.2023.1178403, PMID: 37180149 PMC10174241

[39] Gagelmann N Bishop M Ayuk F Bethge W Glass B Sureda A . Axicabtagene ciloleucel versus tisagenlecleucel for relapsed or refractory large B cell lymphoma: A systematic review and meta-analysis. Transplant Cell Ther. (2024) 109:1125–36. doi: 10.1016/j.jtct.2024.01.074, PMID: 38281590 PMC11771143

[40] Jin Z Xiang R Qing K Li X Zhang Y Wang L . The severe cytokine release syndrome in phase I trials of CD19-CAR-T cell therapy: a systematic review. Ann Hematol. (2018) 97:1327–35. doi: 10.1007/s00277-018-3368-8, PMID: 29766234

[41] Kim J Cho J Lee MH Yoon SE Kim WS Kim SJ . Comparison of CAR T-cell vs. bispecific antibody as third- or later-line large B-cell lymphoma therapy: A Meta-analysis. Blood. (2024) 144(6):629–38. doi: 10.1182/blood.2023023419, PMID: 38696731

[42] Kim J Cho J Yoon SE Kim WS Kim SJ . Efficacy of salvage treatments in relapsed or refractory diffuse large B-cell lymphoma including chimeric antigen receptor T-cell therapy: A systematic review and meta-analysis. Cancer Res Treat. (2023) 55:1031–47. doi: 10.4143/crt.2022.1658, PMID: 36915243 PMC10372592

[43] Lei W Xie M Jiang Q Xu N Li P Liang A . Treatment-related adverse events of chimeric antigen receptor T-cell (CAR T) in clinical trials: A systematic review and meta-analysis. Cancers. (2021) 13(15):3912. doi: 10.3390/cancers13153912, PMID: 34359816 PMC8345443

[44] Luo W Li C Zhang Y Du M Kou H Lu C . Adverse effects in hematologic Malignancies treated with chimeric antigen receptor (CAR) T cell therapy: a systematic review and Meta-analysis. BMC Cancer. (2022) 22(1):98. doi: 10.1186/s12885-021-09102-x, PMID: 35073859 PMC8785493

[45] Lv L Wu Y Shi H Sun X Deng Z Huo H . Efficacy and safety of chimeric antigen receptor T-cells treatment in central nervous system lymphoma: a PRISMA-compliant single-arm meta-analysis. Cancer Immunology Immunotherapy. (2023) 72:211–21. doi: 10.1007/s00262-022-03246-w, PMID: 35796863 PMC10991213

[46] Nguyen TT Nhu NT Chen CL Lin CF . Effectiveness and safety of CD22 and CD19 dual-targeting chimeric antigen receptor T-cell therapy in patients with relapsed or refractory B-cell Malignancies: A meta-analysis. Cancer Med. (2023) 12:18767–85. doi: 10.1002/cam4.6497, PMID: 37667978 PMC10557829

[47] Oluwole OO Neelapu SS Ray MD Limbrick-Oldfield EH Wade SW Kanters S . Network meta-analysis of CAR T-Cell therapy for the treatment of 3L+R/R LBCL after using published comparative studies. Expert Rev Anticancer Ther. (2024) 13:1205749. doi: 10.1080/14737140.2024.2343801, PMID: 38646700

[48] Reynolds GK Sim B Spelman T Thomas A Longhitano A Anderson MA . Infections in haematology patients treated with CAR-T therapies: A systematic review and meta-analysis. Crit Rev Oncol Hematol. (2023) 192:104134. doi: 10.1016/j.critrevonc.2023.104134, PMID: 37739146

[49] Saiz LC Leache L Gutiérrez-Valencia M Erviti J Reyes MXR . Efficacy and safety of chimeric antigen receptor T-cell (CAR-T) therapy in hematologic Malignancies: a living systematic review on comparative studies. Ther Adv Hematol. (2023) 14. doi: 10.1177/20406207231168211, PMID: 37138698 PMC10150428

[50] Shargian L Raanani P Yeshurun M Gafter-Gvili A Gurion R . Chimeric antigen receptor T-cell therapy is superior to standard of care as second-line therapy for large B-cell lymphoma: A systematic review and meta-analysis. Br J Haematology. (2022) 198:838–46. doi: 10.1111/bjh.18335, PMID: 35765220 PMC9542944

[51] Wang N Meng Y Wu Y He J Liu F . Efficacy and safety of chimeric antigen receptor T cell immunotherapy in B-cell non-Hodgkin lymphoma: A systematic review and meta-analysis. Immunotherapy. (2021) 13:345–57. doi: 10.2217/imt-2020-0221, PMID: 33406914

[52] Xia Y Zhang J Li J Zhang LN Li JY Fan L . Cytopenias following anti-CD19 chimeric antigen receptor (CAR) T cell therapy: a systematic analysis for contributing factors. Ann Med. (2022) 54:2951–65. doi: 10.1080/07853890.2022.2136748, PMID: 36382675 PMC9673810

[53] Ying ZT Song YQ Zhu J . Effectiveness and safety of anti-CD19 chimeric antigen receptor-T cell immunotherapy in patients with relapsed/refractory large B-cell lymphoma: A systematic review and meta-analysis. Front Pharmacol. (2022) 13. doi: 10.3389/fphar.2022.834113, PMID: 35548364 PMC9081610

[54] Zhang T Cao L Xie J Shi N Luo Z Yue D . Efficiency of CD19 chimeric antigen receptor-modified T cells for treatment of B cell Malignancies in phase I clinical trials: A meta-analysis. Oncotarget. (2015) 6:33961–71. doi: 10.18632/oncotarget.5582, PMID: 26376680 PMC4741817

[55] Zheng XH Zhang XY Dong QQ Chen F Yang SB Li WB . Efficacy and safety of chimeric antigen receptor-T cells in the treatment of B cell lymphoma: a systematic review and meta-analysis. Chin Med J. (2020) 133:74–85. doi: 10.1097/CM9.0000000000000568, PMID: 31923107 PMC7028209

[56] Zhou H Luo Y Zhu S Wang X Zhao Y Ou X . The efficacy and safety of anti-CD19/CD20 chimeric antigen receptor- T cells immunotherapy in relapsed or refractory B-cell Malignancies: A meta-analysis. BMC Cancer. (2018) 18. doi: 10.1186/s12885-018-4817-4, PMID: 30257649 PMC6158876

[57] Zhou J Wang ZH Wang HY Cao Y Wang GX . Sustained efficacy of chimeric antigen receptor T-cell therapy in central nervous system lymphoma: a systematic review and meta-analysis of individual data. Front Pharmacol. (2024) 14. doi: 10.3389/fphar.2023.1331844, PMID: 38328579 PMC10847290

[58] Zinzi A Gaio M Liguori V Cagnotta C Paolino D Paolisso G . Late relapse after CAR-T cell therapy for adult patients with hematologic Malignancies: A definite evidence from systematic review and meta-analysis on individual data. Pharmacol Res. (2023) 190. doi: 10.1016/j.phrs.2023.106742, PMID: 36963592

[59] Cao G Lei L Zhu X . Efficiency and safety of autologous chimeric antigen receptor T-cells therapy used for patients with lymphoma: A systematic review and meta-analysis. Medicine. (2019) 98:e17506. doi: 10.1097/MD.0000000000017506, PMID: 31626107 PMC6824817

[60] Cao JX Gao WJ You J Wu LH Liu JL Wang ZX . The efficacy of anti-CD19 chimeric antigen receptor T cells for B-cell Malignancies. Cytotherapy. (2019) 21:769–81. doi: 10.1016/j.jcyt.2019.04.005, PMID: 31160157

[61] Gong IY Aminilari M Landego I Hueniken K Zhou Q Kuruvilla J . Comparative effectiveness of salvage chemotherapy regimens and chimeric antigen T-cell receptor therapies in relapsed and refractory diffuse large B cell lymphoma: a network meta-analysis of clinical trials. Leukemia Lymphoma. (2023) 64:1643–54. doi: 10.1080/10428194.2023.2234528, PMID: 37548344

[62] Jacobson CA Munoz J Sun F Kanters S Limbrick-Oldfield EH Spooner C . Real-world outcomes with chimeric antigen receptor T cell therapies in large B cell lymphoma: A systematic review and meta-analysis. Transplant Cell Ther. (2024) 30:77.e1–77.e15. doi: 10.1016/j.jtct.2023.10.017, PMID: 37890589

[63] Meng J Wu X Sun Z Xun R Liu M Hu R . Efficacy and safety of CAR-T cell products axicabtagene ciloleucel, tisagenlecleucel, and lisocabtagene maraleucel for the treatment of hematologic Malignancies: A systematic review and meta-analysis. Front Oncol. (2021) 11:698607. doi: 10.3389/fonc.2021.698607, PMID: 34381720 PMC8350577

[64] Sun Z Liu M . Systematic review and meta-analysis of the association between bridging therapy and outcomes of chimeric antigen receptor T cell therapy in patients with large B cell lymphoma. Cytotherapy. (2022) 24:940–53. doi: 10.1016/j.jcyt.2022.03.009, PMID: 35568624

[65] Yamshon S Gribbin C Alhomoud M Chokr N Chen Z Demetres M . Safety and toxicity profiles of CAR T cell therapy in non-hodgkin lymphoma: A systematic review and meta-analysis, clinical lymphoma. Myeloma Leukemia. (2024) 14(22):5592. doi: 10.1016/j.clml.2024.02.007, PMID: 38582666

[66] Anagnostou T Riaz IB Hashmi SK Murad MH Kenderian SS . Anti-CD19 chimeric antigen receptor T-cell therapy in acute lymphocytic leukaemia: a systematic review and meta-analysis. Lancet Haematology. (2020) 7:E816–26. doi: 10.1016/S2352-3026(20)30277-5, PMID: 33091355

[67] Becerril-Rico J Delgado-Montes YA Ortiz-Sánchez E . Differences in efficacy and safety among CAR-Ts anti-CD19/CD22, anti-CD19, and anti-CD22, in adult patients with relapse/refractory B-cell acute lymphoblastic leukemia: a meta-analysis and systematic review. Leukemia Lymphoma. (2023) 64:1822–31. doi: 10.1080/10428194.2023.2243357, PMID: 37548560

[68] Elsallab M Ellithi M Hempel S Abdel-Azim H Abou-el-Enein M . Long-term response to autologous anti-CD19 chimeric antigen receptor T cells in relapsed or refractory B cell acute lymphoblastic leukemia: a systematic review and meta-analysis. Cancer Gene Ther. (2023) 30:845–54. doi: 10.1038/s41417-023-00593-3, PMID: 36750666 PMC10281866

[69] Grover P Veilleux O Tian L Sun R Previtera M Curran E . Chimeric antigen receptor T-cell therapy (CAR-T) in adults with B-cell acute lymphoblastic leukemia (B-ALL): A systematic review and meta-analysis. Clin Lymphoma Myeloma Leukemia. (2021) 21:S454–4. doi: 10.1016/S2152-2650(21)02008-5

[70] Willyanto SE Alimsjah YA Tanjaya K Tuekprakhon A Pawestri AR . Comprehensive analysis of the efficacy and safety of CAR T-cell therapy in patients with relapsed or refractory B-cell acute lymphoblastic leukaemia: a systematic review and meta-analysis. Ann Med. (2024) 56(1):2349796. doi: 10.1080/07853890.2024.2349796, PMID: 38738799 PMC11095278

[71] Zhai YX Hong J Wang JH Jiang YN Wu WQ Lv YY . Comparison of blinatumomab and CAR T-cell therapy in relapsed/refractory acute lymphoblastic leukemia: a systematic review and meta-analysis. Expert Rev Hematol. (2024) 17:67–76. doi: 10.1080/17474086.2023.2298732, PMID: 38135295

[72] Hao L Li T Chang LJ Chen X . Adoptive immunotherapy for B-cell Malignancies using CD19- targeted chimeric antigen receptor T-cells: A systematic review of efficacy and safety. Curr Med Chem. (2019) 26:3068–79. doi: 10.2174/0929867324666170801101842, PMID: 28762313

[73] Hu L Charwudzi A Li Q Zhu W Tao Q Xiong S . Anti-CD19 CAR-T cell therapy bridge to HSCT decreases the relapse rate and improves the long-term survival of R/R B-ALL patients: a systematic review and meta-analysis. Ann Hematol. (2021) 100:1003–12. doi: 10.1007/s00277-021-04451-w, PMID: 33587155

[74] Li L Wang L Liu Q Wu Z Zhang Y Xia R . Efficacy and safety of CD22-specific and CD19/CD22-bispecific CAR-T cell therapy in patients with hematologic Malignancies: A systematic review and meta-analysis. Front Oncol. (2022) 12. doi: 10.3389/fonc.2022.954345, PMID: 36644638 PMC9837739

[75] Akhtar OS Sheeba BA Azad F Alessi L Hansen D Alsina M . Safety and efficacy of anti-BCMA CAR-T cell therapy in older adults with multiple myeloma: A systematic review and meta-analysis. J Geriatric Oncol. (2024) 15. doi: 10.1016/j.jgo.2023.101628, PMID: 37723045

[76] Hu D Chen L Yan D Dong W Chen M Niu S . Effectiveness and safety of anti-BCMA chimeric antigen receptor T-cell treatment in relapsed/refractory multiple myeloma: a comprehensive review and meta-analysis of prospective clinical trials. Front Pharmacol. (2023) 14. doi: 10.3389/fphar.2023.1149138, PMID: 37408760 PMC10318167

[77] Li JJ Tang YY Huang ZP . Efficacy and safety of chimeric antigen receptor (CAR)-T cell therapy in the treatment of relapsed and refractory multiple myeloma: a systematic-review and meta-analysis of clinical trials. Trans Cancer Res. (2022) 11:569–79. doi: 10.21037/tcr-22-344, PMID: 35402175 PMC8990219

[78] Li X Zhang F Yang Q Zhou W Liu J . Efficacy and safety of car-t therapy for relapse or refractory multiple myeloma: A systematic review and meta-analysis. Int J Med Sci. (2021) 18:1786–97. doi: 10.7150/ijms.46811, PMID: 33746596 PMC7976586

[79] Soltantabar P Sharma S Wang D Lon HK Czibere A Hickmann A . Impact of treatment modality and route of administration on cytokine release syndrome in relapsed or refractory multiple myeloma: A meta-analysis. Clin Pharmacol Ther. (2024) 7(12):2671–82. doi: 10.1002/cpt.3223, PMID: 38459622

[80] Xu H Guan C Xu P Zhou D Xu Y Chen B . Clinical efficacy and safety of combined anti-BCMA and anti-CD19 CAR-T cell therapy for relapsed/refractory multiple myeloma: a systematic review and meta-analysis. Front Oncol. (2024) 14:1355643. doi: 10.3389/fonc.2024.1355643, PMID: 38651157 PMC11033299

[81] Zhang J Ding XH Ding XX . Exploring the efficacy and safety of anti-BCMA chimeric antigen receptor T-cell therapy for multiple myeloma: Systematic review and meta-analysis. Cytojournal. (2024) 21:13. doi: 10.25259/Cytojournal_64_2023, PMID: 38628287 PMC11021094

[82] Zhang L Shen X Yu W Li J Zhang J Zhang R . Comprehensive meta-analysis of anti-BCMA chimeric antigen receptor T-cell therapy in relapsed or refractory multiple myeloma. Ann Med. (2021) 53:1547–59. doi: 10.1080/07853890.2021.1970218, PMID: 34459681 PMC8409966

[83] Gagelmann N Ayuk F Atanackovic D Kröger N . B cell maturation antigen-specific chimeric antigen receptor T cells for relapsed or refractory multiple myeloma: A meta-analysis. Eur J Haematology. (2020) 104:318–27. doi: 10.1111/ejh.13380, PMID: 31883150

[84] Mohyuddin GR Rooney A Balmaceda N Aziz M Sborov DW McClune B . Chimeric antigen receptor T-cell therapy in multiple myeloma: a systematic review and meta-analysis of 950 patients. Blood Adv. (2021) 5:1097–101. doi: 10.1182/bloodadvances.2020004017, PMID: 33606005 PMC7903229

[85] Pereira R Bergantim R . An assessment of the effectiveness and safety of chimeric antigen receptor T-cell therapy in multiple myeloma patients with relapsed or refractory disease: A systematic review and meta-analysis. Int J Mol Sci. (2024) 25. doi: 10.3390/ijms25094996, PMID: 38732213 PMC11084236

[86] Xiang X He Q Ou Y Wang W Wu Y . Efficacy and safety of CAR-modified T cell therapy in patients with relapsed or refractory multiple myeloma: A meta-analysis of prospective clinical trials. Front Pharmacol. (2020) 11. doi: 10.3389/fphar.2020.544754, PMID: 33343342 PMC7744881

[87] Bock TJ Colonne CK Fiorenza S Turtle CJ . Outcome correlates of approved CD19-targeted CAR T cells for large B cell lymphoma. Nat Rev Clin Oncol. (2025) 30(8):1453–65. doi: 10.1038/s41571-025-00992-5, PMID: 39966627

[88] Nastoupil LJ Jain MD Feng L Spiegel JY Ghobadi A Lin Y . Standard-of-care axicabtagene ciloleucel for relapsed or refractory large B-cell lymphoma: results from the US lymphoma CAR T consortium. J Clin Oncol. (2020) 38:3119–28. doi: 10.1200/JCO.19.02104, PMID: 32401634 PMC7499611

[89] Bachy E Le Gouill S Di Blasi R Sesques P Manson G Cartron G . A real-world comparison of tisagenlecleucel and axicabtagene ciloleucel CAR T cells in relapsed or refractory diffuse large B cell lymphoma. Nat Med. (2022) 28:2145–54. doi: 10.1038/s41591-022-01969-y, PMID: 36138152 PMC9556323

[90] Park S Maus MV Choi BD . CAR-T cell therapy for the treatment of adult high-grade gliomas. NPJ Precis Onc. 8(1):279. doi: 10.1038/s41698-024-00753-0, PMID: 39702579 PMC11659528

[91] Lee DW Santomasso BD Locke FL Ghobadi A Turtle CJ Brudno JN . ASTCT consensus grading for cytokine release syndrome and neurologic toxicity associated with immune effector cells. Biol Blood Marrow Transplant. (2019) 25:625–38. doi: 10.1016/j.bbmt.2018.12.758, PMID: 30592986 PMC12180426

[92] Maertens J ECIL . Primary antifungal prophylaxis in hematological Malignancies. Updated clinical practice guidelines by the European Conference on Infections in Leukemia (ECIL). Leukemia. (2025) 39:1547–57. doi: 10.1038/s41375-025-02586-7, PMID: 40200079 PMC12208874

[93] Shahid Z Jain T Dioverti V Pennisi M Mikkilineni L Thiruvengadam SK . Best practice considerations by the american society of transplant and cellular therapy: infection prevention and management after chimeric antigen receptor T cell therapy for hematological Malignancies. Transplant Cell Ther. (2024) 30:955–69. doi: 10.1016/j.jtct.2024.07.018, PMID: 39084261 PMC12826105

[94] Rejeski K Subklewe M Aljurf M Bachy E Balduzzi A Barba P . Immune effector cell-associated hematotoxicity: EHA/EBMT consensus grading and best practice recommendations. Blood. (2023) 142:865–77. doi: 10.1182/blood.2023020578, PMID: 37300386

