# Supplementary material for: Efficacy and safety of chimeric antigen receptor T-cell in the treatment of hematologic malignancy: an umbrella review of systematic review and meta-analysis
Source: Front Immunol. 2025 Nov 19;16:1608768. doi: 10.3389/fimmu.2025.1608768 (PMC12672455; doi:10.3389/fimmu.2025.1608768)
Supplement: Supplementary Table 2 — Assessments of GRADE scores. [file Table2.docx]

| Supplementary Table S1. Assessments of AMSTAR scores. | | | | | | | | | | | | | | | | |
| --- | --- | --- | --- | --- | --- | --- | --- | --- | --- | --- | --- | --- | --- | --- | --- | --- |
| Cancer | CAR-T | Outcome | Author | Year | A priori design provided | Duplicate study selection & data extraction | At least two electronic databases searched | Status of  publication used as an inclusion criterion | List of  included and excluded studies provided | Characteristics of included  studies provided | Scientific quality of  included studies assessed | Scientific quality of the included studies used  appropriately to form  conclusions | Appropriate methods to  combine studies | Publication bias assessed | Conflict of interest included | Total AMSTAR Score |
| AML | CD33 | ORR | MahmoudM.Morsy | 2024 | 0 | 0 | 1 | 0 | 1 | 1 | 1 | 0 | 1 | 0 | 1 | 6 |
| AML | CD33 | CR | MahmoudM.Morsy | 2024 | 0 | 0 | 1 | 0 | 1 | 1 | 1 | 0 | 1 | 0 | 1 | 6 |
| AML | CD33 | PR | MahmoudM.Morsy | 2024 | 0 | 0 | 1 | 0 | 1 | 1 | 1 | 0 | 1 | 0 | 1 | 6 |
| AML | CD33 | CRS | MahmoudM.Morsy | 2024 | 0 | 0 | 1 | 0 | 1 | 1 | 1 | 0 | 1 | 0 | 1 | 6 |
| AML | CD33 | GVHD | MahmoudM.Morsy | 2024 | 0 | 0 | 1 | 0 | 1 | 1 | 1 | 0 | 1 | 0 | 1 | 6 |
| AML | CD33 | ORR | Moazzam Shahzad | 2023 | 1 | 1 | 1 | 0 | 1 | 1 | 1 | 0 | 1 | 0 | 1 | 8 |
| AML | CD33 | ORR CR | Moazzam Shahzad | 2023 | 1 | 1 | 1 | 0 | 1 | 1 | 1 | 0 | 1 | 0 | 1 | 8 |
| AML | CD33 | CRS | Moazzam Shahzad | 2023 | 1 | 1 | 1 | 0 | 1 | 1 | 1 | 0 | 1 | 0 | 1 | 8 |
| B-cell malignancy | CD30 | overall infection | G. Telli Dizman | 2022 | 1 | 1 | 1 | 0 | 1 | 1 | 1 | 0 | 1 | 0 | 1 | 8 |
| MM | CD30 | overall infection | G. Telli Dizman | 2022 | 1 | 1 | 1 | 0 | 1 | 1 | 1 | 0 | 1 | 0 | 1 | 8 |
| B-cell malignancy | CD30 | grade ≥3 infection | G. Telli Dizman | 2022 | 1 | 1 | 1 | 0 | 1 | 1 | 1 | 0 | 1 | 0 | 1 | 8 |
| MM | CD30 | grade ≥3 infection | G. Telli Dizman | 2022 | 1 | 1 | 1 | 0 | 1 | 1 | 1 | 0 | 1 | 0 | 1 | 8 |
| B-cell malignancy | CD30 | infection-related mortality | G. Telli Dizman | 2022 | 1 | 1 | 1 | 0 | 1 | 1 | 1 | 0 | 1 | 0 | 1 | 8 |
| MM | CD30 | infection-related mortality | G. Telli Dizman | 2022 | 1 | 1 | 1 | 0 | 1 | 1 | 1 | 0 | 1 | 0 | 1 | 8 |
| B-cell malignancy | CD30 | invasive fungal infection | G. Telli Dizman | 2022 | 1 | 1 | 1 | 0 | 1 | 1 | 1 | 0 | 1 | 0 | 1 | 8 |
| MM | CD30 | invasive fungal infection | G. Telli Dizman | 2022 | 1 | 1 | 1 | 0 | 1 | 1 | 1 | 0 | 1 | 0 | 1 | 8 |
| B-cell malignancy | CD30 | viral infection | G. Telli Dizman | 2022 | 1 | 1 | 1 | 0 | 1 | 1 | 1 | 0 | 1 | 0 | 1 | 8 |
| MM | CD30 | viral infection | G. Telli Dizman | 2022 | 1 | 1 | 1 | 0 | 1 | 1 | 1 | 0 | 1 | 0 | 1 | 8 |
| B-cell malignancy | CD30 | bacterial infection | G. Telli Dizman | 2022 | 1 | 1 | 1 | 0 | 1 | 1 | 1 | 0 | 1 | 0 | 1 | 8 |
| MM | CD30 | bacterial infection | G. Telli Dizman | 2022 | 1 | 1 | 1 | 0 | 1 | 1 | 1 | 0 | 1 | 0 | 1 | 8 |
| DLBCL | AUTO CART | 1-year PFS | Jinchul Kim | 2023 | 0 | 1 | 1 | 0 | 1 | 1 | 1 | 0 | 1 | 0 | 1 | 7 |
| DLBCL | AUTO CART | CR | Jinchul Kim | 2023 | 0 | 1 | 1 | 0 | 1 | 1 | 1 | 0 | 1 | 0 | 1 | 7 |
| DLBCL | Chemo | 1-year PFS | Jinchul Kim | 2023 | 0 | 1 | 1 | 0 | 1 | 1 | 1 | 0 | 1 | 0 | 1 | 7 |
| DLBCL | Chemo | CR | Jinchul Kim | 2023 | 0 | 1 | 1 | 0 | 1 | 1 | 1 | 0 | 1 | 0 | 1 | 7 |
| DLBCL | CD19 28Z | Any grades Neutropenia | Wenjing Luo | 2022 | 1 | 0 | 1 | 0 | 1 | 1 | 1 | 0 | 1 | 1 | 1 | 8 |
| DLBCL | CD19 28Z | Any grades thrombocytopenia | Wenjing Luo | 2022 | 1 | 0 | 1 | 0 | 1 | 1 | 1 | 0 | 1 | 1 | 1 | 8 |
| DLBCL | CD19 28Z | Any grades anemia | Wenjing Luo | 2022 | 1 | 0 | 1 | 0 | 1 | 1 | 1 | 0 | 1 | 1 | 1 | 8 |
| DLBCL | CD19 28Z | ≥3 grades Neutropenia | Wenjing Luo | 2022 | 1 | 0 | 1 | 0 | 1 | 1 | 1 | 0 | 1 | 1 | 1 | 8 |
| DLBCL | CD19 28Z | ≥3 grades thrombocytopenia | Wenjing Luo | 2022 | 1 | 0 | 1 | 0 | 1 | 1 | 1 | 0 | 1 | 1 | 1 | 8 |
| DLBCL | CD19 28Z | ≥3 grades anemia | Wenjing Luo | 2022 | 1 | 0 | 1 | 0 | 1 | 1 | 1 | 0 | 1 | 1 | 1 | 8 |
| DLBCL | CD19 41BB | Any grades Neutropenia | Wenjing Luo | 2022 | 1 | 0 | 1 | 0 | 1 | 1 | 1 | 0 | 1 | 1 | 1 | 8 |
| DLBCL | CD19 41BB | Any grades thrombocytopenia | Wenjing Luo | 2022 | 1 | 0 | 1 | 0 | 1 | 1 | 1 | 0 | 1 | 1 | 1 | 8 |
| DLBCL | CD19 41BB | Any grades anemia | Wenjing Luo | 2022 | 1 | 0 | 1 | 0 | 1 | 1 | 1 | 0 | 1 | 1 | 1 | 8 |
| DLBCL | CD19 41BB | ≥3 grades Neutropenia | Wenjing Luo | 2022 | 1 | 0 | 1 | 0 | 1 | 1 | 1 | 0 | 1 | 1 | 1 | 8 |
| DLBCL | CD19 41BB | ≥3 grades thrombocytopenia | Wenjing Luo | 2022 | 1 | 0 | 1 | 0 | 1 | 1 | 1 | 0 | 1 | 1 | 1 | 8 |
| DLBCL | CD19 41BB | ≥3 grades anemia | Wenjing Luo | 2022 | 1 | 0 | 1 | 0 | 1 | 1 | 1 | 0 | 1 | 1 | 1 | 8 |
| DLBCL | CD20 41BB | Any grades Neutropenia | Wenjing Luo | 2022 | 1 | 0 | 1 | 0 | 1 | 1 | 1 | 0 | 1 | 1 | 1 | 8 |
| DLBCL | CD20 41BB | Any grades thrombocytopenia | Wenjing Luo | 2022 | 1 | 0 | 1 | 0 | 1 | 1 | 1 | 0 | 1 | 1 | 1 | 8 |
| DLBCL | CD20 41BB | Any grades anemia | Wenjing Luo | 2022 | 1 | 0 | 1 | 0 | 1 | 1 | 1 | 0 | 1 | 1 | 1 | 8 |
| DLBCL | CD20 41BB | ≥3 grades Neutropenia | Wenjing Luo | 2022 | 1 | 0 | 1 | 0 | 1 | 1 | 1 | 0 | 1 | 1 | 1 | 8 |
| DLBCL | CD20 41BB | ≥3 grades thrombocytopenia | Wenjing Luo | 2022 | 1 | 0 | 1 | 0 | 1 | 1 | 1 | 0 | 1 | 1 | 1 | 8 |
| DLBCL | CD20 41BB | ≥3 grades anemia | Wenjing Luo | 2022 | 1 | 0 | 1 | 0 | 1 | 1 | 1 | 0 | 1 | 1 | 1 | 8 |
| DLBCL | CD19 no lyphodepletion | Any grades Neutropenia | Wenjing Luo | 2022 | 1 | 0 | 1 | 0 | 1 | 1 | 1 | 0 | 1 | 1 | 1 | 8 |
| DLBCL | CD19 no lyphodepletion | Any grades thrombocytopenia | Wenjing Luo | 2022 | 1 | 0 | 1 | 0 | 1 | 1 | 1 | 0 | 1 | 1 | 1 | 8 |
| DLBCL | CD19 no lyphodepletion | Any grades anemia | Wenjing Luo | 2022 | 1 | 0 | 1 | 0 | 1 | 1 | 1 | 0 | 1 | 1 | 1 | 8 |
| DLBCL | CD19 no lyphodepletion | ≥3 grades Neutropenia | Wenjing Luo | 2022 | 1 | 0 | 1 | 0 | 1 | 1 | 1 | 0 | 1 | 1 | 1 | 8 |
| DLBCL | CD19 no lyphodepletion | ≥3 grades thrombocytopenia | Wenjing Luo | 2022 | 1 | 0 | 1 | 0 | 1 | 1 | 1 | 0 | 1 | 1 | 1 | 8 |
| DLBCL | CD19 no lyphodepletion | ≥3 grades anemia | Wenjing Luo | 2022 | 1 | 0 | 1 | 0 | 1 | 1 | 1 | 0 | 1 | 1 | 1 | 8 |
| DLBCL | CD19 lyphodepletion flu/cy+beam | Any grades Neutropenia | Wenjing Luo | 2022 | 1 | 0 | 1 | 0 | 1 | 1 | 1 | 0 | 1 | 1 | 1 | 8 |
| DLBCL | CD19 lyphodepletion flu/cy+beam | Any grades thrombocytopenia | Wenjing Luo | 2022 | 1 | 0 | 1 | 0 | 1 | 1 | 1 | 0 | 1 | 1 | 1 | 8 |
| DLBCL | CD19 lyphodepletion flu/cy+beam | Any grades anemia | Wenjing Luo | 2022 | 1 | 0 | 1 | 0 | 1 | 1 | 1 | 0 | 1 | 1 | 1 | 8 |
| DLBCL | CD19 lyphodepletion flu/cy+beam | ≥3 grades Neutropenia | Wenjing Luo | 2022 | 1 | 0 | 1 | 0 | 1 | 1 | 1 | 0 | 1 | 1 | 1 | 8 |
| DLBCL | CD19 lyphodepletion flu/cy+beam | ≥3 grades thrombocytopenia | Wenjing Luo | 2022 | 1 | 0 | 1 | 0 | 1 | 1 | 1 | 0 | 1 | 1 | 1 | 8 |
| DLBCL | CD19 lyphodepletion flu/cy+beam | ≥3 grades anemia | Wenjing Luo | 2022 | 1 | 0 | 1 | 0 | 1 | 1 | 1 | 0 | 1 | 1 | 1 | 8 |
| DLBCL | CD19 lyphodepletion flu | Any grades Neutropenia | Wenjing Luo | 2022 | 1 | 0 | 1 | 0 | 1 | 1 | 1 | 0 | 1 | 1 | 1 | 8 |
| DLBCL | CD19 lyphodepletion flu | Any grades thrombocytopenia | Wenjing Luo | 2022 | 1 | 0 | 1 | 0 | 1 | 1 | 1 | 0 | 1 | 1 | 1 | 8 |
| DLBCL | CD19 lyphodepletion flu | Any grades anemia | Wenjing Luo | 2022 | 1 | 0 | 1 | 0 | 1 | 1 | 1 | 0 | 1 | 1 | 1 | 8 |
| DLBCL | CD19 lyphodepletion flu | ≥3 grades Neutropenia | Wenjing Luo | 2022 | 1 | 0 | 1 | 0 | 1 | 1 | 1 | 0 | 1 | 1 | 1 | 8 |
| DLBCL | CD19 lyphodepletion flu | ≥3 grades thrombocytopenia | Wenjing Luo | 2022 | 1 | 0 | 1 | 0 | 1 | 1 | 1 | 0 | 1 | 1 | 1 | 8 |
| DLBCL | CD19 lyphodepletion flu | ≥3 grades anemia | Wenjing Luo | 2022 | 1 | 0 | 1 | 0 | 1 | 1 | 1 | 0 | 1 | 1 | 1 | 8 |
| DLBCL | CD19 lyphodepletion beam | Any grades Neutropenia | Wenjing Luo | 2022 | 1 | 0 | 1 | 0 | 1 | 1 | 1 | 0 | 1 | 1 | 1 | 8 |
| DLBCL | CD19 lyphodepletion beam | Any grades thrombocytopenia | Wenjing Luo | 2022 | 1 | 0 | 1 | 0 | 1 | 1 | 1 | 0 | 1 | 1 | 1 | 8 |
| DLBCL | CD19 lyphodepletion beam | Any grades anemia | Wenjing Luo | 2022 | 1 | 0 | 1 | 0 | 1 | 1 | 1 | 0 | 1 | 1 | 1 | 8 |
| DLBCL | CD19 lyphodepletion beam | ≥3 grades Neutropenia | Wenjing Luo | 2022 | 1 | 0 | 1 | 0 | 1 | 1 | 1 | 0 | 1 | 1 | 1 | 8 |
| DLBCL | CD19 lyphodepletion beam | ≥3 grades thrombocytopenia | Wenjing Luo | 2022 | 1 | 0 | 1 | 0 | 1 | 1 | 1 | 0 | 1 | 1 | 1 | 8 |
| DLBCL | CD19 lyphodepletion beam | ≥3 grades anemia | Wenjing Luo | 2022 | 1 | 0 | 1 | 0 | 1 | 1 | 1 | 0 | 1 | 1 | 1 | 8 |
| MM | LCAR-B38M 41bb lentiviros | Any grades Neutropenia | Wenjing Luo | 2022 | 1 | 0 | 1 | 0 | 1 | 1 | 1 | 0 | 1 | 1 | 1 | 8 |
| MM | LCAR-B38M 41bb lentiviros | Any grades thrombocytopenia | Wenjing Luo | 2022 | 1 | 0 | 1 | 0 | 1 | 1 | 1 | 0 | 1 | 1 | 1 | 8 |
| MM | LCAR-B38M 41bb lentiviros | Any grades anemia | Wenjing Luo | 2022 | 1 | 0 | 1 | 0 | 1 | 1 | 1 | 0 | 1 | 1 | 1 | 8 |
| MM | LCAR-B38M 41bb lentiviros | ≥3 grades Neutropenia | Wenjing Luo | 2022 | 1 | 0 | 1 | 0 | 1 | 1 | 1 | 0 | 1 | 1 | 1 | 8 |
| MM | LCAR-B38M 41bb lentiviros | ≥3 grades thrombocytopenia | Wenjing Luo | 2022 | 1 | 0 | 1 | 0 | 1 | 1 | 1 | 0 | 1 | 1 | 1 | 8 |
| MM | LCAR-B38M 41bb lentiviros | ≥3 grades anemia | Wenjing Luo | 2022 | 1 | 0 | 1 | 0 | 1 | 1 | 1 | 0 | 1 | 1 | 1 | 8 |
| MM | BCMA 41bb lentiviros | Any grades Neutropenia | Wenjing Luo | 2022 | 1 | 0 | 1 | 0 | 1 | 1 | 1 | 0 | 1 | 1 | 1 | 8 |
| MM | BCMA 41bb lentiviros | Any grades thrombocytopenia | Wenjing Luo | 2022 | 1 | 0 | 1 | 0 | 1 | 1 | 1 | 0 | 1 | 1 | 1 | 8 |
| MM | BCMA 41bb lentiviros | Any grades anemia | Wenjing Luo | 2022 | 1 | 0 | 1 | 0 | 1 | 1 | 1 | 0 | 1 | 1 | 1 | 8 |
| MM | BCMA 41bb lentiviros | ≥3 grades Neutropenia | Wenjing Luo | 2022 | 1 | 0 | 1 | 0 | 1 | 1 | 1 | 0 | 1 | 1 | 1 | 8 |
| MM | BCMA 41bb lentiviros | ≥3 grades thrombocytopenia | Wenjing Luo | 2022 | 1 | 0 | 1 | 0 | 1 | 1 | 1 | 0 | 1 | 1 | 1 | 8 |
| MM | BCMA 41bb lentiviros | ≥3 grades anemia | Wenjing Luo | 2022 | 1 | 0 | 1 | 0 | 1 | 1 | 1 | 0 | 1 | 1 | 1 | 8 |
| MM | LCAR-B38M lymphodeletion flu/cy | Any grades Neutropenia | Wenjing Luo | 2022 | 1 | 0 | 1 | 0 | 1 | 1 | 1 | 0 | 1 | 1 | 1 | 8 |
| MM | LCAR-B38M lymphodeletion flu/cy | Any grades thrombocytopenia | Wenjing Luo | 2022 | 1 | 0 | 1 | 0 | 1 | 1 | 1 | 0 | 1 | 1 | 1 | 8 |
| MM | LCAR-B38M lymphodeletion flu/cy | Any grades anemia | Wenjing Luo | 2022 | 1 | 0 | 1 | 0 | 1 | 1 | 1 | 0 | 1 | 1 | 1 | 8 |
| MM | LCAR-B38M lymphodeletion flu/cy | ≥3 grades Neutropenia | Wenjing Luo | 2022 | 1 | 0 | 1 | 0 | 1 | 1 | 1 | 0 | 1 | 1 | 1 | 8 |
| MM | LCAR-B38M lymphodeletion flu/cy | ≥3 grades thrombocytopenia | Wenjing Luo | 2022 | 1 | 0 | 1 | 0 | 1 | 1 | 1 | 0 | 1 | 1 | 1 | 8 |
| MM | LCAR-B38M lymphodeletion flu/cy | ≥3 grades anemia | Wenjing Luo | 2022 | 1 | 0 | 1 | 0 | 1 | 1 | 1 | 0 | 1 | 1 | 1 | 8 |
| MM | LCAR-B38M lymphodeletion cy | Any grades Neutropenia | Wenjing Luo | 2022 | 1 | 0 | 1 | 0 | 1 | 1 | 1 | 0 | 1 | 1 | 1 | 8 |
| MM | LCAR-B38M lymphodeletion cy | Any grades thrombocytopenia | Wenjing Luo | 2022 | 1 | 0 | 1 | 0 | 1 | 1 | 1 | 0 | 1 | 1 | 1 | 8 |
| MM | LCAR-B38M lymphodeletion cy | Any grades anemia | Wenjing Luo | 2022 | 1 | 0 | 1 | 0 | 1 | 1 | 1 | 0 | 1 | 1 | 1 | 8 |
| MM | LCAR-B38M lymphodeletion cy | ≥3 grades Neutropenia | Wenjing Luo | 2022 | 1 | 0 | 1 | 0 | 1 | 1 | 1 | 0 | 1 | 1 | 1 | 8 |
| MM | LCAR-B38M lymphodeletion cy | ≥3 grades thrombocytopenia | Wenjing Luo | 2022 | 1 | 0 | 1 | 0 | 1 | 1 | 1 | 0 | 1 | 1 | 1 | 8 |
| MM | LCAR-B38M lymphodeletion cy | ≥3 grades anemia | Wenjing Luo | 2022 | 1 | 0 | 1 | 0 | 1 | 1 | 1 | 0 | 1 | 1 | 1 | 8 |
| MM | BCMA T infused down 10e7 | Any grades Neutropenia | Wenjing Luo | 2022 | 1 | 0 | 1 | 0 | 1 | 1 | 1 | 0 | 1 | 1 | 1 | 8 |
| MM | BCMA T infused down 10e7 | Any grades thrombocytopenia | Wenjing Luo | 2022 | 1 | 0 | 1 | 0 | 1 | 1 | 1 | 0 | 1 | 1 | 1 | 8 |
| MM | BCMA T infused down 10e7 | Any grades anemia | Wenjing Luo | 2022 | 1 | 0 | 1 | 0 | 1 | 1 | 1 | 0 | 1 | 1 | 1 | 8 |
| MM | BCMA T infused down 10e7 | ≥3 grades Neutropenia | Wenjing Luo | 2022 | 1 | 0 | 1 | 0 | 1 | 1 | 1 | 0 | 1 | 1 | 1 | 8 |
| MM | BCMA T infused down 10e7 | ≥3 grades thrombocytopenia | Wenjing Luo | 2022 | 1 | 0 | 1 | 0 | 1 | 1 | 1 | 0 | 1 | 1 | 1 | 8 |
| MM | BCMA T infused down 10e7 | ≥3 grades anemia | Wenjing Luo | 2022 | 1 | 0 | 1 | 0 | 1 | 1 | 1 | 0 | 1 | 1 | 1 | 8 |
| MM | BCMA T infused up 10e7 | Any grades Neutropenia | Wenjing Luo | 2022 | 1 | 0 | 1 | 0 | 1 | 1 | 1 | 0 | 1 | 1 | 1 | 8 |
| MM | BCMA T infused up 10e7 | Any grades thrombocytopenia | Wenjing Luo | 2022 | 1 | 0 | 1 | 0 | 1 | 1 | 1 | 0 | 1 | 1 | 1 | 8 |
| MM | BCMA T infused up 10e7 | Any grades anemia | Wenjing Luo | 2022 | 1 | 0 | 1 | 0 | 1 | 1 | 1 | 0 | 1 | 1 | 1 | 8 |
| MM | BCMA T infused up 10e7 | ≥3 grades Neutropenia | Wenjing Luo | 2022 | 1 | 0 | 1 | 0 | 1 | 1 | 1 | 0 | 1 | 1 | 1 | 8 |
| MM | BCMA T infused up 10e7 | ≥3 grades thrombocytopenia | Wenjing Luo | 2022 | 1 | 0 | 1 | 0 | 1 | 1 | 1 | 0 | 1 | 1 | 1 | 8 |
| MM | BCMA T infused up 10e7 | ≥3 grades anemia | Wenjing Luo | 2022 | 1 | 0 | 1 | 0 | 1 | 1 | 1 | 0 | 1 | 1 | 1 | 8 |
| MM | BCMA +CD19 lymphodeletion BENDAM | Any grades Neutropenia | Wenjing Luo | 2022 | 1 | 0 | 1 | 0 | 1 | 1 | 1 | 0 | 1 | 1 | 1 | 8 |
| MM | BCMA +CD19 lymphodeletion BENDAM | Any grades thrombocytopenia | Wenjing Luo | 2022 | 1 | 0 | 1 | 0 | 1 | 1 | 1 | 0 | 1 | 1 | 1 | 8 |
| MM | BCMA +CD19 lymphodeletion BENDAM | Any grades anemia | Wenjing Luo | 2022 | 1 | 0 | 1 | 0 | 1 | 1 | 1 | 0 | 1 | 1 | 1 | 8 |
| MM | BCMA +CD19 lymphodeletion BENDAM | ≥3 grades Neutropenia | Wenjing Luo | 2022 | 1 | 0 | 1 | 0 | 1 | 1 | 1 | 0 | 1 | 1 | 1 | 8 |
| MM | BCMA +CD19 lymphodeletion BENDAM | ≥3 grades thrombocytopenia | Wenjing Luo | 2022 | 1 | 0 | 1 | 0 | 1 | 1 | 1 | 0 | 1 | 1 | 1 | 8 |
| MM | BCMA +CD19 lymphodeletion BENDAM | ≥3 grades anemia | Wenjing Luo | 2022 | 1 | 0 | 1 | 0 | 1 | 1 | 1 | 0 | 1 | 1 | 1 | 8 |
| MM | BCMA +CD19 lymphodeletion cy | Any grades Neutropenia | Wenjing Luo | 2022 | 1 | 0 | 1 | 0 | 1 | 1 | 1 | 0 | 1 | 1 | 1 | 8 |
| MM | BCMA +CD19 lymphodeletion cy | Any grades thrombocytopenia | Wenjing Luo | 2022 | 1 | 0 | 1 | 0 | 1 | 1 | 1 | 0 | 1 | 1 | 1 | 8 |
| MM | BCMA +CD19 lymphodeletion cy | Any grades anemia | Wenjing Luo | 2022 | 1 | 0 | 1 | 0 | 1 | 1 | 1 | 0 | 1 | 1 | 1 | 8 |
| MM | BCMA +CD19 lymphodeletion cy | ≥3 grades Neutropenia | Wenjing Luo | 2022 | 1 | 0 | 1 | 0 | 1 | 1 | 1 | 0 | 1 | 1 | 1 | 8 |
| MM | BCMA +CD19 lymphodeletion cy | ≥3 grades thrombocytopenia | Wenjing Luo | 2022 | 1 | 0 | 1 | 0 | 1 | 1 | 1 | 0 | 1 | 1 | 1 | 8 |
| MM | BCMA +CD19 lymphodeletion cy | ≥3 grades anemia | Wenjing Luo | 2022 | 1 | 0 | 1 | 0 | 1 | 1 | 1 | 0 | 1 | 1 | 1 | 8 |
| MM | BCMA +CD19 lymphodeletion ful/cy | Any grades Neutropenia | Wenjing Luo | 2022 | 1 | 0 | 1 | 0 | 1 | 1 | 1 | 0 | 1 | 1 | 1 | 8 |
| MM | BCMA +CD19 lymphodeletion ful/cy | Any grades thrombocytopenia | Wenjing Luo | 2022 | 1 | 0 | 1 | 0 | 1 | 1 | 1 | 0 | 1 | 1 | 1 | 8 |
| MM | BCMA +CD19 lymphodeletion ful/cy | Any grades anemia | Wenjing Luo | 2022 | 1 | 0 | 1 | 0 | 1 | 1 | 1 | 0 | 1 | 1 | 1 | 8 |
| MM | BCMA +CD19 lymphodeletion ful/cy | ≥3 grades Neutropenia | Wenjing Luo | 2022 | 1 | 0 | 1 | 0 | 1 | 1 | 1 | 0 | 1 | 1 | 1 | 8 |
| MM | BCMA +CD19 lymphodeletion ful/cy | ≥3 grades thrombocytopenia | Wenjing Luo | 2022 | 1 | 0 | 1 | 0 | 1 | 1 | 1 | 0 | 1 | 1 | 1 | 8 |
| MM | BCMA +CD19 lymphodeletion ful/cy | ≥3 grades anemia | Wenjing Luo | 2022 | 1 | 0 | 1 | 0 | 1 | 1 | 1 | 0 | 1 | 1 | 1 | 8 |
| RRLBCL | CD19 28Z | BOR | Zhitao Ying | 2022 | 0 | 1 | 1 | 0 | 1 | 1 | 1 | 0 | 1 | 1 | 1 | 8 |
| RRLBCL | CD19 28Z | BCR | Zhitao Ying | 2022 | 0 | 1 | 1 | 0 | 1 | 1 | 1 | 0 | 1 | 1 | 1 | 8 |
| RRLBCL | CD19 28Z | 3 month-CR | Zhitao Ying | 2022 | 0 | 1 | 1 | 0 | 1 | 1 | 1 | 0 | 1 | 1 | 1 | 8 |
| RRLBCL | CD19 28Z | 12 month -OS | Zhitao Ying | 2022 | 0 | 1 | 1 | 0 | 1 | 1 | 1 | 0 | 1 | 1 | 1 | 8 |
| RRLBCL | CD19 28Z | DOR | Zhitao Ying | 2022 | 0 | 1 | 1 | 0 | 1 | 1 | 1 | 0 | 1 | 1 | 1 | 8 |
| RRLBCL | CD19 28Z | Any grades CRS | Zhitao Ying | 2022 | 0 | 1 | 1 | 0 | 1 | 1 | 1 | 0 | 1 | 1 | 1 | 8 |
| RRLBCL | CD19 28Z | ≥3 grades CRS | Zhitao Ying | 2022 | 0 | 1 | 1 | 0 | 1 | 1 | 1 | 0 | 1 | 1 | 1 | 8 |
| RRLBCL | CD19 28Z | Any grades Neurotoxicity | Zhitao Ying | 2022 | 0 | 1 | 1 | 0 | 1 | 1 | 1 | 0 | 1 | 1 | 1 | 8 |
| RRLBCL | CD19 28Z | ≥3 grades Neurotoxicity | Zhitao Ying | 2022 | 0 | 1 | 1 | 0 | 1 | 1 | 1 | 0 | 1 | 1 | 1 | 8 |
| DLBCL | CD19 28Z/retrovirus | ORR | Na Wang | 2021 | 0 | 1 | 1 | 0 | 1 | 1 | 0 | 0 | 1 | 0 | 1 | 6 |
| CLL | k light chain lymphodepletion cy | ORR | Na Wang | 2021 | 0 | 1 | 1 | 0 | 1 | 1 | 0 | 0 | 1 | 0 | 1 | 6 |
| CLL | k light chain no lymphodepletion | ORR | Na Wang | 2021 | 0 | 1 | 1 | 0 | 1 | 1 | 0 | 0 | 1 | 0 | 1 | 6 |
| CLL | CD19 28Z retrovirus | ORR | Na Wang | 2021 | 0 | 1 | 1 | 0 | 1 | 1 | 0 | 0 | 1 | 0 | 1 | 6 |
| CLL | k light chain 28Z retrovirus | ORR | Na Wang | 2021 | 0 | 1 | 1 | 0 | 1 | 1 | 0 | 0 | 1 | 0 | 1 | 6 |
| DLBCL | CD19 lyphodepletion  Flu/cy IL2 | ORR | Na Wang | 2021 | 0 | 1 | 1 | 0 | 1 | 1 | 0 | 0 | 1 | 0 | 1 | 6 |
| DLBCL | CD20BBZ  +IL2 | ORR | Na Wang | 2021 | 0 | 1 | 1 | 0 | 1 | 1 | 0 | 0 | 1 | 0 | 1 | 6 |
| 19  B-NHL | CD19 41BB | Relapsed rate CAR-T infusion 12 month | Alessia Zinzi | 2023 | 0 | 1 | 1 | 0 | 1 | 1 | 0 | 0 | 1 | 0 | 1 | 6 |
| B-NHL | CD19 41BB | Relapsed rate CAR-T infusion over 1 year | Alessia Zinzi | 2023 | 0 | 1 | 1 | 0 | 1 | 1 | 0 | 0 | 1 | 0 | 1 | 6 |
| B-NHL | CD19+CD22 41BB/lentivirus murine | All grades anemia | Yuan Xia | 2022 | 1 | 1 | 1 | 0 | 1 | 1 | 1 | 0 | 1 | 1 | 1 | 9 |
| B-NHL | CD19+CD22 41BB/lentivirus murine | ≥3 grades anemia | Yuan Xia | 2022 | 1 | 1 | 1 | 0 | 1 | 1 | 1 | 0 | 1 | 1 | 1 | 9 |
| B-NHL | CD19+CD22 41BB/lentivirus murine | ≥3 thrombocytopenia | Yuan Xia | 2022 | 1 | 1 | 1 | 0 | 1 | 1 | 1 | 0 | 1 | 1 | 1 | 9 |
| B-NHL | CD19+CD22 41BB/lentivirus murine | ≥3 neutropenia | Yuan Xia | 2022 | 1 | 1 | 1 | 0 | 1 | 1 | 1 | 0 | 1 | 1 | 1 | 9 |
| B-NHL | CD19+CD22 41BB/lentivirus murine | ≥3 leukopenia | Yuan Xia | 2022 | 1 | 1 | 1 | 0 | 1 | 1 | 1 | 0 | 1 | 1 | 1 | 9 |
| B-NHL | CD19+CD22 41BB/lentivirus murine | ≥3 lymphocytopenia | Yuan Xia | 2022 | 1 | 1 | 1 | 0 | 1 | 1 | 1 | 0 | 1 | 1 | 1 | 9 |
| B-NHL | CD19+CD22 41BB/lentivirus murine | ≥3 febrile neutropenia | Yuan Xia | 2022 | 1 | 1 | 1 | 0 | 1 | 1 | 1 | 0 | 1 | 1 | 1 | 9 |
| B-NHL | CD19+CD22 41BB/lentivirus murine | All grades thrombocytopenia | Yuan Xia | 2022 | 1 | 1 | 1 | 0 | 1 | 1 | 1 | 0 | 1 | 1 | 1 | 9 |
| B-NHL | CD19+CD22 41BB/lentivirus murine | All grades neutropenia | Yuan Xia | 2022 | 1 | 1 | 1 | 0 | 1 | 1 | 1 | 0 | 1 | 1 | 1 | 9 |
| B-NHL | CD19+CD22 41BB/lentivirus murine | All grades leukopenia | Yuan Xia | 2022 | 1 | 1 | 1 | 0 | 1 | 1 | 1 | 0 | 1 | 1 | 1 | 9 |
| B-NHL | CD19+CD22 41BB/lentivirus murine | All grades lymphocytopenia | Yuan Xia | 2022 | 1 | 1 | 1 | 0 | 1 | 1 | 1 | 0 | 1 | 1 | 1 | 9 |
| B-NHL | CD19+CD22 41BB/lentivirus murine | All grades febrile neutropenia | Yuan Xia | 2022 | 1 | 1 | 1 | 0 | 1 | 1 | 1 | 0 | 1 | 1 | 1 | 9 |
| B-NHL | CD19+CD20 41BB/lentivirus murine | All grades anemia | Yuan Xia | 2022 | 1 | 1 | 1 | 0 | 1 | 1 | 1 | 0 | 1 | 1 | 1 | 9 |
| B-NHL | CD19+CD20 41BB/lentivirus murine | ≥3 grades anemia | Yuan Xia | 2022 | 1 | 1 | 1 | 0 | 1 | 1 | 1 | 0 | 1 | 1 | 1 | 9 |
| B-NHL | CD19+CD20 41BB/lentivirus murine | ≥3 thrombocytopenia | Yuan Xia | 2022 | 1 | 1 | 1 | 0 | 1 | 1 | 1 | 0 | 1 | 1 | 1 | 9 |
| B-NHL | CD19+CD20 41BB/lentivirus murine | ≥3 neutropenia | Yuan Xia | 2022 | 1 | 1 | 1 | 0 | 1 | 1 | 1 | 0 | 1 | 1 | 1 | 9 |
| B-NHL | CD19+CD20 41BB/lentivirus murine | ≥3 leukopenia | Yuan Xia | 2022 | 1 | 1 | 1 | 0 | 1 | 1 | 1 | 0 | 1 | 1 | 1 | 9 |
| B-NHL | CD19+CD20 41BB/lentivirus murine | ≥3 lymphocytopenia | Yuan Xia | 2022 | 1 | 1 | 1 | 0 | 1 | 1 | 1 | 0 | 1 | 1 | 1 | 9 |
| B-NHL | CD19+CD20 41BB/lentivirus murine | ≥3 febrile neutropenia | Yuan Xia | 2022 | 1 | 1 | 1 | 0 | 1 | 1 | 1 | 0 | 1 | 1 | 1 | 9 |
| B-NHL | CD19+CD20 41BB/lentivirus murine | All grades thrombocytopenia | Yuan Xia | 2022 | 1 | 1 | 1 | 0 | 1 | 1 | 1 | 0 | 1 | 1 | 1 | 9 |
| B-NHL | CD19+CD20 41BB/lentivirus murine | All grades neutropenia | Yuan Xia | 2022 | 1 | 1 | 1 | 0 | 1 | 1 | 1 | 0 | 1 | 1 | 1 | 9 |
| B-NHL | CD19+CD20 41BB/lentivirus murine | All grades leukopenia | Yuan Xia | 2022 | 1 | 1 | 1 | 0 | 1 | 1 | 1 | 0 | 1 | 1 | 1 | 9 |
| B-NHL | CD19+CD20 41BB/lentivirus murine | All grades lymphocytopenia | Yuan Xia | 2022 | 1 | 1 | 1 | 0 | 1 | 1 | 1 | 0 | 1 | 1 | 1 | 9 |
| B-NHL | CD19+CD20 41BB/lentivirus murine | All grades febrile neutropenia | Yuan Xia | 2022 | 1 | 1 | 1 | 0 | 1 | 1 | 1 | 0 | 1 | 1 | 1 | 9 |
| B-NHL | CD19 41BB+DAP12 | All grades anemia | Yuan Xia | 2022 | 1 | 1 | 1 | 0 | 1 | 1 | 1 | 0 | 1 | 1 | 1 | 9 |
| B-NHL | CD19 41BB+DAP12 | ≥3 grades anemia | Yuan Xia | 2022 | 1 | 1 | 1 | 0 | 1 | 1 | 1 | 0 | 1 | 1 | 1 | 9 |
| B-NHL | CD19 41BB+DAP12 | ≥3 thrombocytopenia | Yuan Xia | 2022 | 1 | 1 | 1 | 0 | 1 | 1 | 1 | 0 | 1 | 1 | 1 | 9 |
| B-NHL | CD19 41BB+DAP12 | ≥3 neutropenia | Yuan Xia | 2022 | 1 | 1 | 1 | 0 | 1 | 1 | 1 | 0 | 1 | 1 | 1 | 9 |
| B-NHL | CD19 41BB+DAP12 | ≥3 leukopenia | Yuan Xia | 2022 | 1 | 1 | 1 | 0 | 1 | 1 | 1 | 0 | 1 | 1 | 1 | 9 |
| B-NHL | CD19 41BB+DAP12 | ≥3 lymphocytopenia | Yuan Xia | 2022 | 1 | 1 | 1 | 0 | 1 | 1 | 1 | 0 | 1 | 1 | 1 | 9 |
| B-NHL | CD19 41BB+DAP12 | ≥3 febrile neutropenia | Yuan Xia | 2022 | 1 | 1 | 1 | 0 | 1 | 1 | 1 | 0 | 1 | 1 | 1 | 9 |
| B-NHL | CD19 41BB+DAP12 | All grades thrombocytopenia | Yuan Xia | 2022 | 1 | 1 | 1 | 0 | 1 | 1 | 1 | 0 | 1 | 1 | 1 | 9 |
| B-NHL | CD19 41BB+DAP12 | All grades neutropenia | Yuan Xia | 2022 | 1 | 1 | 1 | 0 | 1 | 1 | 1 | 0 | 1 | 1 | 1 | 9 |
| B-NHL | CD19 41BB+DAP12 | All grades leukopenia | Yuan Xia | 2022 | 1 | 1 | 1 | 0 | 1 | 1 | 1 | 0 | 1 | 1 | 1 | 9 |
| B-NHL | CD19 41BB+DAP12 | All grades lymphocytopenia | Yuan Xia | 2022 | 1 | 1 | 1 | 0 | 1 | 1 | 1 | 0 | 1 | 1 | 1 | 9 |
| B-NHL | CD19 41BB+DAP12 | All grades febrile neutropenia | Yuan Xia | 2022 | 1 | 1 | 1 | 0 | 1 | 1 | 1 | 0 | 1 | 1 | 1 | 9 |
| B-ALL | CD19 Lentiviral/41BB murine | All grades anemia | Yuan Xia | 2022 | 1 | 1 | 1 | 0 | 1 | 1 | 1 | 0 | 1 | 1 | 1 | 9 |
| B-ALL | CD19 Lentiviral/41BB murine | ≥3 grades anemia | Yuan Xia | 2022 | 1 | 1 | 1 | 0 | 1 | 1 | 1 | 0 | 1 | 1 | 1 | 9 |
| B-ALL | CD19 Lentiviral/41BB murine | ≥3 thrombocytopenia | Yuan Xia | 2022 | 1 | 1 | 1 | 0 | 1 | 1 | 1 | 0 | 1 | 1 | 1 | 9 |
| B-ALL | CD19 Lentiviral/41BB murine | ≥3 neutropenia | Yuan Xia | 2022 | 1 | 1 | 1 | 0 | 1 | 1 | 1 | 0 | 1 | 1 | 1 | 9 |
| B-ALL | CD19 Lentiviral/41BB murine | ≥3 leukopenia | Yuan Xia | 2022 | 1 | 1 | 1 | 0 | 1 | 1 | 1 | 0 | 1 | 1 | 1 | 9 |
| B-ALL | CD19 Lentiviral/41BB murine | ≥3 lymphocytopenia | Yuan Xia | 2022 | 1 | 1 | 1 | 0 | 1 | 1 | 1 | 0 | 1 | 1 | 1 | 9 |
| B-ALL | CD19 Lentiviral/41BB murine | ≥3 febrile neutropenia | Yuan Xia | 2022 | 1 | 1 | 1 | 0 | 1 | 1 | 1 | 0 | 1 | 1 | 1 | 9 |
| B-ALL | CD19 Lentiviral/41BB murine | All grades thrombocytopenia | Yuan Xia | 2022 | 1 | 1 | 1 | 0 | 1 | 1 | 1 | 0 | 1 | 1 | 1 | 9 |
| B-ALL | CD19 Lentiviral/41BB murine | All grades neutropenia | Yuan Xia | 2022 | 1 | 1 | 1 | 0 | 1 | 1 | 1 | 0 | 1 | 1 | 1 | 9 |
| B-ALL | CD19 Lentiviral/41BB murine | All grades leukopenia | Yuan Xia | 2022 | 1 | 1 | 1 | 0 | 1 | 1 | 1 | 0 | 1 | 1 | 1 | 9 |
| B-ALL | CD19 Lentiviral/41BB murine | All grades lymphocytopenia | Yuan Xia | 2022 | 1 | 1 | 1 | 0 | 1 | 1 | 1 | 0 | 1 | 1 | 1 | 9 |
| B-ALL | CD19 Lentiviral/41BB murine | All grades febrile neutropenia | Yuan Xia | 2022 | 1 | 1 | 1 | 0 | 1 | 1 | 1 | 0 | 1 | 1 | 1 | 9 |
| B-ALL | CD19 Lentiviral/41BB human | All grades anemia | Yuan Xia | 2022 | 1 | 1 | 1 | 0 | 1 | 1 | 1 | 0 | 1 | 1 | 1 | 9 |
| B-ALL | CD19 Lentiviral/41BB human | ≥3 grades anemia | Yuan Xia | 2022 | 1 | 1 | 1 | 0 | 1 | 1 | 1 | 0 | 1 | 1 | 1 | 9 |
| B-ALL | CD19 Lentiviral/41BB human | ≥3 thrombocytopenia | Yuan Xia | 2022 | 1 | 1 | 1 | 0 | 1 | 1 | 1 | 0 | 1 | 1 | 1 | 9 |
| B-ALL | CD19 Lentiviral/41BB human | ≥3 neutropenia | Yuan Xia | 2022 | 1 | 1 | 1 | 0 | 1 | 1 | 1 | 0 | 1 | 1 | 1 | 9 |
| B-ALL | CD19 Lentiviral/41BB human | ≥3 leukopenia | Yuan Xia | 2022 | 1 | 1 | 1 | 0 | 1 | 1 | 1 | 0 | 1 | 1 | 1 | 9 |
| B-ALL | CD19 Lentiviral/41BB human | ≥3 lymphocytopenia | Yuan Xia | 2022 | 1 | 1 | 1 | 0 | 1 | 1 | 1 | 0 | 1 | 1 | 1 | 9 |
| B-ALL | CD19 Lentiviral/41BB human | ≥3 febrile neutropenia | Yuan Xia | 2022 | 1 | 1 | 1 | 0 | 1 | 1 | 1 | 0 | 1 | 1 | 1 | 9 |
| B-ALL | CD19 Lentiviral/41BB human | All grades thrombocytopenia | Yuan Xia | 2022 | 1 | 1 | 1 | 0 | 1 | 1 | 1 | 0 | 1 | 1 | 1 | 9 |
| B-ALL | CD19 Lentiviral/41BB human | All grades neutropenia | Yuan Xia | 2022 | 1 | 1 | 1 | 0 | 1 | 1 | 1 | 0 | 1 | 1 | 1 | 9 |
| B-ALL | CD19 Lentiviral/41BB human | All grades leukopenia | Yuan Xia | 2022 | 1 | 1 | 1 | 0 | 1 | 1 | 1 | 0 | 1 | 1 | 1 | 9 |
| B-ALL | CD19 Lentiviral/41BB human | All grades lymphocytopenia | Yuan Xia | 2022 | 1 | 1 | 1 | 0 | 1 | 1 | 1 | 0 | 1 | 1 | 1 | 9 |
| B-ALL | CD19 Lentiviral/41BB human | All grades febrile neutropenia | Yuan Xia | 2022 | 1 | 1 | 1 | 0 | 1 | 1 | 1 | 0 | 1 | 1 | 1 | 9 |
| B-ALL | CD19 lyphodepletion flu/cy+Alem-tuzumab | thrombo- cytopenia, neutropenia, leukaemia | Yuan Xia | 2022 | 1 | 1 | 1 | 0 | 1 | 1 | 1 | 0 | 1 | 1 | 1 | 9 |
| B-ALL | CD19 lyphodepletion flu/cy+cytarabine | All grades anemia | Yuan Xia | 2022 | 1 | 1 | 1 | 0 | 1 | 1 | 1 | 0 | 1 | 1 | 1 | 9 |
| B-ALL | CD19 lyphodepletion flu/cy+cytarabine | ≥3 grades anemia | Yuan Xia | 2022 | 1 | 1 | 1 | 0 | 1 | 1 | 1 | 0 | 1 | 1 | 1 | 9 |
| B-ALL | CD19 lyphodepletion flu/cy+cytarabine | ≥3 thrombocytopenia | Yuan Xia | 2022 | 1 | 1 | 1 | 0 | 1 | 1 | 1 | 0 | 1 | 1 | 1 | 9 |
| B-ALL | CD19 lyphodepletion flu/cy+cytarabine | ≥3 neutropenia | Yuan Xia | 2022 | 1 | 1 | 1 | 0 | 1 | 1 | 1 | 0 | 1 | 1 | 1 | 9 |
| B-ALL | CD19 lyphodepletion flu/cy+cytarabine | ≥3 leukopenia | Yuan Xia | 2022 | 1 | 1 | 1 | 0 | 1 | 1 | 1 | 0 | 1 | 1 | 1 | 9 |
| B-ALL | CD19 lyphodepletion flu/cy+cytarabine | ≥3 lymphocytopenia | Yuan Xia | 2022 | 1 | 1 | 1 | 0 | 1 | 1 | 1 | 0 | 1 | 1 | 1 | 9 |
| B-ALL | CD19 lyphodepletion flu/cy+cytarabine | ≥3 febrile neutropenia | Yuan Xia | 2022 | 1 | 1 | 1 | 0 | 1 | 1 | 1 | 0 | 1 | 1 | 1 | 9 |
| B-ALL | CD19 lyphodepletion flu/cy+cytarabine | All grades thrombocytopenia | Yuan Xia | 2022 | 1 | 1 | 1 | 0 | 1 | 1 | 1 | 0 | 1 | 1 | 1 | 9 |
| B-ALL | CD19 lyphodepletion flu/cy+cytarabine | All grades neutropenia | Yuan Xia | 2022 | 1 | 1 | 1 | 0 | 1 | 1 | 1 | 0 | 1 | 1 | 1 | 9 |
| B-ALL | CD19 lyphodepletion flu/cy+cytarabine | All grades leukopenia | Yuan Xia | 2022 | 1 | 1 | 1 | 0 | 1 | 1 | 1 | 0 | 1 | 1 | 1 | 9 |
| B-ALL | CD19 lyphodepletion flu/cy+cytarabine | All grades lymphocytopenia | Yuan Xia | 2022 | 1 | 1 | 1 | 0 | 1 | 1 | 1 | 0 | 1 | 1 | 1 | 9 |
| B-ALL | CD19 lyphodepletion flu/cy+cytarabine | All grades febrile neutropenia | Yuan Xia | 2022 | 1 | 1 | 1 | 0 | 1 | 1 | 1 | 0 | 1 | 1 | 1 | 9 |
| B-ALL | CD19 lyphodepletion flu/cy+Alem-tuzumab | All grades anemia | Yuan Xia | 2022 | 1 | 1 | 1 | 0 | 1 | 1 | 1 | 0 | 1 | 1 | 1 | 9 |
| B-ALL | CD19 lyphodepletion flu/cy+Alem-tuzumab | ≥3 grades anemia | Yuan Xia | 2022 | 1 | 1 | 1 | 0 | 1 | 1 | 1 | 0 | 1 | 1 | 1 | 9 |
| B-ALL | CD19 lyphodepletion flu/cy+Alem-tuzumab | ≥3 thrombocytopenia | Yuan Xia | 2022 | 1 | 1 | 1 | 0 | 1 | 1 | 1 | 0 | 1 | 1 | 1 | 9 |
| B-ALL | CD19 lyphodepletion flu/cy+Alem-tuzumab | ≥3 neutropenia | Yuan Xia | 2022 | 1 | 1 | 1 | 0 | 1 | 1 | 1 | 0 | 1 | 1 | 1 | 9 |
| B-ALL | CD19 lyphodepletion flu/cy+Alem-tuzumab | ≥3 leukopenia | Yuan Xia | 2022 | 1 | 1 | 1 | 0 | 1 | 1 | 1 | 0 | 1 | 1 | 1 | 9 |
| B-ALL | CD19 lyphodepletion flu/cy+Alem-tuzumab | ≥3 lymphocytopenia | Yuan Xia | 2022 | 1 | 1 | 1 | 0 | 1 | 1 | 1 | 0 | 1 | 1 | 1 | 9 |
| B-ALL | CD19 lyphodepletion flu/cy+Alem-tuzumab | ≥3 febrile neutropenia | Yuan Xia | 2022 | 1 | 1 | 1 | 0 | 1 | 1 | 1 | 0 | 1 | 1 | 1 | 9 |
| B-ALL | CD19 lyphodepletion flu/cy+Alem-tuzumab | All grades thrombocytopenia | Yuan Xia | 2022 | 1 | 1 | 1 | 0 | 1 | 1 | 1 | 0 | 1 | 1 | 1 | 9 |
| B-ALL | CD19 lyphodepletion flu/cy+Alem-tuzumab | All grades neutropenia | Yuan Xia | 2022 | 1 | 1 | 1 | 0 | 1 | 1 | 1 | 0 | 1 | 1 | 1 | 9 |
| B-ALL | CD19 lyphodepletion flu/cy+Alem-tuzumab | All grades leukopenia | Yuan Xia | 2022 | 1 | 1 | 1 | 0 | 1 | 1 | 1 | 0 | 1 | 1 | 1 | 9 |
| B-ALL | CD19 lyphodepletion flu/cy+Alem-tuzumab | All grades lymphocytopenia | Yuan Xia | 2022 | 1 | 1 | 1 | 0 | 1 | 1 | 1 | 0 | 1 | 1 | 1 | 9 |
| B-ALL | CD19 lyphodepletion flu/cy+Alem-tuzumab | All grades febrile neutropenia | Yuan Xia | 2022 | 1 | 1 | 1 | 0 | 1 | 1 | 1 | 0 | 1 | 1 | 1 | 9 |
| B-NHL | CD19+CD20 lyphodepletion flu/cy | All grades lymphocytopenia | Yuan Xia | 2022 | 1 | 1 | 1 | 0 | 1 | 1 | 1 | 0 | 1 | 1 | 1 | 9 |
| B-NHL | CD19+CD20 lyphodepletion flu/cy+ifosfamide | All grades febrile neutropenia | Yuan Xia | 2022 | 1 | 1 | 1 | 0 | 1 | 1 | 1 | 0 | 1 | 1 | 1 | 9 |
| B-NHL | CD19-28 lentivirus  virus murine | All grades anemia | Yuan Xia | 2022 | 1 | 1 | 1 | 0 | 1 | 1 | 1 | 0 | 1 | 1 | 1 | 9 |
| B-NHL | CD19-28  Gammaretrovirus murine | ≥3 grades anemia | Yuan Xia | 2022 | 1 | 1 | 1 | 0 | 1 | 1 | 1 | 0 | 1 | 1 | 1 | 9 |
| B-NHL | CD19 28Z murine | ≥3 thrombocytopenia | Yuan Xia | 2022 | 1 | 1 | 1 | 0 | 1 | 1 | 1 | 0 | 1 | 1 | 1 | 9 |
| B-NHL | CD19 28z+41BBz murine | ≥3 neutropenia | Yuan Xia | 2022 | 1 | 1 | 1 | 0 | 1 | 1 | 1 | 0 | 1 | 1 | 1 | 9 |
| CLL | CD19 28Z murine | ≥3 leukopenia | Yuan Xia | 2022 | 1 | 1 | 1 | 0 | 1 | 1 | 1 | 0 | 1 | 1 | 1 | 9 |
| CLL | CD19 28z+41BBz murine | ≥3 lymphocytopenia | Yuan Xia | 2022 | 1 | 1 | 1 | 0 | 1 | 1 | 1 | 0 | 1 | 1 | 1 | 9 |
| CLL | CD19 28Z retrovirus murine | ≥3 febrile neutropenia | Yuan Xia | 2022 | 1 | 1 | 1 | 0 | 1 | 1 | 1 | 0 | 1 | 1 | 1 | 9 |
| CLL | CD19 41BB lenti murine | All grades lymphocytopenia | Yuan Xia | 2022 | 1 | 1 | 1 | 0 | 1 | 1 | 1 | 0 | 1 | 1 | 1 | 9 |
| CLL | CD19+CD20 41BBz lentirvirual murine | All grades febrile neutropenia | Yuan Xia | 2022 | 1 | 1 | 1 | 0 | 1 | 1 | 1 | 0 | 1 | 1 | 1 | 9 |
| CLL | CD19 41BBz lentirvirual murine | All grades anemia | Yuan Xia | 2022 | 1 | 1 | 1 | 0 | 1 | 1 | 1 | 0 | 1 | 1 | 1 | 9 |
| CLL | CD19 CD28/41BB Lenti murine | ≥3 grades anemia | Yuan Xia | 2022 | 1 | 1 | 1 | 0 | 1 | 1 | 1 | 0 | 1 | 1 | 1 | 9 |
| CLL | CD19 41BB Lenti murine | ≥3 thrombocytopenia | Yuan Xia | 2022 | 1 | 1 | 1 | 0 | 1 | 1 | 1 | 0 | 1 | 1 | 1 | 9 |
| DLBCL | CD19 CD28  lenti human | ≥3 neutropenia | Yuan Xia | 2022 | 1 | 1 | 1 | 0 | 1 | 1 | 1 | 0 | 1 | 1 | 1 | 9 |
| DLBCL | CD19 41BB lenti human | ≥3 leukopenia | Yuan Xia | 2022 | 1 | 1 | 1 | 0 | 1 | 1 | 1 | 0 | 1 | 1 | 1 | 9 |
| DLBCL | CD19 CD28 lenti human | ≥3 lymphocytopenia | Yuan Xia | 2022 | 1 | 1 | 1 | 0 | 1 | 1 | 1 | 0 | 1 | 1 | 1 | 9 |
| DLBCL | CD19 CD28  lenti murine | ≥3 febrile neutropenia | Yuan Xia | 2022 | 1 | 1 | 1 | 0 | 1 | 1 | 1 | 0 | 1 | 1 | 1 | 9 |
| DLBCL | CD19 41BB  Lenti human | All grades lymphocytopenia | Yuan Xia | 2022 | 1 | 1 | 1 | 0 | 1 | 1 | 1 | 0 | 1 | 1 | 1 | 9 |
| DLBCL | CD19 41BB  lenti murine | All grades febrile neutropenia | Yuan Xia | 2022 | 1 | 1 | 1 | 0 | 1 | 1 | 1 | 0 | 1 | 1 | 1 | 9 |
| CLL | CD19 41BB  Lenti human | All grades anemia | Yuan Xia | 2022 | 1 | 1 | 1 | 0 | 1 | 1 | 1 | 0 | 1 | 1 | 1 | 9 |
| CLL | CD19 41BB  lenti murine | ≥3 grades anemia | Yuan Xia | 2022 | 1 | 1 | 1 | 0 | 1 | 1 | 1 | 0 | 1 | 1 | 1 | 9 |
| ALL | CD19 CD28+41BB  retrovirus murine | ≥3 thrombocytopenia | Yuan Xia | 2022 | 1 | 1 | 1 | 0 | 1 | 1 | 1 | 0 | 1 | 1 | 1 | 9 |
| ALL | CD19 CD28 retrovirus murine | ≥3 neutropenia | Yuan Xia | 2022 | 1 | 1 | 1 | 0 | 1 | 1 | 1 | 0 | 1 | 1 | 1 | 9 |
| CNSL | CD19 BBz | Relapse rates | Jing Zhou | 2024 | 1 | 1 | 1 | 0 | 1 | 1 | 1 | 0 | 1 | 1 | 1 | 9 |
| CNSL | CD20 BBz | Relapse rates | Jing Zhou | 2024 | 1 | 1 | 1 | 0 | 1 | 1 | 1 | 0 | 1 | 1 | 1 | 9 |
| CNSL | CD22 BBz | Relapse rates | Jing Zhou | 2024 | 1 | 1 | 1 | 0 | 1 | 1 | 1 | 0 | 1 | 1 | 1 | 9 |
| CNSL | CD19+20+22 BBz | Relapse rates | Jing Zhou | 2024 | 1 | 1 | 1 | 0 | 1 | 1 | 1 | 0 | 1 | 1 | 1 | 9 |
| pCNSL | CD19 28+BBz | Relapse rates | Jing Zhou | 2024 | 1 | 1 | 1 | 0 | 1 | 1 | 1 | 0 | 1 | 1 | 1 | 9 |
| sCNSL | CD19 28+BBz | Relapse rates | Jing Zhou | 2024 | 1 | 1 | 1 | 0 | 1 | 1 | 1 | 0 | 1 | 1 | 1 | 9 |
| sCNSL | CD19  65y up | Relapse rates | Jing Zhou | 2024 | 1 | 1 | 1 | 0 | 1 | 1 | 1 | 0 | 1 | 1 | 1 | 9 |
| sCNSL | CD19  65y down | Relapse rates | Jing Zhou | 2024 | 1 | 1 | 1 | 0 | 1 | 1 | 1 | 0 | 1 | 1 | 1 | 9 |
| CNSL | CD19 +noHSCT | Relapse rates | Jing Zhou | 2024 | 1 | 1 | 1 | 0 | 1 | 1 | 1 | 0 | 1 | 1 | 1 | 9 |
| CNSL | CD19+22 | Relapse rates | Jing Zhou | 2024 | 1 | 1 | 1 | 0 | 1 | 1 | 1 | 0 | 1 | 1 | 1 | 9 |
| CNSL | CD20+HSCT | Relapse rates | Jing Zhou | 2024 | 1 | 1 | 1 | 0 | 1 | 1 | 1 | 0 | 1 | 1 | 1 | 9 |
| CNSL | CD20+noHSCT | Relapse rates | Jing Zhou | 2024 | 1 | 1 | 1 | 0 | 1 | 1 | 1 | 0 | 1 | 1 | 1 | 9 |
| CNSL | CD22 HSCT | Relapse rates | Jing Zhou | 2024 | 1 | 1 | 1 | 0 | 1 | 1 | 1 | 0 | 1 | 1 | 1 | 9 |
| CNSL | CD22 noHSCT | Relapse rates | Jing Zhou | 2024 | 1 | 1 | 1 | 0 | 1 | 1 | 1 | 0 | 1 | 1 | 1 | 9 |
| ALL | CD19 | CR | EmmaJ.M. Grigor | 2019 | 1 | 1 | 1 | 0 | 1 | 1 | 1 | 0 | 1 | 1 | 1 | 9 |
| CLL | CD19 | CR | EmmaJ.M. Grigor | 2019 | 1 | 1 | 1 | 0 | 1 | 1 | 1 | 0 | 1 | 1 | 1 | 9 |
| NHL | CD19 | CR | EmmaJ.M. Grigor | 2019 | 1 | 1 | 1 | 0 | 1 | 1 | 1 | 0 | 1 | 1 | 1 | 9 |
| ALL | CD22 | CR | EmmaJ.M. Grigor | 2019 | 1 | 1 | 1 | 0 | 1 | 1 | 1 | 0 | 1 | 1 | 1 | 9 |
| HL | CD30 | CR | EmmaJ.M. Grigor | 2019 | 1 | 1 | 1 | 0 | 1 | 1 | 1 | 0 | 1 | 1 | 1 | 9 |
| HL | Lewis Y | CR | EmmaJ.M. Grigor | 2019 | 1 | 1 | 1 | 0 | 1 | 1 | 1 | 0 | 1 | 1 | 1 | 9 |
| NHL | CD20 | CR | EmmaJ.M. Grigor | 2019 | 1 | 1 | 1 | 0 | 1 | 1 | 1 | 0 | 1 | 1 | 1 | 9 |
| AML | Lewis Y | CR | EmmaJ.M. Grigor | 2019 | 1 | 1 | 1 | 0 | 1 | 1 | 1 | 0 | 1 | 1 | 1 | 9 |
| ALL | CD19 | OR | EmmaJ.M. Grigor | 2019 | 1 | 1 | 1 | 0 | 1 | 1 | 1 | 0 | 1 | 1 | 1 | 9 |
| CLL | CD19 | OR | EmmaJ.M. Grigor | 2019 | 1 | 1 | 1 | 0 | 1 | 1 | 1 | 0 | 1 | 1 | 1 | 9 |
| NHL | CD19 | OR | EmmaJ.M. Grigor | 2019 | 1 | 1 | 1 | 0 | 1 | 1 | 1 | 0 | 1 | 1 | 1 | 9 |
| ALL | CD22 | OR | EmmaJ.M. Grigor | 2019 | 1 | 1 | 1 | 0 | 1 | 1 | 1 | 0 | 1 | 1 | 1 | 9 |
| HL | CD30 | OR | EmmaJ.M. Grigor | 2019 | 1 | 1 | 1 | 0 | 1 | 1 | 1 | 0 | 1 | 1 | 1 | 9 |
| HL | Lewis Y | OR | EmmaJ.M. Grigor | 2019 | 1 | 1 | 1 | 0 | 1 | 1 | 1 | 0 | 1 | 1 | 1 | 9 |
| NHL | CD20 | OR | EmmaJ.M. Grigor | 2019 | 1 | 1 | 1 | 0 | 1 | 1 | 1 | 0 | 1 | 1 | 1 | 9 |
| AML | Lewis Y | OR | EmmaJ.M. Grigor | 2019 | 1 | 1 | 1 | 0 | 1 | 1 | 1 | 0 | 1 | 1 | 1 | 9 |
| ALL | CD19 | Relapse rates | EmmaJ.M. Grigor | 2019 | 1 | 1 | 1 | 0 | 1 | 1 | 1 | 0 | 1 | 1 | 1 | 9 |
| CLL | CD19 | Relapse rates | EmmaJ.M. Grigor | 2019 | 1 | 1 | 1 | 0 | 1 | 1 | 1 | 0 | 1 | 1 | 1 | 9 |
| NHL | CD19 | Relapse rates | EmmaJ.M. Grigor | 2019 | 1 | 1 | 1 | 0 | 1 | 1 | 1 | 0 | 1 | 1 | 1 | 9 |
| ALL | CD22 | Relapse rates | EmmaJ.M. Grigor | 2019 | 1 | 1 | 1 | 0 | 1 | 1 | 1 | 0 | 1 | 1 | 1 | 9 |
| HL | CD30 | Relapse rates | EmmaJ.M. Grigor | 2019 | 1 | 1 | 1 | 0 | 1 | 1 | 1 | 0 | 1 | 1 | 1 | 9 |
| HL | Lewis Y | Relapse rates | EmmaJ.M. Grigor | 2019 | 1 | 1 | 1 | 0 | 1 | 1 | 1 | 0 | 1 | 1 | 1 | 9 |
| NHL | CD20 | Relapse rates | EmmaJ.M. Grigor | 2019 | 1 | 1 | 1 | 0 | 1 | 1 | 1 | 0 | 1 | 1 | 1 | 9 |
| ALL | CD19 | OS | EmmaJ.M. Grigor | 2019 | 1 | 1 | 1 | 0 | 1 | 1 | 1 | 0 | 1 | 1 | 1 | 9 |
| CLL | CD19 | OS | EmmaJ.M. Grigor | 2019 | 1 | 1 | 1 | 0 | 1 | 1 | 1 | 0 | 1 | 1 | 1 | 9 |
| NHL | CD19 | OS | EmmaJ.M. Grigor | 2019 | 1 | 1 | 1 | 0 | 1 | 1 | 1 | 0 | 1 | 1 | 1 | 9 |
| ALL | CD22 | OS | EmmaJ.M. Grigor | 2019 | 1 | 1 | 1 | 0 | 1 | 1 | 1 | 0 | 1 | 1 | 1 | 9 |
| HL | CD30 | OS | EmmaJ.M. Grigor | 2019 | 1 | 1 | 1 | 0 | 1 | 1 | 1 | 0 | 1 | 1 | 1 | 9 |
| HL | Lewis Y | OS | EmmaJ.M. Grigor | 2019 | 1 | 1 | 1 | 0 | 1 | 1 | 1 | 0 | 1 | 1 | 1 | 9 |
| NHL | CD20 | OS | EmmaJ.M. Grigor | 2019 | 1 | 1 | 1 | 0 | 1 | 1 | 1 | 0 | 1 | 1 | 1 | 9 |
| AML | CD20 | OS | EmmaJ.M. Grigor | 2019 | 1 | 1 | 1 | 0 | 1 | 1 | 1 | 0 | 1 | 1 | 1 | 9 |
| Hematologic malignancies | CD19 CAR-T | CRS | EmmaJ.M. Grigor | 2019 | 1 | 1 | 1 | 0 | 1 | 1 | 1 | 0 | 1 | 1 | 1 | 9 |
| Hematologic malignancies | CD19 CAR-T | Neurotoxicity | EmmaJ.M. Grigor | 2019 | 1 | 1 | 1 | 0 | 1 | 1 | 1 | 0 | 1 | 1 | 1 | 9 |
| Hematologic malignancies | CD19 CAR-T | Infection | EmmaJ.M. Grigor | 2019 | 1 | 1 | 1 | 0 | 1 | 1 | 1 | 0 | 1 | 1 | 1 | 9 |
| 129  ALL | CD19 CAR-T | GVHD | N. J. Fergusson | 2023 | 1 | 1 | 1 | 0 | 1 | 1 | 1 | 1 | 1 | 1 | 1 | 10 |
| ALL | CD19/22 CAR-T | CR | N. J. Fergusson | 2023 | 1 | 1 | 1 | 0 | 1 | 1 | 1 | 1 | 1 | 1 | 1 | 10 |
| ALL | CD22 CAR-T | CR | N. J. Fergusson | 2023 | 1 | 1 | 1 | 0 | 1 | 1 | 1 | 1 | 1 | 1 | 1 | 10 |
| NHL | CD22 CAR-T | CR | N. J. Fergusson | 2023 | 1 | 1 | 1 | 0 | 1 | 1 | 1 | 1 | 1 | 1 | 1 | 10 |
| NHL | CD19/22 CAR-T | CR | N. J. Fergusson | 2023 | 1 | 1 | 1 | 0 | 1 | 1 | 1 | 1 | 1 | 1 | 1 | 10 |
| ALL+ B-NHL+CLL | CD19 | Response rates | Hui Zhou | 2018 | 0 | 1 | 0 | 0 | 1 | 1 | 0 | 0 | 1 | 0 | 1 | 5 |
| ALL+ B-NHL+CLL | CD20 | Response rates | Hui Zhou | 2018 | 0 | 1 | 0 | 0 | 1 | 1 | 0 | 0 | 1 | 0 | 1 | 5 |
| Leukemia | CAR-T | Response rates | Hui Zhou | 2018 | 0 | 1 | 0 | 0 | 1 | 1 | 0 | 0 | 1 | 0 | 1 | 5 |
| Lymphoma | CAR-T | Response rates | Hui Zhou | 2018 | 0 | 1 | 0 | 0 | 1 | 1 | 0 | 0 | 1 | 0 | 1 | 5 |
| ALL+ B-NHL+CLL | Autogous | Response rates | Hui Zhou | 2018 | 0 | 1 | 0 | 0 | 1 | 1 | 0 | 0 | 1 | 0 | 1 | 5 |
| ALL+ B-NHL+CLL | allogeneic | Response rates | Hui Zhou | 2018 | 0 | 1 | 0 | 0 | 1 | 1 | 0 | 0 | 1 | 0 | 1 | 5 |
| ALL+ B-NHL+CLL | 1^st^ | Response rates | Hui Zhou | 2018 | 0 | 1 | 0 | 0 | 1 | 1 | 0 | 0 | 1 | 0 | 1 | 5 |
| ALL+ B-NHL+CLL | 2^nd^ | Response rates | Hui Zhou | 2018 | 0 | 1 | 0 | 0 | 1 | 1 | 0 | 0 | 1 | 0 | 1 | 5 |
| ALL+ B-NHL+CLL | 41BB+CD3ζ | Response rates | Hui Zhou | 2018 | 0 | 1 | 0 | 0 | 1 | 1 | 0 | 0 | 1 | 0 | 1 | 5 |
| ALL+ B-NHL+CLL | CD28+CD3ζ | Response rates | Hui Zhou | 2018 | 0 | 1 | 0 | 0 | 1 | 1 | 0 | 0 | 1 | 0 | 1 | 5 |
| ALL+ B-NHL+CLL | OKT3 | Response rates | Hui Zhou | 2018 | 0 | 1 | 0 | 0 | 1 | 1 | 0 | 0 | 1 | 0 | 1 | 5 |
| ALL+ B-NHL+CLL | CD3/CD28 BEADS | Response rates | Hui Zhou | 2018 | 0 | 1 | 0 | 0 | 1 | 1 | 0 | 0 | 1 | 0 | 1 | 5 |
| ALL+ B-NHL+CLL | +IL2 cells | Response rates | Hui Zhou | 2018 | 0 | 1 | 0 | 0 | 1 | 1 | 0 | 0 | 1 | 0 | 1 | 5 |
| ALL+ B-NHL+CLL | NO IL-2 cells | Response rates | Hui Zhou | 2018 | 0 | 1 | 0 | 0 | 1 | 1 | 0 | 0 | 1 | 0 | 1 | 5 |
| ALL+ B-NHL+CLL | NO viral vector | Response rates | Hui Zhou | 2018 | 0 | 1 | 0 | 0 | 1 | 1 | 0 | 0 | 1 | 0 | 1 | 5 |
| ALL+ B-NHL+CLL | viral vector | Response rates | Hui Zhou | 2018 | 0 | 1 | 0 | 0 | 1 | 1 | 0 | 0 | 1 | 0 | 1 | 5 |
| ALL+ B-NHL+CLL | lymphodepletion | Response rates | Hui Zhou | 2018 | 0 | 1 | 0 | 0 | 1 | 1 | 0 | 0 | 1 | 0 | 1 | 5 |
| ALL+ B-NHL+CLL | No lymphodepletion | Response rates | Hui Zhou | 2018 | 0 | 1 | 0 | 0 | 1 | 1 | 0 | 0 | 1 | 0 | 1 | 5 |
| ALL+ B-NHL+CLL | ≥10^8^ CAR-T cells | Response rates | Hui Zhou | 2018 | 0 | 1 | 0 | 0 | 1 | 1 | 0 | 0 | 1 | 0 | 1 | 5 |
| ALL+ B-NHL+CLL | ＜10^8^ CAR-T cells | Response rates | Hui Zhou | 2018 | 0 | 1 | 0 | 0 | 1 | 1 | 0 | 0 | 1 | 0 | 1 | 5 |
| ALL+ B-NHL+CLL | +IL2 pateints | Response rates | Hui Zhou | 2018 | 0 | 1 | 0 | 0 | 1 | 1 | 0 | 0 | 1 | 0 | 1 | 5 |
| ALL+ B-NHL+CLL | NO IL-2 pateints | Response rates | Hui Zhou | 2018 | 0 | 1 | 0 | 0 | 1 | 1 | 0 | 0 | 1 | 0 | 1 | 5 |
| ALL+ B-NHL+CLL | persistence times≥2 months | Response rates | Hui Zhou | 2018 | 0 | 1 | 0 | 0 | 1 | 1 | 0 | 0 | 1 | 0 | 1 | 5 |
| ALL+ B-NHL+CLL | persistence times＜2 months | Response rates | Hui Zhou | 2018 | 0 | 1 | 0 | 0 | 1 | 1 | 0 | 0 | 1 | 0 | 1 | 5 |
| ALL+ B-NHL+CLL | IL-2peak≥50 pg/ml | Response rates | Hui Zhou | 2018 | 0 | 1 | 0 | 0 | 1 | 1 | 0 | 0 | 1 | 0 | 1 | 5 |
| ALL+ B-NHL+CLL | IL-2peak＜50 pg/ml | Response rates | Hui Zhou | 2018 | 0 | 1 | 0 | 0 | 1 | 1 | 0 | 0 | 1 | 0 | 1 | 5 |
| ALL+ B-NHL+CLL | CD19 | 1 years PFS | Hui Zhou | 2018 | 0 | 1 | 0 | 0 | 1 | 1 | 0 | 0 | 1 | 0 | 1 | 5 |
| ALL+ B-NHL+CLL | CD20 | 1 years PFS | Hui Zhou | 2018 | 0 | 1 | 0 | 0 | 1 | 1 | 0 | 0 | 1 | 0 | 1 | 5 |
| Leukemia | CAR-T | 1 years PFS | Hui Zhou | 2018 | 0 | 1 | 0 | 0 | 1 | 1 | 0 | 0 | 1 | 0 | 1 | 5 |
| Lymphoma | CAR-T | 1 years PFS | Hui Zhou | 2018 | 0 | 1 | 0 | 0 | 1 | 1 | 0 | 0 | 1 | 0 | 1 | 5 |
| ALL+ B-NHL+CLL | Autogous | 1 years PFS | Hui Zhou | 2018 | 0 | 1 | 0 | 0 | 1 | 1 | 0 | 0 | 1 | 0 | 1 | 5 |
| ALL+ B-NHL+CLL | allogeneic | 1 years PFS | Hui Zhou | 2018 | 0 | 1 | 0 | 0 | 1 | 1 | 0 | 0 | 1 | 0 | 1 | 5 |
| ALL+ B-NHL+CLL | 1^st^ | 1 years PFS | Hui Zhou | 2018 | 0 | 1 | 0 | 0 | 1 | 1 | 0 | 0 | 1 | 0 | 1 | 5 |
| ALL+ B-NHL+CLL | 2^nd^ | 1 years PFS | Hui Zhou | 2018 | 0 | 1 | 0 | 0 | 1 | 1 | 0 | 0 | 1 | 0 | 1 | 5 |
| ALL+ B-NHL+CLL | 41BB+CD3ζ | 1 years PFS | Hui Zhou | 2018 | 0 | 1 | 0 | 0 | 1 | 1 | 0 | 0 | 1 | 0 | 1 | 5 |
| ALL+ B-NHL+CLL | CD28+CD3ζ | 1 years PFS | Hui Zhou | 2018 | 0 | 1 | 0 | 0 | 1 | 1 | 0 | 0 | 1 | 0 | 1 | 5 |
| ALL+ B-NHL+CLL | OKT3 | 1 years PFS | Hui Zhou | 2018 | 0 | 1 | 0 | 0 | 1 | 1 | 0 | 0 | 1 | 0 | 1 | 5 |
| ALL+ B-NHL+CLL | CD3/CD28 BEADS | 1 years PFS | Hui Zhou | 2018 | 0 | 1 | 0 | 0 | 1 | 1 | 0 | 0 | 1 | 0 | 1 | 5 |
| ALL+ B-NHL+CLL | +IL2 cells | 1 years PFS | Hui Zhou | 2018 | 0 | 1 | 0 | 0 | 1 | 1 | 0 | 0 | 1 | 0 | 1 | 5 |
| ALL+ B-NHL+CLL | NO IL-2 cells | 1 years PFS | Hui Zhou | 2018 | 0 | 1 | 0 | 0 | 1 | 1 | 0 | 0 | 1 | 0 | 1 | 5 |
| ALL+ B-NHL+CLL | NO viral vector | 1 years PFS | Hui Zhou | 2018 | 0 | 1 | 0 | 0 | 1 | 1 | 0 | 0 | 1 | 0 | 1 | 5 |
| ALL+ B-NHL+CLL | viral vector | 1 years PFS | Hui Zhou | 2018 | 0 | 1 | 0 | 0 | 1 | 1 | 0 | 0 | 1 | 0 | 1 | 5 |
| ALL+ B-NHL+CLL | lymphodepletion | 1 years PFS | Hui Zhou | 2018 | 0 | 1 | 0 | 0 | 1 | 1 | 0 | 0 | 1 | 0 | 1 | 5 |
| ALL+ B-NHL+CLL | No lymphodepletion | 1 years PFS | Hui Zhou | 2018 | 0 | 1 | 0 | 0 | 1 | 1 | 0 | 0 | 1 | 0 | 1 | 5 |
| ALL+ B-NHL+CLL | ≥10^8^ CAR-T cells | 1 years PFS | Hui Zhou | 2018 | 0 | 1 | 0 | 0 | 1 | 1 | 0 | 0 | 1 | 0 | 1 | 5 |
| ALL+ B-NHL+CLL | ＜10^8^ CAR-T cells | 1 years PFS | Hui Zhou | 2018 | 0 | 1 | 0 | 0 | 1 | 1 | 0 | 0 | 1 | 0 | 1 | 5 |
| ALL+ B-NHL+CLL | +IL2 pateints | 1 years PFS | Hui Zhou | 2018 | 0 | 1 | 0 | 0 | 1 | 1 | 0 | 0 | 1 | 0 | 1 | 5 |
| ALL+ B-NHL+CLL | NO IL-2 pateints | 1 years PFS | Hui Zhou | 2018 | 0 | 1 | 0 | 0 | 1 | 1 | 0 | 0 | 1 | 0 | 1 | 5 |
| ALL+ B-NHL+CLL | persistence times≥2 months | 1 years PFS | Hui Zhou | 2018 | 0 | 1 | 0 | 0 | 1 | 1 | 0 | 0 | 1 | 0 | 1 | 5 |
| ALL+ B-NHL+CLL | persistence times＜2 months | 1 years PFS | Hui Zhou | 2018 | 0 | 1 | 0 | 0 | 1 | 1 | 0 | 0 | 1 | 0 | 1 | 5 |
| ALL+ B-NHL+CLL | ≥50 pg/ml | 1 years PFS | Hui Zhou | 2018 | 0 | 1 | 0 | 0 | 1 | 1 | 0 | 0 | 1 | 0 | 1 | 5 |
| ALL+ B-NHL+CLL | ＜50 pg/ml | 1 years PFS | Hui Zhou | 2018 | 0 | 1 | 0 | 0 | 1 | 1 | 0 | 0 | 1 | 0 | 1 | 5 |
| LBCL | CD19 CAR-T | OR | Ghada Elgohary | 2024 | 0 | 1 | 1 | 0 | 1 | 1 | 1 | 0 | 1 | 1 | 1 | 8 |
| LBCL | CD19 CAR-T | CR | Ghada Elgohary | 2024 | 0 | 1 | 1 | 0 | 1 | 1 | 1 | 0 | 1 | 1 | 1 | 8 |
| LBCL | CD19 CAR-T | ICANS | Ghada Elgohary | 2024 | 0 | 1 | 1 | 0 | 1 | 1 | 1 | 0 | 1 | 1 | 1 | 8 |
| LBCL | CD19 CAR-T | CRS | Ghada Elgohary | 2024 | 0 | 1 | 1 | 0 | 1 | 1 | 1 | 0 | 1 | 1 | 1 | 8 |
| DLBCL,B-ALL,B-NHL  MM,PCL,CLL,MCL,LCBL  FL, | CD19 28Z | Severe, overall infection. | GülçinTelli Dizman | 2022 | 1 | 1 | 1 | 0 | 1 | 1 | 1 | 0 | 1 | 1 | 1 | 9 |
| DLBCL,B-ALL,B-NHL  MM,PCL,CLL,MCL,LCBL  FL, | CD19 CD27 | Severe, overall infection. | GülçinTelli Dizman | 2022 | 1 | 1 | 1 | 0 | 1 | 1 | 1 | 0 | 1 | 1 | 1 | 9 |
| CLL+ B-NHL+ALL | lymphodepletion | Response rate | Tengfei Zhang | 2015 | 0 | 1 | 1 | 0 | 1 | 1 | 1 | 0 | 1 | 1 | 1 | 8 |
| CLL+ B-NHL+ALL | Nolymphodepletion | Response rate | Tengfei Zhang | 2015 | 0 | 1 | 1 | 0 | 1 | 1 | 1 | 0 | 1 | 1 | 1 | 8 |
| CLL+ B-NHL+ALL | ≥14 days culture | Response rate | Tengfei Zhang | 2015 | 0 | 1 | 1 | 0 | 1 | 1 | 1 | 0 | 1 | 1 | 1 | 8 |
| CLL+ B-NHL+ALL | ＜14 days culture | Response rate | Tengfei Zhang | 2015 | 0 | 1 | 1 | 0 | 1 | 1 | 1 | 0 | 1 | 1 | 1 | 8 |
| CLL+ B-NHL+ALL | ≥10^7^ infused | Response rate | Tengfei Zhang | 2015 | 0 | 1 | 1 | 0 | 1 | 1 | 1 | 0 | 1 | 1 | 1 | 8 |
| CLL+ B-NHL+ALL | ＜10^7^ infused | Response rate | Tengfei Zhang | 2015 | 0 | 1 | 1 | 0 | 1 | 1 | 1 | 0 | 1 | 1 | 1 | 8 |
| CLL+ B-NHL+ALL | Adult | Response rate | Tengfei Zhang | 2015 | 0 | 1 | 1 | 0 | 1 | 1 | 1 | 0 | 1 | 1 | 1 | 8 |
| CLL+ B-NHL+ALL | Paediatric | Response rate | Tengfei Zhang | 2015 | 0 | 1 | 1 | 0 | 1 | 1 | 1 | 0 | 1 | 1 | 1 | 8 |
| CLL+ B-NHL+ALL | CD19 CAR-T | CRS | Tengfei Zhang | 2015 | 0 | 1 | 1 | 0 | 1 | 1 | 1 | 0 | 1 | 1 | 1 | 8 |
| CLL+ B-NHL+ALL | lymphodepletion | Response rate | Tengfei Zhang | 2015 | 0 | 1 | 1 | 0 | 1 | 1 | 1 | 0 | 1 | 1 | 1 | 8 |
| CLL | CD19  T culture TIME up 14 | Response rate | Emmanuel Kwateng Drokow | 2019 | 0 | 1 | 1 | 0 | 1 | 1 | 1 | 0 | 1 | 1 | 1 | 8 |
| CLL | CD19 T culture TIME up(28z retro) 14(41bb lenti) | Response rate | Emmanuel Kwateng Drokow | 2019 | 0 | 1 | 1 | 0 | 1 | 1 | 1 | 0 | 1 | 1 | 1 | 8 |
| CLL | CD19 T culture TIME down 14(41bb lenti) | Response rate | Emmanuel Kwateng Drokow | 2019 | 0 | 1 | 1 | 0 | 1 | 1 | 1 | 0 | 1 | 1 | 1 | 8 |
| B-NHL | CD19  T culture TIME up 14 | Response rate | Emmanuel Kwateng Drokow | 2019 | 0 | 1 | 1 | 0 | 1 | 1 | 1 | 0 | 1 | 1 | 1 | 8 |
| ALL | CD19  T culture TIME up 14 | Response rate | Emmanuel Kwateng Drokow | 2019 | 0 | 1 | 1 | 0 | 1 | 1 | 1 | 0 | 1 | 1 | 1 | 8 |
| NHL | CD19  T culture TIME up 14 | Response rate | Emmanuel Kwateng Drokow | 2019 | 0 | 1 | 1 | 0 | 1 | 1 | 1 | 0 | 1 | 1 | 1 | 8 |
| ALL | CD19 lyphodepletion bandamusion | Response rate | Emmanuel Kwateng Drokow | 2019 | 0 | 1 | 1 | 0 | 1 | 1 | 1 | 0 | 1 | 1 | 1 | 8 |
| ALL | CD19 cy+pen+R | Response rate | Emmanuel Kwateng Drokow | 2019 | 0 | 1 | 1 | 0 | 1 | 1 | 1 | 0 | 1 | 1 | 1 | 8 |
| ALL | CD19 flu/cy | Response rate | Emmanuel Kwateng Drokow | 2019 | 0 | 1 | 1 | 0 | 1 | 1 | 1 | 0 | 1 | 1 | 1 | 8 |
| ALL | CD19 cy+R | Response rate | Emmanuel Kwateng Drokow | 2019 | 0 | 1 | 1 | 0 | 1 | 1 | 1 | 0 | 1 | 1 | 1 | 8 |
| ALL | CD19 cy+etoposdie | Response rate | Emmanuel Kwateng Drokow | 2019 | 0 | 1 | 1 | 0 | 1 | 1 | 1 | 0 | 1 | 1 | 1 | 8 |
| ALL | CD19 no lyphodepletion | Response rate | Emmanuel Kwateng Drokow | 2019 | 0 | 1 | 1 | 0 | 1 | 1 | 1 | 0 | 1 | 1 | 1 | 8 |
| CLL | CD19  T culture TIME up 14 | CRS | Emmanuel Kwateng Drokow | 2019 | 0 | 1 | 1 | 0 | 1 | 1 | 1 | 0 | 1 | 1 | 1 | 8 |
| CLL | CD19 T culture TIME up(28z retro) 14(41bb lenti) | CRS | Emmanuel Kwateng Drokow | 2019 | 0 | 1 | 1 | 0 | 1 | 1 | 1 | 0 | 1 | 1 | 1 | 8 |
| CLL | CD19 T culture TIME down 14(41bb lenti) | CRS | Emmanuel Kwateng Drokow | 2019 | 0 | 1 | 1 | 0 | 1 | 1 | 1 | 0 | 1 | 1 | 1 | 8 |
| B-NHL | CD19  T culture TIME up 14 | CRS | Emmanuel Kwateng Drokow | 2019 | 0 | 1 | 1 | 0 | 1 | 1 | 1 | 0 | 1 | 1 | 1 | 8 |
| ALL | CD19  T culture TIME up 14 | CRS | Emmanuel Kwateng Drokow | 2019 | 0 | 1 | 1 | 0 | 1 | 1 | 1 | 0 | 1 | 1 | 1 | 8 |
| NHL | CD19  T culture TIME up 14 | CRS | Emmanuel Kwateng Drokow | 2019 | 0 | 1 | 1 | 0 | 1 | 1 | 1 | 0 | 1 | 1 | 1 | 8 |
| ALL | CD19 lyphodepletion bandamusion | CRS | Emmanuel Kwateng Drokow | 2019 | 0 | 1 | 1 | 0 | 1 | 1 | 1 | 0 | 1 | 1 | 1 | 8 |
| ALL | CD19 cy+pen+R | CRS | Emmanuel Kwateng Drokow | 2019 | 0 | 1 | 1 | 0 | 1 | 1 | 1 | 0 | 1 | 1 | 1 | 8 |
| ALL | CD19 flu/cy | CRS | Emmanuel Kwateng Drokow | 2019 | 0 | 1 | 1 | 0 | 1 | 1 | 1 | 0 | 1 | 1 | 1 | 8 |
| ALL | CD19 cy+R | CRS | Emmanuel Kwateng Drokow | 2019 | 0 | 1 | 1 | 0 | 1 | 1 | 1 | 0 | 1 | 1 | 1 | 8 |
| ALL | CD19 cy+etoposdie | CRS | Emmanuel Kwateng Drokow | 2019 | 0 | 1 | 1 | 0 | 1 | 1 | 1 | 0 | 1 | 1 | 1 | 8 |
| ALL | CD19 no lyphodepletion | CRS | Emmanuel Kwateng Drokow | 2019 | 0 | 1 | 1 | 0 | 1 | 1 | 1 | 0 | 1 | 1 | 1 | 8 |
| B- ALL | CD19 | MRD- | Sebastian Emmanuel Willyanto | 2024 | 0 | 1 | 1 | 1 | 1 | 1 | 1 | 0 | 1 | 1 | 1 | 9 |
| B- ALL | CD22 | MRD- | Sebastian Emmanuel Willyanto | 2024 | 0 | 1 | 1 | 1 | 1 | 1 | 1 | 0 | 1 | 1 | 1 | 9 |
| B- ALL | CD19/CD22 | MRD- | Sebastian Emmanuel Willyanto | 2024 | 0 | 1 | 1 | 1 | 1 | 1 | 1 | 0 | 1 | 1 | 1 | 9 |
| B- ALL | 41BB | MRD- | Sebastian Emmanuel Willyanto | 2024 | 0 | 1 | 1 | 1 | 1 | 1 | 1 | 0 | 1 | 1 | 1 | 9 |
| B- ALL | 41BB+CD3z | MRD- | Sebastian Emmanuel Willyanto | 2024 | 0 | 1 | 1 | 1 | 1 | 1 | 1 | 0 | 1 | 1 | 1 | 9 |
| B- ALL | CD28 | MRD- | Sebastian Emmanuel Willyanto | 2024 | 0 | 1 | 1 | 1 | 1 | 1 | 1 | 0 | 1 | 1 | 1 | 9 |
| B- ALL | CD3z+CD28 | MRD- | Sebastian Emmanuel Willyanto | 2024 | 0 | 1 | 1 | 1 | 1 | 1 | 1 | 0 | 1 | 1 | 1 | 9 |
| B- ALL | 4^th^ | MRD- | Sebastian Emmanuel Willyanto | 2024 | 0 | 1 | 1 | 1 | 1 | 1 | 1 | 0 | 1 | 1 | 1 | 9 |
| B- ALL | CD19 | Relapse rate | Sebastian Emmanuel Willyanto | 2024 | 0 | 1 | 1 | 1 | 1 | 1 | 1 | 0 | 1 | 1 | 1 | 9 |
| B- ALL | CD22 | Relapse rate | Sebastian Emmanuel Willyanto | 2024 | 0 | 1 | 1 | 1 | 1 | 1 | 1 | 0 | 1 | 1 | 1 | 9 |
| B- ALL | CD19/CD22 | Relapse rate | Sebastian Emmanuel Willyanto | 2024 | 0 | 1 | 1 | 1 | 1 | 1 | 1 | 0 | 1 | 1 | 1 | 9 |
| B- ALL | 41BB | Relapse rate | Sebastian Emmanuel Willyanto | 2024 | 0 | 1 | 1 | 1 | 1 | 1 | 1 | 0 | 1 | 1 | 1 | 9 |
| B- ALL | 41BB+CD3z | Relapse rate | Sebastian Emmanuel Willyanto | 2024 | 0 | 1 | 1 | 1 | 1 | 1 | 1 | 0 | 1 | 1 | 1 | 9 |
| B- ALL | CD28 | Relapse rate | Sebastian Emmanuel Willyanto | 2024 | 0 | 1 | 1 | 1 | 1 | 1 | 1 | 0 | 1 | 1 | 1 | 9 |
| B- ALL | CD3z+CD28 | Relapse rate | Sebastian Emmanuel Willyanto | 2024 | 0 | 1 | 1 | 1 | 1 | 1 | 1 | 0 | 1 | 1 | 1 | 9 |
| B- ALL | 4^th^ | Relapse rate | Sebastian Emmanuel Willyanto | 2024 | 0 | 1 | 1 | 1 | 1 | 1 | 1 | 0 | 1 | 1 | 1 | 9 |
| B- ALL | CD19 | CRS | Sebastian Emmanuel Willyanto | 2024 | 0 | 1 | 1 | 1 | 1 | 1 | 1 | 0 | 1 | 1 | 1 | 9 |
| B- ALL | CD22 | CRS | Sebastian Emmanuel Willyanto | 2024 | 0 | 1 | 1 | 1 | 1 | 1 | 1 | 0 | 1 | 1 | 1 | 9 |
| B- ALL | CD19/CD22 | CRS | Sebastian Emmanuel Willyanto | 2024 | 0 | 1 | 1 | 1 | 1 | 1 | 1 | 0 | 1 | 1 | 1 | 9 |
| B- ALL | 41BB | CRS | Sebastian Emmanuel Willyanto | 2024 | 0 | 1 | 1 | 1 | 1 | 1 | 1 | 0 | 1 | 1 | 1 | 9 |
| B- ALL | 41BB+CD3z | CRS | Sebastian Emmanuel Willyanto | 2024 | 0 | 1 | 1 | 1 | 1 | 1 | 1 | 0 | 1 | 1 | 1 | 9 |
| B- ALL | CD28 | CRS | Sebastian Emmanuel Willyanto | 2024 | 0 | 1 | 1 | 1 | 1 | 1 | 1 | 0 | 1 | 1 | 1 | 9 |
| B- ALL | CD3z+CD28 | CRS | Sebastian Emmanuel Willyanto | 2024 | 0 | 1 | 1 | 1 | 1 | 1 | 1 | 0 | 1 | 1 | 1 | 9 |
| B- ALL | 4^th^ | CRS | Sebastian Emmanuel Willyanto | 2024 | 0 | 1 | 1 | 1 | 1 | 1 | 1 | 0 | 1 | 1 | 1 | 9 |
| B- ALL | CD19 | ICANS | Sebastian Emmanuel Willyanto | 2024 | 0 | 1 | 1 | 1 | 1 | 1 | 1 | 0 | 1 | 1 | 1 | 9 |
| B- ALL | CD22 | ICANS | Sebastian Emmanuel Willyanto | 2024 | 0 | 1 | 1 | 1 | 1 | 1 | 1 | 0 | 1 | 1 | 1 | 9 |
| B- ALL | CD19/CD22 | ICANS | Sebastian Emmanuel Willyanto | 2024 | 0 | 1 | 1 | 1 | 1 | 1 | 1 | 0 | 1 | 1 | 1 | 9 |
| B- ALL | 41BB | ICANS | Sebastian Emmanuel Willyanto | 2024 | 0 | 1 | 1 | 1 | 1 | 1 | 1 | 0 | 1 | 1 | 1 | 9 |
| B- ALL | 41BB+CD3z | ICANS | Sebastian Emmanuel Willyanto | 2024 | 0 | 1 | 1 | 1 | 1 | 1 | 1 | 0 | 1 | 1 | 1 | 9 |
| B- ALL | CD28 | ICANS | Sebastian Emmanuel Willyanto | 2024 | 0 | 1 | 1 | 1 | 1 | 1 | 1 | 0 | 1 | 1 | 1 | 9 |
| B- ALL | CD3z+CD28 | ICANS | Sebastian Emmanuel Willyanto | 2024 | 0 | 1 | 1 | 1 | 1 | 1 | 1 | 0 | 1 | 1 | 1 | 9 |
| B- ALL | 4^th^ | ICANS | Sebastian Emmanuel Willyanto | 2024 | 0 | 1 | 1 | 1 | 1 | 1 | 1 | 0 | 1 | 1 | 1 | 9 |
| GCB n-GCB HGBL | CD19 T infused up 10e7 | ORR OS EFS HR RR | Liat Shargian | 2023 | 0 | 1 | 1 | 0 | 1 | 1 | 1 | 0 | 1 | 0 | 1 | 7 |
| GCB n-GCB HGBL | CD19 T infused down 10e7 | ORR OS EFS HR RR | Liat Shargian | 2023 | 0 | 1 | 1 | 0 | 1 | 1 | 1 | 0 | 1 | 0 | 1 | 7 |
| ALL | CD19/22 CAR-T | Overall response | ThiThuy Nguyen | 2022 | 1 | 1 | 1 | 0 | 1 | 1 | 1 | 0 | 1 | 1 | 1 | 10 |
| ALL | CD19/22 CAR-T | CR | ThiThuy Nguyen | 2022 | 1 | 1 | 1 | 0 | 1 | 1 | 1 | 0 | 1 | 1 | 1 | 10 |
| NHL | CD19/22 CAR-T | Overall response | ThiThuy Nguyen | 2022 | 1 | 1 | 1 | 0 | 1 | 1 | 1 | 0 | 1 | 1 | 1 | 10 |
| NHL | CD19/22 CAR-T | CR | ThiThuy Nguyen | 2022 | 1 | 1 | 1 | 0 | 1 | 1 | 1 | 0 | 1 | 1 | 1 | 10 |
| ALL | CD19/22 CAR-T | CRS | ThiThuy Nguyen | 2022 | 1 | 1 | 1 | 0 | 1 | 1 | 1 | 0 | 1 | 1 | 1 | 10 |
| ALL | CD19/22 CAR-T | sCRS | ThiThuy Nguyen | 2022 | 1 | 1 | 1 | 0 | 1 | 1 | 1 | 0 | 1 | 1 | 1 | 10 |
| ALL | CD19/22 CAR-T | Neurotoxicity | ThiThuy Nguyen | 2022 | 1 | 1 | 1 | 0 | 1 | 1 | 1 | 0 | 1 | 1 | 1 | 10 |
| NHL | CD19/22 CAR-T | CRS | ThiThuy Nguyen | 2022 | 1 | 1 | 1 | 0 | 1 | 1 | 1 | 0 | 1 | 1 | 1 | 10 |
| NHL | CD19/22 CAR-T | sCRS | ThiThuy Nguyen | 2022 | 1 | 1 | 1 | 0 | 1 | 1 | 1 | 0 | 1 | 1 | 1 | 10 |
| NHL | CD19/22 CAR-T | Neurotoxicity | ThiThuy Nguyen | 2022 | 1 | 1 | 1 | 0 | 1 | 1 | 1 | 0 | 1 | 1 | 1 | 10 |
| ALL | CD19/22 CAR-T | Overall response | ThiThuy Nguyen | 2022 | 1 | 1 | 1 | 0 | 1 | 1 | 1 | 0 | 1 | 1 | 1 | 10 |
| ALL | CD19+22 CAR-T | Early infections | Gemma K. Reynolds | 2023 | 1 | 1 | 1 | 0 | 1 | 1 | 1 | 0 | 1 | 1 | 1 | 9 |
| ALL | CD19+22 CAR-T | late infections | Gemma K. Reynolds | 2023 | 1 | 1 | 1 | 0 | 1 | 1 | 1 | 0 | 1 | 1 | 1 | 9 |
| NHL | CD19+22 CAR-T | Early infections | Gemma K. Reynolds | 2023 | 1 | 1 | 1 | 0 | 1 | 1 | 1 | 0 | 1 | 1 | 1 | 9 |
| NHL | CD19+22 CAR-T | late infections | Gemma K. Reynolds | 2023 | 1 | 1 | 1 | 0 | 1 | 1 | 1 | 0 | 1 | 1 | 1 | 9 |
| NHL | CD19+IL-6 | CRS | Baitao Dou | 2024 | 1 | 1 | 1 | 0 | 1 | 1 | 1 | 0 | 1 | 1 | 1 | 10 |
| B‑cell lymphoma | CD19,CD20 CAR-T | ORR | MUBARAK AL‑MANSOUR | 2020 | 0 | 1 | 1 | 0 | 0 | 1 | 1 | 0 | 1 | 0 | 1 | 6 |
| B‑cell lymphoma | CD19,CD20 CAR-T | CR | MUBARAK AL‑MANSOUR | 2020 | 0 | 1 | 1 | 0 | 0 | 1 | 1 | 0 | 1 | 0 | 1 | 6 |
| B‑cell lymphoma | CD19,CD20 CAR-T | Anemia | MUBARAK AL‑MANSOUR | 2020 | 0 | 1 | 1 | 0 | 0 | 1 | 1 | 0 | 1 | 0 | 1 | 6 |
| B‑cell lymphoma | CD19,CD20 CAR-T | CRS | MUBARAK AL‑MANSOUR | 2020 | 0 | 1 | 1 | 0 | 0 | 1 | 1 | 0 | 1 | 0 | 1 | 6 |
| B‑cell lymphoma | CD19,CD20 CAR-T | Neutropenia | MUBARAK AL‑MANSOUR | 2020 | 0 | 1 | 1 | 0 | 0 | 1 | 1 | 0 | 1 | 0 | 1 | 6 |
| B‑cell lymphoma | CD19,CD20 CAR-T | Neurotoxicity | MUBARAK AL‑MANSOUR | 2020 | 0 | 1 | 1 | 0 | 0 | 1 | 1 | 0 | 1 | 0 | 1 | 6 |
| B‑cell lymphoma | CD19,CD20 CAR-T | Thrombocytopenia | MUBARAK AL‑MANSOUR | 2020 | 0 | 1 | 1 | 0 | 0 | 1 | 1 | 0 | 1 | 0 | 1 | 6 |
| R/R DLBCL | CD20 lyphodepletion CHODE | CR | H. H. Cao | 2020 | 0 | 1 | 1 | 0 | 1 | 1 | 1 | 0 | 1 | 1 | 1 | 8 |
| R/R DLBCL | CD20 lyphodepletion ESHAP | CR | H. H. Cao | 2020 | 0 | 1 | 1 | 0 | 1 | 1 | 1 | 0 | 1 | 1 | 1 | 8 |
| R/R DLBCL | CD20 lyphodepletion COD | CR | H. H. Cao | 2020 | 0 | 1 | 1 | 0 | 1 | 1 | 1 | 0 | 1 | 1 | 1 | 8 |
| R/R DLBCL | CD20 lyphodepletion COED | CR | H. H. Cao | 2020 | 0 | 1 | 1 | 0 | 1 | 1 | 1 | 0 | 1 | 1 | 1 | 8 |
| R/R DLBCL | CD20 lyphodepletion cy | CR | H. H. Cao | 2020 | 0 | 1 | 1 | 0 | 1 | 1 | 1 | 0 | 1 | 1 | 1 | 8 |
| R/R DLBCL | CD20 T infused up 10e7 | CR | H. H. Cao | 2020 | 0 | 1 | 1 | 0 | 1 | 1 | 1 | 0 | 1 | 1 | 1 | 8 |
| R/R DLBCL | CD20 T infused down 10e7 | CR | H. H. Cao | 2020 | 0 | 1 | 1 | 0 | 1 | 1 | 1 | 0 | 1 | 1 | 1 | 8 |
| S/PCSL | CD19+CD22 | ORR | Liwei Lv | 2023 | 1 | 1 | 1 | 0 | 1 | 1 | 1 | 0 | 1 | 1 | 1 | 8 |
| S/PCSL | CD19+CD22 | CR | Liwei Lv | 2023 | 1 | 1 | 1 | 0 | 1 | 1 | 1 | 0 | 1 | 1 | 1 | 8 |
| S/PCSL | CD19+CD22 | PR | Liwei Lv | 2023 | 1 | 1 | 1 | 0 | 1 | 1 | 1 | 0 | 1 | 1 | 1 | 8 |
| S/PCSL | CD19+CD22 | PD | Liwei Lv | 2023 | 1 | 1 | 1 | 0 | 1 | 1 | 1 | 0 | 1 | 1 | 1 | 8 |
| PCSL | CD19+CD22 | ORR | Liwei Lv | 2023 | 1 | 1 | 1 | 0 | 1 | 1 | 1 | 0 | 1 | 1 | 1 | 8 |
| PCSL | CD19+CD22 | CR | Liwei Lv | 2023 | 1 | 1 | 1 | 0 | 1 | 1 | 1 | 0 | 1 | 1 | 1 | 8 |
| SCSL | CD19+CD22 | ORR | Liwei Lv | 2023 | 1 | 1 | 1 | 0 | 1 | 1 | 1 | 0 | 1 | 1 | 1 | 8 |
| SCSL | CD19+CD22 | CR | Liwei Lv | 2023 | 1 | 1 | 1 | 0 | 1 | 1 | 1 | 0 | 1 | 1 | 1 | 8 |
| S/PCSL | CD19+CD22 | OS | Liwei Lv | 2023 | 1 | 1 | 1 | 0 | 1 | 1 | 1 | 0 | 1 | 1 | 1 | 8 |
| S/PCSL | CD19+CD22 | PFS | Liwei Lv | 2023 | 1 | 1 | 1 | 0 | 1 | 1 | 1 | 0 | 1 | 1 | 1 | 8 |
| S/PCSL | CD19+CD22 | 1-2 CRS | Liwei Lv | 2023 | 1 | 1 | 1 | 0 | 1 | 1 | 1 | 0 | 1 | 1 | 1 | 8 |
| S/PCSL | CD19+CD22 | 1-2 neurotoxicity | Liwei Lv | 2023 | 1 | 1 | 1 | 0 | 1 | 1 | 1 | 0 | 1 | 1 | 1 | 8 |
| S/PCSL | CD19+CD22 | ≥3 neurotoxicity | Liwei Lv | 2023 | 1 | 1 | 1 | 0 | 1 | 1 | 1 | 0 | 1 | 1 | 1 | 8 |
| B-NHL | Axi-Cel | Any CRS | Samuel Yamshon | 2024 | 1 | 1 | 1 | 0 | 1 | 1 | 1 | 0 | 1 | 1 | 1 | 9 |
| B-NHL | Liso-Cel | Any CRS | Samuel Yamshon | 2024 | 1 | 1 | 1 | 0 | 1 | 1 | 1 | 0 | 1 | 1 | 1 | 9 |
| B-NHL | Tiso-Cel | Any CRS | Samuel Yamshon | 2024 | 1 | 1 | 1 | 0 | 1 | 1 | 1 | 0 | 1 | 1 | 1 | 9 |
| B-NHL | Axi-Cel | ≥3 CRS | Samuel Yamshon | 2024 | 1 | 1 | 1 | 0 | 1 | 1 | 1 | 0 | 1 | 1 | 1 | 9 |
| B-NHL | Liso-Cel | ≥3 CRS | Samuel Yamshon | 2024 | 1 | 1 | 1 | 0 | 1 | 1 | 1 | 0 | 1 | 1 | 1 | 9 |
| B-NHL | Tiso-Cel | ≥3 CRS | Samuel Yamshon | 2024 | 1 | 1 | 1 | 0 | 1 | 1 | 1 | 0 | 1 | 1 | 1 | 9 |
| B-NHL | Axi-Cel | Any ICANS | Samuel Yamshon | 2024 | 1 | 1 | 1 | 0 | 1 | 1 | 1 | 0 | 1 | 1 | 1 | 9 |
| B-NHL | Liso-Cel | Any ICANS | Samuel Yamshon | 2024 | 1 | 1 | 1 | 0 | 1 | 1 | 1 | 0 | 1 | 1 | 1 | 9 |
| B-NHL | Tiso-Cel | Any ICANS | Samuel Yamshon | 2024 | 1 | 1 | 1 | 0 | 1 | 1 | 1 | 0 | 1 | 1 | 1 | 9 |
| B-NHL | Axi-Cel | ≥3 ICANS | Samuel Yamshon | 2024 | 1 | 1 | 1 | 0 | 1 | 1 | 1 | 0 | 1 | 1 | 1 | 9 |
| B-NHL | Liso-Cel | ≥3 ICANS | Samuel Yamshon | 2024 | 1 | 1 | 1 | 0 | 1 | 1 | 1 | 0 | 1 | 1 | 1 | 9 |
| B-NHL | Tiso-Cel | ≥3 ICANS | Samuel Yamshon | 2024 | 1 | 1 | 1 | 0 | 1 | 1 | 1 | 0 | 1 | 1 | 1 | 9 |
| B-NHL | Axi-Cel | Any thrombocytopenia | Samuel Yamshon | 2024 | 1 | 1 | 1 | 0 | 1 | 1 | 1 | 0 | 1 | 1 | 1 | 9 |
| B-NHL | Liso-Cel | Any thrombocytopenia | Samuel Yamshon | 2024 | 1 | 1 | 1 | 0 | 1 | 1 | 1 | 0 | 1 | 1 | 1 | 9 |
| B-NHL | Tiso-Cel | Any thrombocytopenia | Samuel Yamshon | 2024 | 1 | 1 | 1 | 0 | 1 | 1 | 1 | 0 | 1 | 1 | 1 | 9 |
| B-NHL | Axi-Cel | ≥3 thrombocytopenia | Samuel Yamshon | 2024 | 1 | 1 | 1 | 0 | 1 | 1 | 1 | 0 | 1 | 1 | 1 | 9 |
| B-NHL | Liso-Cel | ≥3 thrombocytopenia | Samuel Yamshon | 2024 | 1 | 1 | 1 | 0 | 1 | 1 | 1 | 0 | 1 | 1 | 1 | 9 |
| B-NHL | Tiso-Cel | ≥3 thrombocytopenia | Samuel Yamshon | 2024 | 1 | 1 | 1 | 0 | 1 | 1 | 1 | 0 | 1 | 1 | 1 | 9 |
| B-NHL | Axi-Cel | Any neutropenia | Samuel Yamshon | 2024 | 1 | 1 | 1 | 0 | 1 | 1 | 1 | 0 | 1 | 1 | 1 | 9 |
| B-NHL | Liso-Cel | Any neutropenia | Samuel Yamshon | 2024 | 1 | 1 | 1 | 0 | 1 | 1 | 1 | 0 | 1 | 1 | 1 | 9 |
| B-NHL | Tiso-Cel | Any neutropenia | Samuel Yamshon | 2024 | 1 | 1 | 1 | 0 | 1 | 1 | 1 | 0 | 1 | 1 | 1 | 9 |
| B-NHL | Axi-Cel | ≥3 neutropenia | Samuel Yamshon | 2024 | 1 | 1 | 1 | 0 | 1 | 1 | 1 | 0 | 1 | 1 | 1 | 9 |
| B-NHL | Liso-Cel | ≥3 neutropenia | Samuel Yamshon | 2024 | 1 | 1 | 1 | 0 | 1 | 1 | 1 | 0 | 1 | 1 | 1 | 9 |
| B-NHL | Tiso-Cel | ≥3 neutropenia | Samuel Yamshon | 2024 | 1 | 1 | 1 | 0 | 1 | 1 | 1 | 0 | 1 | 1 | 1 | 9 |
| B-NHL | Axi-Cel | Any infection | Samuel Yamshon | 2024 | 1 | 1 | 1 | 0 | 1 | 1 | 1 | 0 | 1 | 1 | 1 | 9 |
| B-NHL | Liso-Cel | Any infection | Samuel Yamshon | 2024 | 1 | 1 | 1 | 0 | 1 | 1 | 1 | 0 | 1 | 1 | 1 | 9 |
| B-NHL | Tiso-Cel | Any infection | Samuel Yamshon | 2024 | 1 | 1 | 1 | 0 | 1 | 1 | 1 | 0 | 1 | 1 | 1 | 9 |
| B-NHL | Axi-Cel | ≥3 infection | Samuel Yamshon | 2024 | 1 | 1 | 1 | 0 | 1 | 1 | 1 | 0 | 1 | 1 | 1 | 9 |
| B-NHL | Liso-Cel | ≥3 infection | Samuel Yamshon | 2024 | 1 | 1 | 1 | 0 | 1 | 1 | 1 | 0 | 1 | 1 | 1 | 9 |
| B-NHL | Tiso-Cel | ≥3 infection | Samuel Yamshon | 2024 | 1 | 1 | 1 | 0 | 1 | 1 | 1 | 0 | 1 | 1 | 1 | 9 |
| B-NHL | Axi-Cel | febrile neutropenia | Samuel Yamshon | 2024 | 1 | 1 | 1 | 0 | 1 | 1 | 1 | 0 | 1 | 1 | 1 | 9 |
| B-NHL | Liso-Cel | febrile neutropenia | Samuel Yamshon | 2024 | 1 | 1 | 1 | 0 | 1 | 1 | 1 | 0 | 1 | 1 | 1 | 9 |
| B-NHL | Tiso-Cel | febrile neutropenia | Samuel Yamshon | 2024 | 1 | 1 | 1 | 0 | 1 | 1 | 1 | 0 | 1 | 1 | 1 | 9 |
| NHL | CD19 CAR-T | any CRS | Wen Lei | 2021 | 0 | 1 | 1 | 0 | 1 | 1 | 1 | 0 | 1 | 1 | 1 | 8 |
| ALL | CD19 CAR-T | any CRS | Wen Lei | 2021 | 0 | 1 | 1 | 0 | 1 | 1 | 1 | 0 | 1 | 1 | 1 | 8 |
| NHL+ALL | USA | any CRS | Wen Lei | 2021 | 0 | 1 | 1 | 0 | 1 | 1 | 1 | 0 | 1 | 1 | 1 | 8 |
| NHL+ALL | CHINA | any CRS | Wen Lei | 2021 | 0 | 1 | 1 | 0 | 1 | 1 | 1 | 0 | 1 | 1 | 1 | 8 |
| NHL+ALL | Adult | any CRS | Wen Lei | 2021 | 0 | 1 | 1 | 0 | 1 | 1 | 1 | 0 | 1 | 1 | 1 | 8 |
| NHL+ALL | Young | any CRS | Wen Lei | 2021 | 0 | 1 | 1 | 0 | 1 | 1 | 1 | 0 | 1 | 1 | 1 | 8 |
| NHL+ALL | Auto | any CRS | Wen Lei | 2021 | 0 | 1 | 1 | 0 | 1 | 1 | 1 | 0 | 1 | 1 | 1 | 8 |
| NHL+ALL | Allo | any CRS | Wen Lei | 2021 | 0 | 1 | 1 | 0 | 1 | 1 | 1 | 0 | 1 | 1 | 1 | 8 |
| NHL+ALL | 41BB+CD28 | any CRS | Wen Lei | 2021 | 0 | 1 | 1 | 0 | 1 | 1 | 1 | 0 | 1 | 1 | 1 | 8 |
| NHL+ALL | CD28 | any CRS | Wen Lei | 2021 | 0 | 1 | 1 | 0 | 1 | 1 | 1 | 0 | 1 | 1 | 1 | 8 |
| NHL+ALL | 41BB | any CRS | Wen Lei | 2021 | 0 | 1 | 1 | 0 | 1 | 1 | 1 | 0 | 1 | 1 | 1 | 8 |
| NHL+ALL | lenti | any CRS | Wen Lei | 2021 | 0 | 1 | 1 | 0 | 1 | 1 | 1 | 0 | 1 | 1 | 1 | 8 |
| NHL+ALL | Gamm | any CRS | Wen Lei | 2021 | 0 | 1 | 1 | 0 | 1 | 1 | 1 | 0 | 1 | 1 | 1 | 8 |
| NHL+ALL | Retro | any CRS | Wen Lei | 2021 | 0 | 1 | 1 | 0 | 1 | 1 | 1 | 0 | 1 | 1 | 1 | 8 |
| NHL+ALL | Cy | any CRS | Wen Lei | 2021 | 0 | 1 | 1 | 0 | 1 | 1 | 1 | 0 | 1 | 1 | 1 | 8 |
| NHL+ALL | C+yFlu | any CRS | Wen Lei | 2021 | 0 | 1 | 1 | 0 | 1 | 1 | 1 | 0 | 1 | 1 | 1 | 8 |
| NHL+ALL | ＜10^6^ | any CRS | Wen Lei | 2021 | 0 | 1 | 1 | 0 | 1 | 1 | 1 | 0 | 1 | 1 | 1 | 8 |
| NHL+ALL | 10^6^ | any CRS | Wen Lei | 2021 | 0 | 1 | 1 | 0 | 1 | 1 | 1 | 0 | 1 | 1 | 1 | 8 |
| NHL+ALL | ＞10^6^ | any CRS | Wen Lei | 2021 | 0 | 1 | 1 | 0 | 1 | 1 | 1 | 0 | 1 | 1 | 1 | 8 |
| NHL | CD19 CAR-T | any NS | Wen Lei | 2021 | 0 | 1 | 1 | 0 | 1 | 1 | 1 | 0 | 1 | 1 | 1 | 8 |
| ALL | CD19 CAR-T | any NS | Wen Lei | 2021 | 0 | 1 | 1 | 0 | 1 | 1 | 1 | 0 | 1 | 1 | 1 | 8 |
| NHL+ALL | USA | any NS | Wen Lei | 2021 | 0 | 1 | 1 | 0 | 1 | 1 | 1 | 0 | 1 | 1 | 1 | 8 |
| NHL+ALL | CHINA | any NS | Wen Lei | 2021 | 0 | 1 | 1 | 0 | 1 | 1 | 1 | 0 | 1 | 1 | 1 | 8 |
| NHL+ALL | Adult | any NS | Wen Lei | 2021 | 0 | 1 | 1 | 0 | 1 | 1 | 1 | 0 | 1 | 1 | 1 | 8 |
| NHL+ALL | Young | any NS | Wen Lei | 2021 | 0 | 1 | 1 | 0 | 1 | 1 | 1 | 0 | 1 | 1 | 1 | 8 |
| NHL+ALL | Auto | any NS | Wen Lei | 2021 | 0 | 1 | 1 | 0 | 1 | 1 | 1 | 0 | 1 | 1 | 1 | 8 |
| NHL+ALL | Allo | any NS | Wen Lei | 2021 | 0 | 1 | 1 | 0 | 1 | 1 | 1 | 0 | 1 | 1 | 1 | 8 |
| NHL+ALL | 41BB | any NS | Wen Lei | 2021 | 0 | 1 | 1 | 0 | 1 | 1 | 1 | 0 | 1 | 1 | 1 | 8 |
| NHL+ALL | CD28 | any NS | Wen Lei | 2021 | 0 | 1 | 1 | 0 | 1 | 1 | 1 | 0 | 1 | 1 | 1 | 8 |
| NHL+ALL | Gamm | any NS | Wen Lei | 2021 | 0 | 1 | 1 | 0 | 1 | 1 | 1 | 0 | 1 | 1 | 1 | 8 |
| NHL+ALL | lenti | any NS | Wen Lei | 2021 | 0 | 1 | 1 | 0 | 1 | 1 | 1 | 0 | 1 | 1 | 1 | 8 |
| NHL+ALL | Retro | any NS | Wen Lei | 2021 | 0 | 1 | 1 | 0 | 1 | 1 | 1 | 0 | 1 | 1 | 1 | 8 |
| NHL+ALL | Cy | any NS | Wen Lei | 2021 | 0 | 1 | 1 | 0 | 1 | 1 | 1 | 0 | 1 | 1 | 1 | 8 |
| NHL+ALL | C+yFlu | any NS | Wen Lei | 2021 | 0 | 1 | 1 | 0 | 1 | 1 | 1 | 0 | 1 | 1 | 1 | 8 |
| NHL+ALL | ＜10^6^ | any NS | Wen Lei | 2021 | 0 | 1 | 1 | 0 | 1 | 1 | 1 | 0 | 1 | 1 | 1 | 8 |
| NHL+ALL | 10^6^ | any NS | Wen Lei | 2021 | 0 | 1 | 1 | 0 | 1 | 1 | 1 | 0 | 1 | 1 | 1 | 8 |
| NHL | CD19 CAR-T | ≥3 CRS | Wen Lei | 2021 | 0 | 1 | 1 | 0 | 1 | 1 | 1 | 0 | 1 | 1 | 1 | 8 |
| ALL | CD19 CAR-T | ≥3 CRS | Wen Lei | 2021 | 0 | 1 | 1 | 0 | 1 | 1 | 1 | 0 | 1 | 1 | 1 | 8 |
| NHL+ALL | USA | ≥3 CRS | Wen Lei | 2021 | 0 | 1 | 1 | 0 | 1 | 1 | 1 | 0 | 1 | 1 | 1 | 8 |
| NHL+ALL | CHINA | ≥3 CRS | Wen Lei | 2021 | 0 | 1 | 1 | 0 | 1 | 1 | 1 | 0 | 1 | 1 | 1 | 8 |
| NHL+ALL | Adult | ≥3 CRS | Wen Lei | 2021 | 0 | 1 | 1 | 0 | 1 | 1 | 1 | 0 | 1 | 1 | 1 | 8 |
| NHL+ALL | Young | ≥3 CRS | Wen Lei | 2021 | 0 | 1 | 1 | 0 | 1 | 1 | 1 | 0 | 1 | 1 | 1 | 8 |
| NHL+ALL | Auto | ≥3 CRS | Wen Lei | 2021 | 0 | 1 | 1 | 0 | 1 | 1 | 1 | 0 | 1 | 1 | 1 | 8 |
| NHL+ALL | Allo | ≥3 CRS | Wen Lei | 2021 | 0 | 1 | 1 | 0 | 1 | 1 | 1 | 0 | 1 | 1 | 1 | 8 |
| NHL+ALL | 41BB+CD28 | ≥3 CRS | Wen Lei | 2021 | 0 | 1 | 1 | 0 | 1 | 1 | 1 | 0 | 1 | 1 | 1 | 8 |
| NHL+ALL | CD28 | ≥3 CRS | Wen Lei | 2021 | 0 | 1 | 1 | 0 | 1 | 1 | 1 | 0 | 1 | 1 | 1 | 8 |
| NHL+ALL | 41BB | ≥3 CRS | Wen Lei | 2021 | 0 | 1 | 1 | 0 | 1 | 1 | 1 | 0 | 1 | 1 | 1 | 8 |
| NHL+ALL | Gamm | ≥3 CRS | Wen Lei | 2021 | 0 | 1 | 1 | 0 | 1 | 1 | 1 | 0 | 1 | 1 | 1 | 8 |
| NHL+ALL | lenti | ≥3 CRS | Wen Lei | 2021 | 0 | 1 | 1 | 0 | 1 | 1 | 1 | 0 | 1 | 1 | 1 | 8 |
| NHL+ALL | Retro | ≥3 CRS | Wen Lei | 2021 | 0 | 1 | 1 | 0 | 1 | 1 | 1 | 0 | 1 | 1 | 1 | 8 |
| NHL+ALL | Cy | ≥3 CRS | Wen Lei | 2021 | 0 | 1 | 1 | 0 | 1 | 1 | 1 | 0 | 1 | 1 | 1 | 8 |
| NHL+ALL | C+yFlu | ≥3 CRS | Wen Lei | 2021 | 0 | 1 | 1 | 0 | 1 | 1 | 1 | 0 | 1 | 1 | 1 | 8 |
| NHL+ALL | ＜10^6^ | ≥3 CRS | Wen Lei | 2021 | 0 | 1 | 1 | 0 | 1 | 1 | 1 | 0 | 1 | 1 | 1 | 8 |
| NHL+ALL | 10^6^ | ≥3 CRS | Wen Lei | 2021 | 0 | 1 | 1 | 0 | 1 | 1 | 1 | 0 | 1 | 1 | 1 | 8 |
| NHL+ALL | ＞10^6^ | ≥3 CRS | Wen Lei | 2021 | 0 | 1 | 1 | 0 | 1 | 1 | 1 | 0 | 1 | 1 | 1 | 8 |
| NHL | CD19 CAR-T | ≥3 NS | Wen Lei | 2021 | 0 | 1 | 1 | 0 | 1 | 1 | 1 | 0 | 1 | 1 | 1 | 8 |
| ALL | CD19 CAR-T | ≥3 NS | Wen Lei | 2021 | 0 | 1 | 1 | 0 | 1 | 1 | 1 | 0 | 1 | 1 | 1 | 8 |
| NHL+ALL | USA | ≥3 NS | Wen Lei | 2021 | 0 | 1 | 1 | 0 | 1 | 1 | 1 | 0 | 1 | 1 | 1 | 8 |
| NHL+ALL | CHINA | ≥3 NS | Wen Lei | 2021 | 0 | 1 | 1 | 0 | 1 | 1 | 1 | 0 | 1 | 1 | 1 | 8 |
| NHL+ALL | Adult | ≥3 NS | Wen Lei | 2021 | 0 | 1 | 1 | 0 | 1 | 1 | 1 | 0 | 1 | 1 | 1 | 8 |
| NHL+ALL | Young | ≥3 NS | Wen Lei | 2021 | 0 | 1 | 1 | 0 | 1 | 1 | 1 | 0 | 1 | 1 | 1 | 8 |
| NHL+ALL | Auto | ≥3 NS | Wen Lei | 2021 | 0 | 1 | 1 | 0 | 1 | 1 | 1 | 0 | 1 | 1 | 1 | 8 |
| NHL+ALL | Allo | ≥3 NS | Wen Lei | 2021 | 0 | 1 | 1 | 0 | 1 | 1 | 1 | 0 | 1 | 1 | 1 | 8 |
| NHL+ALL | 41BB | ≥3 NS | Wen Lei | 2021 | 0 | 1 | 1 | 0 | 1 | 1 | 1 | 0 | 1 | 1 | 1 | 8 |
| NHL+ALL | CD28 | ≥3 NS | Wen Lei | 2021 | 0 | 1 | 1 | 0 | 1 | 1 | 1 | 0 | 1 | 1 | 1 | 8 |
| NHL+ALL | Gamm | ≥3 NS | Wen Lei | 2021 | 0 | 1 | 1 | 0 | 1 | 1 | 1 | 0 | 1 | 1 | 1 | 8 |
| NHL+ALL | lenti | ≥3 NS | Wen Lei | 2021 | 0 | 1 | 1 | 0 | 1 | 1 | 1 | 0 | 1 | 1 | 1 | 8 |
| NHL+ALL | Retro | ≥3 NS | Wen Lei | 2021 | 0 | 1 | 1 | 0 | 1 | 1 | 1 | 0 | 1 | 1 | 1 | 8 |
| NHL+ALL | Cy | ≥3 NS | Wen Lei | 2021 | 0 | 1 | 1 | 0 | 1 | 1 | 1 | 0 | 1 | 1 | 1 | 8 |
| NHL+ALL | C+yFlu | any NS | Wen Lei | 2021 | 0 | 1 | 1 | 0 | 1 | 1 | 1 | 0 | 1 | 1 | 1 | 8 |
| NHL+ALL | ＜10^6^ | any NS | Wen Lei | 2021 | 0 | 1 | 1 | 0 | 1 | 1 | 1 | 0 | 1 | 1 | 1 | 8 |
| NHL+ALL | 10^6^ | any NS | Wen Lei | 2021 | 0 | 1 | 1 | 0 | 1 | 1 | 1 | 0 | 1 | 1 | 1 | 8 |
| NHL+ALL | ＞10^6^ | any NS | Wen Lei | 2021 | 0 | 1 | 1 | 0 | 1 | 1 | 1 | 0 | 1 | 1 | 1 | 8 |
| R/RALL | CD19 allogenic at TRAC and CD52 locus | CR 1year-OS CRS  GVHD Nt | Sifei Chen | 2022 | 1 | 1 | 1 | 0 | 1 | 1 | 1 | 1 | 1 | 1 | 1 | 10 |
| R/RALL | CD19 allogenic at TRAC locus | CR 1year-OS CRS  GVHD Nt | Sifei Chen | 2022 | 1 | 1 | 1 | 0 | 1 | 1 | 1 | 1 | 1 | 1 | 1 | 10 |
| NHL | CD19 allogenic at TRAC and CD52 locus | CR 1year-OS CRS  GVHD Nt | Sifei Chen | 2022 | 1 | 1 | 1 | 0 | 1 | 1 | 1 | 1 | 1 | 1 | 1 | 10 |
| NHL | CD19 allogenic at TRAC locus | CR 1year-OS CRS  GVHD Nt | Sifei Chen | 2022 | 1 | 1 | 1 | 0 | 1 | 1 | 1 | 1 | 1 | 1 | 1 | 10 |
| ALL | CD19/CD22 | CR | Jared Becerril-Rico | 2023 | 0 | 1 | 1 | 0 | 1 | 1 | 1 | 0 | 1 | 0 | 1 | 7 |
| ALL | CD19 | CR | Jared Becerril-Rico | 2023 | 0 | 1 | 1 | 0 | 1 | 1 | 1 | 0 | 1 | 0 | 1 | 7 |
| ALL | CD22 | CR | Jared Becerril-Rico | 2023 | 0 | 1 | 1 | 0 | 1 | 1 | 1 | 0 | 1 | 0 | 1 | 7 |
| ALL | CD19/CD22 | MRD- | Jared Becerril-Rico | 2023 | 0 | 1 | 1 | 0 | 1 | 1 | 1 | 0 | 1 | 0 | 1 | 7 |
| ALL | CD19 | MRD- | Jared Becerril-Rico | 2023 | 0 | 1 | 1 | 0 | 1 | 1 | 1 | 0 | 1 | 0 | 1 | 7 |
| ALL | CD22 | MRD- | Jared Becerril-Rico | 2023 | 0 | 1 | 1 | 0 | 1 | 1 | 1 | 0 | 1 | 0 | 1 | 7 |
| ALL | CD19/CD22 | 1 year OS | Jared Becerril-Rico | 2023 | 0 | 1 | 1 | 0 | 1 | 1 | 1 | 0 | 1 | 0 | 1 | 7 |
| ALL | CD19 | 1 year OS | Jared Becerril-Rico | 2023 | 0 | 1 | 1 | 0 | 1 | 1 | 1 | 0 | 1 | 0 | 1 | 7 |
| ALL | CD19/CD22 | 1 year RFS Relapse Free Survival | Jared Becerril-Rico | 2023 | 0 | 1 | 1 | 0 | 1 | 1 | 1 | 0 | 1 | 0 | 1 | 7 |
| ALL | CD19 | 1 year RFS | Jared Becerril-Rico | 2023 | 0 | 1 | 1 | 0 | 1 | 1 | 1 | 0 | 1 | 0 | 1 | 7 |
| ALL | CD22 | 1 year RFS | Jared Becerril-Rico | 2023 | 0 | 1 | 1 | 0 | 1 | 1 | 1 | 0 | 1 | 0 | 1 | 7 |
| ALL | CD19/CD22 | ≥3 CRS | Jared Becerril-Rico | 2023 | 0 | 1 | 1 | 0 | 1 | 1 | 1 | 0 | 1 | 0 | 1 | 7 |
| ALL | CD19 | ≥3 CRS | Jared Becerril-Rico | 2023 | 0 | 1 | 1 | 0 | 1 | 1 | 1 | 0 | 1 | 0 | 1 | 7 |
| ALL | CD22 | ≥3 CRS | Jared Becerril-Rico | 2023 | 0 | 1 | 1 | 0 | 1 | 1 | 1 | 0 | 1 | 0 | 1 | 7 |
| ALL | CD19/CD22 | Neurotoxicity | Jared Becerril-Rico | 2023 | 0 | 1 | 1 | 0 | 1 | 1 | 1 | 0 | 1 | 0 | 1 | 7 |
| ALL | CD19 | Neurotoxicity | Jared Becerril-Rico | 2023 | 0 | 1 | 1 | 0 | 1 | 1 | 1 | 0 | 1 | 0 | 1 | 7 |
| ALL | CD22 | Neurotoxicity | Jared Becerril-Rico | 2023 | 0 | 1 | 1 | 0 | 1 | 1 | 1 | 0 | 1 | 0 | 1 | 7 |
| R/R B-ALL | CD19 CAR-T | CR | Punita Grover | 2021 | 1 | 1 | 1 | 1 | 1 | 1 | 1 | 0 | 1 | 1 | 1 | 9 |
| R/R B-ALL | CD19 CAR-T | MRD | Punita Grover | 2021 | 1 | 1 | 1 | 1 | 1 | 1 | 1 | 0 | 1 | 1 | 1 | 9 |
| R/R B-ALL | CD19 CAR-T | 1 year PFS | Punita Grover | 2021 | 1 | 1 | 1 | 1 | 1 | 1 | 1 | 0 | 1 | 1 | 1 | 9 |
| R/R B-ALL | CD19 CAR-T | 1 year OS | Punita Grover | 2021 | 1 | 1 | 1 | 1 | 1 | 1 | 1 | 0 | 1 | 1 | 1 | 9 |
| R/R B-ALL | CD19 CAR-T | ≥3 Neurotoxicity | Punita Grover | 2021 | 1 | 1 | 1 | 1 | 1 | 1 | 1 | 0 | 1 | 1 | 1 | 9 |
| R/R B-ALL | CD19 CAR-T | Any Neurotoxicity | Punita Grover | 2021 | 1 | 1 | 1 | 1 | 1 | 1 | 1 | 0 | 1 | 1 | 1 | 9 |
| R/R B-ALL | CD19 CAR-T | ≥3 CRS | Punita Grover | 2021 | 1 | 1 | 1 | 1 | 1 | 1 | 1 | 0 | 1 | 1 | 1 | 9 |
| R/R B-ALL | CD19 CAR-T | Any CRS | Punita Grover | 2021 | 1 | 1 | 1 | 1 | 1 | 1 | 1 | 0 | 1 | 1 | 1 | 9 |
| ALL | CD19 CD28+41BB+EGFRt lentivi32 | optimum response CRS | Kathleen Nagle | 2019 | 0 | 1 | 1 | 0 | 1 | 1 | 1 | 0 | 1 | 0 | 1 | 7 |
| ALL | CD19 CD28+41BB+EGFRt lentivi32 | Neurotoxicity | Kathleen Nagle | 2019 | 0 | 1 | 1 | 0 | 1 | 1 | 1 | 0 | 1 | 0 | 1 | 7 |
| ALL | CD19 CD28+41BB lentivirus | optimum response CRS | Kathleen Nagle | 2019 | 0 | 1 | 1 | 0 | 1 | 1 | 1 | 0 | 1 | 0 | 1 | 7 |
| ALL | CD19 CD28+41BB lentivirus | Neurotoxicity | Kathleen Nagle | 2019 | 0 | 1 | 1 | 0 | 1 | 1 | 1 | 0 | 1 | 0 | 1 | 7 |
| ALL | CD19 CD28 lentivirus | optimum response CRS | Kathleen Nagle | 2019 | 0 | 1 | 1 | 0 | 1 | 1 | 1 | 0 | 1 | 0 | 1 | 7 |
| ALL | CD19 CD28 lentivirus | Neurotoxicity | Kathleen Nagle | 2019 | 0 | 1 | 1 | 0 | 1 | 1 | 1 | 0 | 1 | 0 | 1 | 7 |
| ALL | CD19 CD28+EGFRt lentivirus | optimum response CRS | Kathleen Nagle | 2019 | 0 | 1 | 1 | 0 | 1 | 1 | 1 | 0 | 1 | 0 | 1 | 7 |
| ALL | CD19 CD28+EGFRt lentivirus | Neurotoxicity | Kathleen Nagle | 2019 | 0 | 1 | 1 | 0 | 1 | 1 | 1 | 0 | 1 | 0 | 1 | 7 |
| ALL | CD28 | ITT | Magdi Elsallab | 2023 | 0 | 1 | 1 | 0 | 1 | 1 | 1 | 0 | 1 | 1 | 1 | 9 |
| ALL | 41BB | ITT | Magdi Elsallab | 2023 | 0 | 1 | 1 | 0 | 1 | 1 | 1 | 0 | 1 | 1 | 1 | 9 |
| ALL | Pediatric/Young+adult | ITT | Magdi Elsallab | 2023 | 0 | 1 | 1 | 0 | 1 | 1 | 1 | 0 | 1 | 1 | 1 | 9 |
| ALL | Mixed | ITT | Magdi Elsallab | 2023 | 0 | 1 | 1 | 0 | 1 | 1 | 1 | 0 | 1 | 1 | 1 | 9 |
| ALL | Remission | ITT | Magdi Elsallab | 2023 | 0 | 1 | 1 | 0 | 1 | 1 | 1 | 0 | 1 | 1 | 1 | 9 |
| ALL | disease | ITT | Magdi Elsallab | 2023 | 0 | 1 | 1 | 0 | 1 | 1 | 1 | 0 | 1 | 1 | 1 | 9 |
| ALL | LD-high | ITT | Magdi Elsallab | 2023 | 0 | 1 | 1 | 0 | 1 | 1 | 1 | 0 | 1 | 1 | 1 | 9 |
| ALL | LD-low | ITT | Magdi Elsallab | 2023 | 0 | 1 | 1 | 0 | 1 | 1 | 1 | 0 | 1 | 1 | 1 | 9 |
| ALL | Clinical trial | ITT | Magdi Elsallab | 2023 | 0 | 1 | 1 | 0 | 1 | 1 | 1 | 0 | 1 | 1 | 1 | 9 |
| ALL | real world data | ITT | Magdi Elsallab | 2023 | 0 | 1 | 1 | 0 | 1 | 1 | 1 | 0 | 1 | 1 | 1 | 9 |
| ALL | CD28 | mITT | Magdi Elsallab | 2023 | 0 | 1 | 1 | 0 | 1 | 1 | 1 | 0 | 1 | 1 | 1 | 9 |
| ALL | 41BB | mITT | Magdi Elsallab | 2023 | 0 | 1 | 1 | 0 | 1 | 1 | 1 | 0 | 1 | 1 | 1 | 9 |
| ALL | Pediatric/Young+adult | mITT | Magdi Elsallab | 2023 | 0 | 1 | 1 | 0 | 1 | 1 | 1 | 0 | 1 | 1 | 1 | 9 |
| ALL | Mixed | mITT | Magdi Elsallab | 2023 | 0 | 1 | 1 | 0 | 1 | 1 | 1 | 0 | 1 | 1 | 1 | 9 |
| ALL | Remission | mITT | Magdi Elsallab | 2023 | 0 | 1 | 1 | 0 | 1 | 1 | 1 | 0 | 1 | 1 | 1 | 9 |
| ALL | disease | mITT | Magdi Elsallab | 2023 | 0 | 1 | 1 | 0 | 1 | 1 | 1 | 0 | 1 | 1 | 1 | 9 |
| ALL | LD-high lymphodepletion | mITT | Magdi Elsallab | 2023 | 0 | 1 | 1 | 0 | 1 | 1 | 1 | 0 | 1 | 1 | 1 | 9 |
| ALL | LD-low | mITT | Magdi Elsallab | 2023 | 0 | 1 | 1 | 0 | 1 | 1 | 1 | 0 | 1 | 1 | 1 | 9 |
| ALL | Clinical trial | mITT | Magdi Elsallab | 2023 | 0 | 1 | 1 | 0 | 1 | 1 | 1 | 0 | 1 | 1 | 1 | 9 |
| ALL | real world data | mITT | Magdi Elsallab | 2023 | 0 | 1 | 1 | 0 | 1 | 1 | 1 | 0 | 1 | 1 | 1 | 9 |
| ALL | CD28 | MRD- | Magdi Elsallab | 2023 | 0 | 1 | 1 | 0 | 1 | 1 | 1 | 0 | 1 | 1 | 1 | 9 |
| ALL | 41BB | MRD- | Magdi Elsallab | 2023 | 0 | 1 | 1 | 0 | 1 | 1 | 1 | 0 | 1 | 1 | 1 | 9 |
| ALL | Pediatric/Young+adult | MRD- | Magdi Elsallab | 2023 | 0 | 1 | 1 | 0 | 1 | 1 | 1 | 0 | 1 | 1 | 1 | 9 |
| ALL | Mixed | MRD- | Magdi Elsallab | 2023 | 0 | 1 | 1 | 0 | 1 | 1 | 1 | 0 | 1 | 1 | 1 | 9 |
| ALL | Remission | MRD- | Magdi Elsallab | 2023 | 0 | 1 | 1 | 0 | 1 | 1 | 1 | 0 | 1 | 1 | 1 | 9 |
| ALL | disease | MRD- | Magdi Elsallab | 2023 | 0 | 1 | 1 | 0 | 1 | 1 | 1 | 0 | 1 | 1 | 1 | 9 |
| ALL | LD-high | MRD- | Magdi Elsallab | 2023 | 0 | 1 | 1 | 0 | 1 | 1 | 1 | 0 | 1 | 1 | 1 | 9 |
| ALL | LD-low | MRD- | Magdi Elsallab | 2023 | 0 | 1 | 1 | 0 | 1 | 1 | 1 | 0 | 1 | 1 | 1 | 9 |
| ALL | Clinical trial | MRD- | Magdi Elsallab | 2023 | 0 | 1 | 1 | 0 | 1 | 1 | 1 | 0 | 1 | 1 | 1 | 9 |
| ALL | real world data | MRD- | Magdi Elsallab | 2023 | 0 | 1 | 1 | 0 | 1 | 1 | 1 | 0 | 1 | 1 | 1 | 9 |
| ALL | CD28 | CRS | Magdi Elsallab | 2023 | 0 | 1 | 1 | 0 | 1 | 1 | 1 | 0 | 1 | 1 | 1 | 9 |
| ALL | 41BB | CRS | Magdi Elsallab | 2023 | 0 | 1 | 1 | 0 | 1 | 1 | 1 | 0 | 1 | 1 | 1 | 9 |
| ALL | Pediatric/Young+adult | CRS | Magdi Elsallab | 2023 | 0 | 1 | 1 | 0 | 1 | 1 | 1 | 0 | 1 | 1 | 1 | 9 |
| ALL | Mixed | CRS | Magdi Elsallab | 2023 | 0 | 1 | 1 | 0 | 1 | 1 | 1 | 0 | 1 | 1 | 1 | 9 |
| ALL | Remission | CRS | Magdi Elsallab | 2023 | 0 | 1 | 1 | 0 | 1 | 1 | 1 | 0 | 1 | 1 | 1 | 9 |
| ALL | disease | CRS | Magdi Elsallab | 2023 | 0 | 1 | 1 | 0 | 1 | 1 | 1 | 0 | 1 | 1 | 1 | 9 |
| ALL | LD-high | CRS | Magdi Elsallab | 2023 | 0 | 1 | 1 | 0 | 1 | 1 | 1 | 0 | 1 | 1 | 1 | 9 |
| ALL | LD-low | CRS | Magdi Elsallab | 2023 | 0 | 1 | 1 | 0 | 1 | 1 | 1 | 0 | 1 | 1 | 1 | 9 |
| ALL | Clinical trial | CRS | Magdi Elsallab | 2023 | 0 | 1 | 1 | 0 | 1 | 1 | 1 | 0 | 1 | 1 | 1 | 9 |
| ALL | real world data | CRS | Magdi Elsallab | 2023 | 0 | 1 | 1 | 0 | 1 | 1 | 1 | 0 | 1 | 1 | 1 | 9 |
| ALL | CD28 | ≥3 CRS | Magdi Elsallab | 2023 | 0 | 1 | 1 | 0 | 1 | 1 | 1 | 0 | 1 | 1 | 1 | 9 |
| ALL | 41BB | ≥3 CRS | Magdi Elsallab | 2023 | 0 | 1 | 1 | 0 | 1 | 1 | 1 | 0 | 1 | 1 | 1 | 9 |
| ALL | Pediatric/Young+adult | ≥3 CRS | Magdi Elsallab | 2023 | 0 | 1 | 1 | 0 | 1 | 1 | 1 | 0 | 1 | 1 | 1 | 9 |
| ALL | Mixed | ≥3 CRS | Magdi Elsallab | 2023 | 0 | 1 | 1 | 0 | 1 | 1 | 1 | 0 | 1 | 1 | 1 | 9 |
| ALL | Remission | ≥3 CRS | Magdi Elsallab | 2023 | 0 | 1 | 1 | 0 | 1 | 1 | 1 | 0 | 1 | 1 | 1 | 9 |
| ALL | disease | ≥3 CRS | Magdi Elsallab | 2023 | 0 | 1 | 1 | 0 | 1 | 1 | 1 | 0 | 1 | 1 | 1 | 9 |
| ALL | LD-high lymphodepletion | ≥3 CRS | Magdi Elsallab | 2023 | 0 | 1 | 1 | 0 | 1 | 1 | 1 | 0 | 1 | 1 | 1 | 9 |
| ALL | LD-low | ≥3 CRS | Magdi Elsallab | 2023 | 0 | 1 | 1 | 0 | 1 | 1 | 1 | 0 | 1 | 1 | 1 | 9 |
| ALL | Clinical trial | ≥3 CRS | Magdi Elsallab | 2023 | 0 | 1 | 1 | 0 | 1 | 1 | 1 | 0 | 1 | 1 | 1 | 9 |
| ALL | real world data | ≥3 CRS | Magdi Elsallab | 2023 | 0 | 1 | 1 | 0 | 1 | 1 | 1 | 0 | 1 | 1 | 1 | 9 |
| ALL | CD28 | Neurotoxicity | Magdi Elsallab | 2023 | 0 | 1 | 1 | 0 | 1 | 1 | 1 | 0 | 1 | 1 | 1 | 9 |
| ALL | 41BB | Neurotoxicity | Magdi Elsallab | 2023 | 0 | 1 | 1 | 0 | 1 | 1 | 1 | 0 | 1 | 1 | 1 | 9 |
| ALL | Pediatric/Young+adult | Neurotoxicity | Magdi Elsallab | 2023 | 0 | 1 | 1 | 0 | 1 | 1 | 1 | 0 | 1 | 1 | 1 | 9 |
| ALL | Mixed | Neurotoxicity | Magdi Elsallab | 2023 | 0 | 1 | 1 | 0 | 1 | 1 | 1 | 0 | 1 | 1 | 1 | 9 |
| ALL | Remission | Neurotoxicity | Magdi Elsallab | 2023 | 0 | 1 | 1 | 0 | 1 | 1 | 1 | 0 | 1 | 1 | 1 | 9 |
| ALL | disease | Neurotoxicity | Magdi Elsallab | 2023 | 0 | 1 | 1 | 0 | 1 | 1 | 1 | 0 | 1 | 1 | 1 | 9 |
| ALL | LD-high | Neurotoxicity | Magdi Elsallab | 2023 | 0 | 1 | 1 | 0 | 1 | 1 | 1 | 0 | 1 | 1 | 1 | 9 |
| ALL | LD-low | Neurotoxicity | Magdi Elsallab | 2023 | 0 | 1 | 1 | 0 | 1 | 1 | 1 | 0 | 1 | 1 | 1 | 9 |
| ALL | Clinical trial | Neurotoxicity | Magdi Elsallab | 2023 | 0 | 1 | 1 | 0 | 1 | 1 | 1 | 0 | 1 | 1 | 1 | 9 |
| ALL | real world data | Neurotoxicity | Magdi Elsallab | 2023 | 0 | 1 | 1 | 0 | 1 | 1 | 1 | 0 | 1 | 1 | 1 | 9 |
| ALL | CD19 Lentiviral/41BB allogenic | PFS OS CR AEs PR  GVHD TAE SAE ORR  TtT Nt | Luis Carlos Saiz | 2023 | 1 | 1 | 1 | 0 | 1 | 1 | 1 | 1 | 1 | 1 | 1 | 10 |
| ALL | CD19 Lentiviral/41BB autogenic | PFS OS CR AEs PR  GVHD TAE SAE ORR  TtT Nt | Luis Carlos Saiz | 2023 | 1 | 1 | 1 | 0 | 1 | 1 | 1 | 1 | 1 | 1 | 1 | 10 |
| ALL | CD19 T infused up 10e7 | PFS OS CR AEs PR  GVHD TAE SAE ORR  TtT Nt | Luis Carlos Saiz | 2023 | 1 | 1 | 1 | 0 | 1 | 1 | 1 | 1 | 1 | 1 | 1 | 10 |
| ALL | CD19 T infused down 10e7 | PFS OS CR AEs PR  GVHD TAE SAE ORR  TtT Nt | Luis Carlos Saiz | 2023 | 1 | 1 | 1 | 0 | 1 | 1 | 1 | 1 | 1 | 1 | 1 | 10 |
| ALL | CD19+DLI | PFS OS CR AEs PR  GVHD TAE SAE ORR  TtT Nt | Luis Carlos Saiz | 2023 | 1 | 1 | 1 | 0 | 1 | 1 | 1 | 1 | 1 | 1 | 1 | 10 |
| ALL | CD19+ SOC | PFS OS CR AEs PR  GVHD TAE SAE ORR  TtT Nt | Luis Carlos Saiz | 2023 | 1 | 1 | 1 | 0 | 1 | 1 | 1 | 1 | 1 | 1 | 1 | 10 |
| ALL | CD19+ CH+HSCT | PFS OS CR AEs PR  GVHD TAE SAE ORR  TtT Nt | Luis Carlos Saiz | 2023 | 1 | 1 | 1 | 0 | 1 | 1 | 1 | 1 | 1 | 1 | 1 | 10 |
| ALL | CD19+ polatuzmab | PFS OS CR AEs PR  GVHD TAE SAE ORR  TtT Nt | Luis Carlos Saiz | 2023 | 1 | 1 | 1 | 0 | 1 | 1 | 1 | 1 | 1 | 1 | 1 | 10 |
| ALL | CD19+ chemo | PFS OS CR AEs PR  GVHD TAE SAE ORR  TtT Nt | Luis Carlos Saiz | 2023 | 1 | 1 | 1 | 0 | 1 | 1 | 1 | 1 | 1 | 1 | 1 | 10 |
| ALL | CD19+ ASCT | PFS OS CR AEs PR  GVHD TAE SAE ORR  TtT Nt | Luis Carlos Saiz | 2023 | 1 | 1 | 1 | 0 | 1 | 1 | 1 | 1 | 1 | 1 | 1 | 10 |
| ALL | CD19+ chemo +hsct | PFS OS CR AEs PR  GVHD TAE SAE ORR  TtT Nt | Luis Carlos Saiz | 2023 | 1 | 1 | 1 | 0 | 1 | 1 | 1 | 1 | 1 | 1 | 1 | 10 |
| B-ALL | FMC63-derived | CR | Theodora Anagnostou | 2020 | 0 | 1 | 1 | 0 | 1 | 1 | 0 | 0 | 1 | 0 | 1 | 6 |
| B-ALL | NoFMC63-derived | CR | Theodora Anagnostou | 2020 | 0 | 1 | 1 | 0 | 1 | 1 | 0 | 0 | 1 | 0 | 1 | 6 |
| B-ALL | Auto | CR | Theodora Anagnostou | 2020 | 0 | 1 | 1 | 0 | 1 | 1 | 0 | 0 | 1 | 0 | 1 | 6 |
| B-ALL | Allo | CR | Theodora Anagnostou | 2020 | 0 | 1 | 1 | 0 | 1 | 1 | 0 | 0 | 1 | 0 | 1 | 6 |
| B-ALL | Adult | CR | Theodora Anagnostou | 2020 | 0 | 1 | 1 | 0 | 1 | 1 | 0 | 0 | 1 | 0 | 1 | 6 |
| B-ALL | Young | CR | Theodora Anagnostou | 2020 | 0 | 1 | 1 | 0 | 1 | 1 | 0 | 0 | 1 | 0 | 1 | 6 |
| B-ALL | 41BB | CR | Theodora Anagnostou | 2020 | 0 | 1 | 1 | 0 | 1 | 1 | 0 | 0 | 1 | 0 | 1 | 6 |
| B-ALL | CD28 | CR | Theodora Anagnostou | 2020 | 0 | 1 | 1 | 0 | 1 | 1 | 0 | 0 | 1 | 0 | 1 | 6 |
| B-ALL | 3/4ST | CR | Theodora Anagnostou | 2020 | 0 | 1 | 1 | 0 | 1 | 1 | 0 | 0 | 1 | 0 | 1 | 6 |
| B-ALL | Risk-LOW | CR | Theodora Anagnostou | 2020 | 0 | 1 | 1 | 0 | 1 | 1 | 0 | 0 | 1 | 0 | 1 | 6 |
| B-ALL | Risk-HIGH | CR | Theodora Anagnostou | 2020 | 0 | 1 | 1 | 0 | 1 | 1 | 0 | 0 | 1 | 0 | 1 | 6 |
| B-ALL | FMC63-derived | MRD- | Theodora Anagnostou | 2020 | 0 | 1 | 1 | 0 | 1 | 1 | 0 | 0 | 1 | 0 | 1 | 6 |
| B-ALL | NoFMC63-derived | MRD- | Theodora Anagnostou | 2020 | 0 | 1 | 1 | 0 | 1 | 1 | 0 | 0 | 1 | 0 | 1 | 6 |
| B-ALL | Auto | MRD- | Theodora Anagnostou | 2020 | 0 | 1 | 1 | 0 | 1 | 1 | 0 | 0 | 1 | 0 | 1 | 6 |
| B-ALL | Allo | MRD- | Theodora Anagnostou | 2020 | 0 | 1 | 1 | 0 | 1 | 1 | 0 | 0 | 1 | 0 | 1 | 6 |
| B-ALL | Adult | MRD- | Theodora Anagnostou | 2020 | 0 | 1 | 1 | 0 | 1 | 1 | 0 | 0 | 1 | 0 | 1 | 6 |
| B-ALL | Young | MRD- | Theodora Anagnostou | 2020 | 0 | 1 | 1 | 0 | 1 | 1 | 0 | 0 | 1 | 0 | 1 | 6 |
| B-ALL | 41BB | MRD- | Theodora Anagnostou | 2020 | 0 | 1 | 1 | 0 | 1 | 1 | 0 | 0 | 1 | 0 | 1 | 6 |
| B-ALL | CD28 | MRD- | Theodora Anagnostou | 2020 | 0 | 1 | 1 | 0 | 1 | 1 | 0 | 0 | 1 | 0 | 1 | 6 |
| B-ALL | 3/4ST | MRD- | Theodora Anagnostou | 2020 | 0 | 1 | 1 | 0 | 1 | 1 | 0 | 0 | 1 | 0 | 1 | 6 |
| B-ALL | Risk-LOW | MRD- | Theodora Anagnostou | 2020 | 0 | 1 | 1 | 0 | 1 | 1 | 0 | 0 | 1 | 0 | 1 | 6 |
| B-ALL | Risk-HIGH | MRD- | Theodora Anagnostou | 2020 | 0 | 1 | 1 | 0 | 1 | 1 | 0 | 0 | 1 | 0 | 1 | 6 |
| B-ALL | FMC63-derived | 1 year OS | Theodora Anagnostou | 2020 | 0 | 1 | 1 | 0 | 1 | 1 | 0 | 0 | 1 | 0 | 1 | 6 |
| B-ALL | NoFMC63-derived | 1 year OS | Theodora Anagnostou | 2020 | 0 | 1 | 1 | 0 | 1 | 1 | 0 | 0 | 1 | 0 | 1 | 6 |
| B-ALL | Auto | 1 year OS | Theodora Anagnostou | 2020 | 0 | 1 | 1 | 0 | 1 | 1 | 0 | 0 | 1 | 0 | 1 | 6 |
| B-ALL | Allo | 1 year OS | Theodora Anagnostou | 2020 | 0 | 1 | 1 | 0 | 1 | 1 | 0 | 0 | 1 | 0 | 1 | 6 |
| B-ALL | Adult | 1 year OS | Theodora Anagnostou | 2020 | 0 | 1 | 1 | 0 | 1 | 1 | 0 | 0 | 1 | 0 | 1 | 6 |
| B-ALL | Young | 1 year OS | Theodora Anagnostou | 2020 | 0 | 1 | 1 | 0 | 1 | 1 | 0 | 0 | 1 | 0 | 1 | 6 |
| B-ALL | 41BB | 1 year OS | Theodora Anagnostou | 2020 | 0 | 1 | 1 | 0 | 1 | 1 | 0 | 0 | 1 | 0 | 1 | 6 |
| B-ALL | CD28 | 1 year OS | Theodora Anagnostou | 2020 | 0 | 1 | 1 | 0 | 1 | 1 | 0 | 0 | 1 | 0 | 1 | 6 |
| B-ALL | 3/4ST | 1 year OS | Theodora Anagnostou | 2020 | 0 | 1 | 1 | 0 | 1 | 1 | 0 | 0 | 1 | 0 | 1 | 6 |
| B-ALL | Risk-LOW | 1 year OS | Theodora Anagnostou | 2020 | 0 | 1 | 1 | 0 | 1 | 1 | 0 | 0 | 1 | 0 | 1 | 6 |
| B-ALL | Risk-HIGH | 1 year OS | Theodora Anagnostou | 2020 | 0 | 1 | 1 | 0 | 1 | 1 | 0 | 0 | 1 | 0 | 1 | 6 |
| B-ALL | FMC63-derived | 1 year PFS | Theodora Anagnostou | 2020 | 0 | 1 | 1 | 0 | 1 | 1 | 0 | 0 | 1 | 0 | 1 | 6 |
| B-ALL | NoFMC63-derived | 1 year PFS | Theodora Anagnostou | 2020 | 0 | 1 | 1 | 0 | 1 | 1 | 0 | 0 | 1 | 0 | 1 | 6 |
| B-ALL | Auto | 1 year PFS | Theodora Anagnostou | 2020 | 0 | 1 | 1 | 0 | 1 | 1 | 0 | 0 | 1 | 0 | 1 | 6 |
| B-ALL | Allo | 1 year PFS | Theodora Anagnostou | 2020 | 0 | 1 | 1 | 0 | 1 | 1 | 0 | 0 | 1 | 0 | 1 | 6 |
| B-ALL | Adult | 1 year PFS | Theodora Anagnostou | 2020 | 0 | 1 | 1 | 0 | 1 | 1 | 0 | 0 | 1 | 0 | 1 | 6 |
| B-ALL | Young | 1 year PFS | Theodora Anagnostou | 2020 | 0 | 1 | 1 | 0 | 1 | 1 | 0 | 0 | 1 | 0 | 1 | 6 |
| B-ALL | 41BB | 1 year PFS | Theodora Anagnostou | 2020 | 0 | 1 | 1 | 0 | 1 | 1 | 0 | 0 | 1 | 0 | 1 | 6 |
| B-ALL | CD28 | 1 year PFS | Theodora Anagnostou | 2020 | 0 | 1 | 1 | 0 | 1 | 1 | 0 | 0 | 1 | 0 | 1 | 6 |
| B-ALL | 3/4ST | 1 year PFS | Theodora Anagnostou | 2020 | 0 | 1 | 1 | 0 | 1 | 1 | 0 | 0 | 1 | 0 | 1 | 6 |
| B-ALL | Risk-LOW | 1 year PFS | Theodora Anagnostou | 2020 | 0 | 1 | 1 | 0 | 1 | 1 | 0 | 0 | 1 | 0 | 1 | 6 |
| B-ALL | Risk-HIGH | 1 year PFS | Theodora Anagnostou | 2020 | 0 | 1 | 1 | 0 | 1 | 1 | 0 | 0 | 1 | 0 | 1 | 6 |
| B-ALL | FMC63-derived | CRS | Theodora Anagnostou | 2020 | 0 | 1 | 1 | 0 | 1 | 1 | 0 | 0 | 1 | 0 | 1 | 6 |
| B-ALL | NoFMC63-derived | CRS | Theodora Anagnostou | 2020 | 0 | 1 | 1 | 0 | 1 | 1 | 0 | 0 | 1 | 0 | 1 | 6 |
| B-ALL | Auto | CRS | Theodora Anagnostou | 2020 | 0 | 1 | 1 | 0 | 1 | 1 | 0 | 0 | 1 | 0 | 1 | 6 |
| B-ALL | Allo | CRS | Theodora Anagnostou | 2020 | 0 | 1 | 1 | 0 | 1 | 1 | 0 | 0 | 1 | 0 | 1 | 6 |
| B-ALL | Adult | CRS | Theodora Anagnostou | 2020 | 0 | 1 | 1 | 0 | 1 | 1 | 0 | 0 | 1 | 0 | 1 | 6 |
| B-ALL | Young | CRS | Theodora Anagnostou | 2020 | 0 | 1 | 1 | 0 | 1 | 1 | 0 | 0 | 1 | 0 | 1 | 6 |
| B-ALL | 41BB | CRS | Theodora Anagnostou | 2020 | 0 | 1 | 1 | 0 | 1 | 1 | 0 | 0 | 1 | 0 | 1 | 6 |
| B-ALL | CD28 | CRS | Theodora Anagnostou | 2020 | 0 | 1 | 1 | 0 | 1 | 1 | 0 | 0 | 1 | 0 | 1 | 6 |
| B-ALL | 3/4ST | CRS | Theodora Anagnostou | 2020 | 0 | 1 | 1 | 0 | 1 | 1 | 0 | 0 | 1 | 0 | 1 | 6 |
| B-ALL | Risk-LOW | CRS | Theodora Anagnostou | 2020 | 0 | 1 | 1 | 0 | 1 | 1 | 0 | 0 | 1 | 0 | 1 | 6 |
| B-ALL | Risk-HIGH | CRS | Theodora Anagnostou | 2020 | 0 | 1 | 1 | 0 | 1 | 1 | 0 | 0 | 1 | 0 | 1 | 6 |
| B-ALL | FMC63-derived | 3-4 CRS | Theodora Anagnostou | 2020 | 0 | 1 | 1 | 0 | 1 | 1 | 0 | 0 | 1 | 0 | 1 | 6 |
| B-ALL | NoFMC63-derived | 3-4 CRS | Theodora Anagnostou | 2020 | 0 | 1 | 1 | 0 | 1 | 1 | 0 | 0 | 1 | 0 | 1 | 6 |
| B-ALL | Auto | 3-4 CRS | Theodora Anagnostou | 2020 | 0 | 1 | 1 | 0 | 1 | 1 | 0 | 0 | 1 | 0 | 1 | 6 |
| B-ALL | Allo | 3-4 CRS | Theodora Anagnostou | 2020 | 0 | 1 | 1 | 0 | 1 | 1 | 0 | 0 | 1 | 0 | 1 | 6 |
| B-ALL | Adult | 3-4 CRS | Theodora Anagnostou | 2020 | 0 | 1 | 1 | 0 | 1 | 1 | 0 | 0 | 1 | 0 | 1 | 6 |
| B-ALL | Young | 3-4 CRS | Theodora Anagnostou | 2020 | 0 | 1 | 1 | 0 | 1 | 1 | 0 | 0 | 1 | 0 | 1 | 6 |
| B-ALL | 41BB | 3-4 CRS | Theodora Anagnostou | 2020 | 0 | 1 | 1 | 0 | 1 | 1 | 0 | 0 | 1 | 0 | 1 | 6 |
| B-ALL | CD28 | 3-4 CRS | Theodora Anagnostou | 2020 | 0 | 1 | 1 | 0 | 1 | 1 | 0 | 0 | 1 | 0 | 1 | 6 |
| B-ALL | 3/4ST | 3-4 CRS | Theodora Anagnostou | 2020 | 0 | 1 | 1 | 0 | 1 | 1 | 0 | 0 | 1 | 0 | 1 | 6 |
| B-ALL | Risk-LOW | 3-4 CRS | Theodora Anagnostou | 2020 | 0 | 1 | 1 | 0 | 1 | 1 | 0 | 0 | 1 | 0 | 1 | 6 |
| B-ALL | Risk-HIGH | 3-4 CRS | Theodora Anagnostou | 2020 | 0 | 1 | 1 | 0 | 1 | 1 | 0 | 0 | 1 | 0 | 1 | 6 |
| B-ALL | FMC63-derived | Neurotoxicity | Theodora Anagnostou | 2020 | 0 | 1 | 1 | 0 | 1 | 1 | 0 | 0 | 1 | 0 | 1 | 6 |
| B-ALL | NoFMC63-derived | Neurotoxicity | Theodora Anagnostou | 2020 | 0 | 1 | 1 | 0 | 1 | 1 | 0 | 0 | 1 | 0 | 1 | 6 |
| B-ALL | Auto | Neurotoxicity | Theodora Anagnostou | 2020 | 0 | 1 | 1 | 0 | 1 | 1 | 0 | 0 | 1 | 0 | 1 | 6 |
| B-ALL | Allo | Neurotoxicity | Theodora Anagnostou | 2020 | 0 | 1 | 1 | 0 | 1 | 1 | 0 | 0 | 1 | 0 | 1 | 6 |
| B-ALL | Adult | Neurotoxicity | Theodora Anagnostou | 2020 | 0 | 1 | 1 | 0 | 1 | 1 | 0 | 0 | 1 | 0 | 1 | 6 |
| B-ALL | Young | Neurotoxicity | Theodora Anagnostou | 2020 | 0 | 1 | 1 | 0 | 1 | 1 | 0 | 0 | 1 | 0 | 1 | 6 |
| B-ALL | 41BB | Neurotoxicity | Theodora Anagnostou | 2020 | 0 | 1 | 1 | 0 | 1 | 1 | 0 | 0 | 1 | 0 | 1 | 6 |
| B-ALL | CD28 | Neurotoxicity | Theodora Anagnostou | 2020 | 0 | 1 | 1 | 0 | 1 | 1 | 0 | 0 | 1 | 0 | 1 | 6 |
| B-ALL | 3/4ST | Neurotoxicity | Theodora Anagnostou | 2020 | 0 | 1 | 1 | 0 | 1 | 1 | 0 | 0 | 1 | 0 | 1 | 6 |
| B-ALL | Risk-LOW | Neurotoxicity | Theodora Anagnostou | 2020 | 0 | 1 | 1 | 0 | 1 | 1 | 0 | 0 | 1 | 0 | 1 | 6 |
| B-ALL | Risk-HIGH | Neurotoxicity | Theodora Anagnostou | 2020 | 0 | 1 | 1 | 0 | 1 | 1 | 0 | 0 | 1 | 0 | 1 | 6 |
| B-ALL | FMC63-derived | 3-4 Neurotoxicity | Theodora Anagnostou | 2020 | 0 | 1 | 1 | 0 | 1 | 1 | 0 | 0 | 1 | 0 | 1 | 6 |
| B-ALL | NoFMC63-derived | 3-4 Neurotoxicity | Theodora Anagnostou | 2020 | 0 | 1 | 1 | 0 | 1 | 1 | 0 | 0 | 1 | 0 | 1 | 6 |
| B-ALL | Auto | 3-4 Neurotoxicity | Theodora Anagnostou | 2020 | 0 | 1 | 1 | 0 | 1 | 1 | 0 | 0 | 1 | 0 | 1 | 6 |
| B-ALL | Allo | 3-4 Neurotoxicity | Theodora Anagnostou | 2020 | 0 | 1 | 1 | 0 | 1 | 1 | 0 | 0 | 1 | 0 | 1 | 6 |
| B-ALL | Adult | 3-4 Neurotoxicity | Theodora Anagnostou | 2020 | 0 | 1 | 1 | 0 | 1 | 1 | 0 | 0 | 1 | 0 | 1 | 6 |
| B-ALL | Young | 3-4 Neurotoxicity | Theodora Anagnostou | 2020 | 0 | 1 | 1 | 0 | 1 | 1 | 0 | 0 | 1 | 0 | 1 | 6 |
| B-ALL | 41BB | 3-4 Neurotoxicity | Theodora Anagnostou | 2020 | 0 | 1 | 1 | 0 | 1 | 1 | 0 | 0 | 1 | 0 | 1 | 6 |
| B-ALL | CD28 | 3-4 Neurotoxicity | Theodora Anagnostou | 2020 | 0 | 1 | 1 | 0 | 1 | 1 | 0 | 0 | 1 | 0 | 1 | 6 |
| B-ALL | 3/4ST | 3-4 Neurotoxicity | Theodora Anagnostou | 2020 | 0 | 1 | 1 | 0 | 1 | 1 | 0 | 0 | 1 | 0 | 1 | 6 |
| B-ALL | Risk-LOW | 3-4 Neurotoxicity | Theodora Anagnostou | 2020 | 0 | 1 | 1 | 0 | 1 | 1 | 0 | 0 | 1 | 0 | 1 | 6 |
| B-ALL | Risk-HIGH | 3-4 Neurotoxicity | Theodora Anagnostou | 2020 | 0 | 1 | 1 | 0 | 1 | 1 | 0 | 0 | 1 | 0 | 1 | 6 |
| RR ALL | blinatumomab | CR | Yixin Zhai | 2024 | 0 | 1 | 1 | 0 | 1 | 1 | 1 | 0 | 1 | 1 | 1 | 8 |
| RR ALL | CD19 CAR-T | CR | Yixin Zhai | 2024 | 0 | 1 | 1 | 0 | 1 | 1 | 1 | 0 | 1 | 1 | 1 | 8 |
| RR ALL | blinatumomab | MRD- | Yixin Zhai | 2024 | 0 | 1 | 1 | 0 | 1 | 1 | 1 | 0 | 1 | 1 | 1 | 8 |
| RR ALL | CD19 CAR-T | MRD- | Yixin Zhai | 2024 | 0 | 1 | 1 | 0 | 1 | 1 | 1 | 0 | 1 | 1 | 1 | 8 |
| RR ALL | Blinatumomab+Allo-SCT | 3-4 CRS | Yixin Zhai | 2024 | 0 | 1 | 1 | 0 | 1 | 1 | 1 | 0 | 1 | 1 | 1 | 8 |
| RR ALL | CD19 CAR-T+Allo-SCT | 3-4 CRS | Yixin Zhai | 2024 | 0 | 1 | 1 | 0 | 1 | 1 | 1 | 0 | 1 | 1 | 1 | 8 |
| RR ALL | blinatumomab | 3-4 neurotoxicity | Yixin Zhai | 2024 | 0 | 1 | 1 | 0 | 1 | 1 | 1 | 0 | 1 | 1 | 1 | 8 |
| RR ALL | CD19 CAR-T | 3-4 neurotoxicity | Yixin Zhai | 2024 | 0 | 1 | 1 | 0 | 1 | 1 | 1 | 0 | 1 | 1 | 1 | 8 |
| RR ALL | Blinatumomab+Allo-SCT | 3-4 aGVHD | Yixin Zhai | 2024 | 0 | 1 | 1 | 0 | 1 | 1 | 1 | 0 | 1 | 1 | 1 | 8 |
| RR ALL | CD19 CAR-T+Allo-SCT | 3-4 aGVHD | Yixin Zhai | 2024 | 0 | 1 | 1 | 0 | 1 | 1 | 1 | 0 | 1 | 1 | 1 | 8 |
| RR ALL | Blinatumomab+Allo-SCT | 2 year cGVHD | Yixin Zhai | 2024 | 0 | 1 | 1 | 0 | 1 | 1 | 1 | 0 | 1 | 1 | 1 | 8 |
| RR ALL | CD19 CAR-T+Allo-SCT | 2 year cGVHD | Yixin Zhai | 2024 | 0 | 1 | 1 | 0 | 1 | 1 | 1 | 0 | 1 | 1 | 1 | 8 |
| RR B-ALL | CD19 | OR CR OS | Han‐Yu Cao | 2023 | 0 | 1 | 1 | 0 | 1 | 1 | 0 | 0 | 1 | 1 | 1 | 7 |
| RR B-ALL | CD19/CD22 | OR CR OS | Han‐Yu Cao | 2023 | 0 | 1 | 1 | 0 | 1 | 1 | 0 | 0 | 1 | 1 | 1 | 7 |
| RR B-ALL | Sequential CD19-22 vsInotuzumab Ozogamicin | OR CR OS | Han‐Yu Cao | 2023 | 0 | 1 | 1 | 0 | 1 | 1 | 0 | 0 | 1 | 1 | 1 | 7 |
| RR B-ALL | Blinatumomab | OR CR OS | Han‐Yu Cao | 2023 | 0 | 1 | 1 | 0 | 1 | 1 | 0 | 0 | 1 | 1 | 1 | 7 |
| RR B-ALL | Standard Chemotherapy | OR CR OS | Han‐Yu Cao | 2023 | 0 | 1 | 1 | 0 | 1 | 1 | 0 | 0 | 1 | 1 | 1 | 7 |
| MM | BsAb | Any CRS | Pooneh Soltantabar | 2024 | 0 | 1 | 1 | 0 | 1 | 1 | 1 | 0 | 1 | 1 | 1 | 8 |
| MM | BsAb | ≥3 CRS | Pooneh Soltantabar | 2024 | 0 | 1 | 1 | 0 | 1 | 1 | 1 | 0 | 1 | 1 | 1 | 8 |
| MM | CAR-T | Any CRS | Pooneh Soltantabar | 2024 | 0 | 1 | 1 | 0 | 1 | 1 | 1 | 0 | 1 | 1 | 1 | 8 |
| MM | CAR-T | ≥3 CRS | Pooneh Soltantabar | 2024 | 0 | 1 | 1 | 0 | 1 | 1 | 1 | 0 | 1 | 1 | 1 | 8 |
| MM | Intravenous | Any CRS | Pooneh Soltantabar | 2024 | 0 | 1 | 1 | 0 | 1 | 1 | 1 | 0 | 1 | 1 | 1 | 8 |
| MM | Intravenous | ≥3 CRS | Pooneh Soltantabar | 2024 | 0 | 1 | 1 | 0 | 1 | 1 | 1 | 0 | 1 | 1 | 1 | 8 |
| MM | Subcutaneous | Any CRS | Pooneh Soltantabar | 2024 | 0 | 1 | 1 | 0 | 1 | 1 | 1 | 0 | 1 | 1 | 1 | 8 |
| MM | Subcutaneous | ≥3 CRS | Pooneh Soltantabar | 2024 | 0 | 1 | 1 | 0 | 1 | 1 | 1 | 0 | 1 | 1 | 1 | 8 |
| RRMM | BCMA | CR | Jingjing Li | 2022 | 0 | 1 | 1 | 0 | 1 | 1 | 1 | 0 | 1 | 1 | 1 | 8 |
| RRMM | LCAR-B38M | CR | Jingjing Li | 2022 | 0 | 1 | 1 | 0 | 1 | 1 | 1 | 0 | 1 | 1 | 1 | 8 |
| RRMM | BCMA+CD19 | CR | Jingjing Li | 2022 | 0 | 1 | 1 | 0 | 1 | 1 | 1 | 0 | 1 | 1 | 1 | 8 |
| RRMM | 41BB | CR | Jingjing Li | 2022 | 0 | 1 | 1 | 0 | 1 | 1 | 1 | 0 | 1 | 1 | 1 | 8 |
| RRMM | CD28 | CR | Jingjing Li | 2022 | 0 | 1 | 1 | 0 | 1 | 1 | 1 | 0 | 1 | 1 | 1 | 8 |
| RRMM | CD3+CD28 | CR | Jingjing Li | 2022 | 0 | 1 | 1 | 0 | 1 | 1 | 1 | 0 | 1 | 1 | 1 | 8 |
| RRMM | CD3 | CR | Jingjing Li | 2022 | 0 | 1 | 1 | 0 | 1 | 1 | 1 | 0 | 1 | 1 | 1 | 8 |
| RRMM | BCMA | CRS | Jingjing Li | 2022 | 0 | 1 | 1 | 0 | 1 | 1 | 1 | 0 | 1 | 1 | 1 | 8 |
| RRMM | LCAR-B38M | CRS | Jingjing Li | 2022 | 0 | 1 | 1 | 0 | 1 | 1 | 1 | 0 | 1 | 1 | 1 | 8 |
| RRMM | BCMA+CD19 | CRS | Jingjing Li | 2022 | 0 | 1 | 1 | 0 | 1 | 1 | 1 | 0 | 1 | 1 | 1 | 8 |
| RRMM | 41BB | CRS | Jingjing Li | 2022 | 0 | 1 | 1 | 0 | 1 | 1 | 1 | 0 | 1 | 1 | 1 | 8 |
| RRMM | CD28 | CRS | Jingjing Li | 2022 | 0 | 1 | 1 | 0 | 1 | 1 | 1 | 0 | 1 | 1 | 1 | 8 |
| RRMM | CD3 | CRS | Jingjing Li | 2022 | 0 | 1 | 1 | 0 | 1 | 1 | 1 | 0 | 1 | 1 | 1 | 8 |
| RRMM | CAR-T | AE | Jingjing Li | 2022 | 0 | 1 | 1 | 0 | 1 | 1 | 1 | 0 | 1 | 1 | 1 | 8 |
| B-ALL | CD19 CAR-T | CRS | Jin | 2018 | 0 | 1 | 1 | 0 | 1 | 1 | 1 | 0 | 1 | 1 | 1 | 8 |
| B-CLL | CD19 CAR-T | CRS | Jin | 2018 | 0 | 1 | 1 | 0 | 1 | 1 | 1 | 0 | 1 | 1 | 1 | 8 |
| B-NHL | CD19 CAR-T | CRS | Jin | 2018 | 0 | 1 | 1 | 0 | 1 | 1 | 1 | 0 | 1 | 1 | 1 | 8 |
| CLL+ B-NHL+ALL | Post-HSCT | CRS | Jin | 2018 | 0 | 1 | 1 | 0 | 1 | 1 | 1 | 0 | 1 | 1 | 1 | 8 |
| CLL+ B-NHL+ALL | NO-HSCT | CRS | Jin | 2018 | 0 | 1 | 1 | 0 | 1 | 1 | 1 | 0 | 1 | 1 | 1 | 8 |
| RRMM | Eastern | OR | Qin Yang | 2021 | 0 | 1 | 1 | 0 | 1 | 1 | 1 | 0 | 1 | 1 | 1 | 8 |
| RRMM | Western | OR | Qin Yang | 2021 | 0 | 1 | 1 | 0 | 1 | 1 | 1 | 0 | 1 | 1 | 1 | 8 |
| RRMM | Single center | OR | Qin Yang | 2021 | 0 | 1 | 1 | 0 | 1 | 1 | 1 | 0 | 1 | 1 | 1 | 8 |
| RRMM | mutiple center | OR | Qin Yang | 2021 | 0 | 1 | 1 | 0 | 1 | 1 | 1 | 0 | 1 | 1 | 1 | 8 |
| RRMM | Status completed | OR | Qin Yang | 2021 | 0 | 1 | 1 | 0 | 1 | 1 | 1 | 0 | 1 | 1 | 1 | 8 |
| RRMM | Status ongoning | OR | Qin Yang | 2021 | 0 | 1 | 1 | 0 | 1 | 1 | 1 | 0 | 1 | 1 | 1 | 8 |
| RRMM | Human scfv | OR | Qin Yang | 2021 | 0 | 1 | 1 | 0 | 1 | 1 | 1 | 0 | 1 | 1 | 1 | 8 |
| RRMM | No Human scfv | OR | Qin Yang | 2021 | 0 | 1 | 1 | 0 | 1 | 1 | 1 | 0 | 1 | 1 | 1 | 8 |
| RRMM | 41bb | OR | Qin Yang | 2021 | 0 | 1 | 1 | 0 | 1 | 1 | 1 | 0 | 1 | 1 | 1 | 8 |
| RRMM | CD28 | OR | Qin Yang | 2021 | 0 | 1 | 1 | 0 | 1 | 1 | 1 | 0 | 1 | 1 | 1 | 8 |
| RRMM | 2^nd^ generation | OR | Qin Yang | 2021 | 0 | 1 | 1 | 0 | 1 | 1 | 1 | 0 | 1 | 1 | 1 | 8 |
| RRMM | 3^rd^ generation | OR | Qin Yang | 2021 | 0 | 1 | 1 | 0 | 1 | 1 | 1 | 0 | 1 | 1 | 1 | 8 |
| RRMM | BCMA | OR | Qin Yang | 2021 | 0 | 1 | 1 | 0 | 1 | 1 | 1 | 0 | 1 | 1 | 1 | 8 |
| RRMM | NO BCMA | OR | Qin Yang | 2021 | 0 | 1 | 1 | 0 | 1 | 1 | 1 | 0 | 1 | 1 | 1 | 8 |
| RRMM | BCMA contained | OR | Qin Yang | 2021 | 0 | 1 | 1 | 0 | 1 | 1 | 1 | 0 | 1 | 1 | 1 | 8 |
| RRMM | BCMA uncontained | OR | Qin Yang | 2021 | 0 | 1 | 1 | 0 | 1 | 1 | 1 | 0 | 1 | 1 | 1 | 8 |
| RRMM | Single-target | OR | Qin Yang | 2021 | 0 | 1 | 1 | 0 | 1 | 1 | 1 | 0 | 1 | 1 | 1 | 8 |
| RRMM | Dual -target | OR | Qin Yang | 2021 | 0 | 1 | 1 | 0 | 1 | 1 | 1 | 0 | 1 | 1 | 1 | 8 |
| RRMM | Eastern | CRS | Qin Yang | 2021 | 0 | 1 | 1 | 0 | 1 | 1 | 1 | 0 | 1 | 1 | 1 | 8 |
| RRMM | Western | CRS | Qin Yang | 2021 | 0 | 1 | 1 | 0 | 1 | 1 | 1 | 0 | 1 | 1 | 1 | 8 |
| RRMM | Single center | CRS | Qin Yang | 2021 | 0 | 1 | 1 | 0 | 1 | 1 | 1 | 0 | 1 | 1 | 1 | 8 |
| RRMM | mutiple center | CRS | Qin Yang | 2021 | 0 | 1 | 1 | 0 | 1 | 1 | 1 | 0 | 1 | 1 | 1 | 8 |
| RRMM | Status completed | CRS | Qin Yang | 2021 | 0 | 1 | 1 | 0 | 1 | 1 | 1 | 0 | 1 | 1 | 1 | 8 |
| RRMM | Status ongoning | CRS | Qin Yang | 2021 | 0 | 1 | 1 | 0 | 1 | 1 | 1 | 0 | 1 | 1 | 1 | 8 |
| RRMM | Human scfv | CRS | Qin Yang | 2021 | 0 | 1 | 1 | 0 | 1 | 1 | 1 | 0 | 1 | 1 | 1 | 8 |
| RRMM | No Human scfv | CRS | Qin Yang | 2021 | 0 | 1 | 1 | 0 | 1 | 1 | 1 | 0 | 1 | 1 | 1 | 8 |
| RRMM | 41bb | CRS | Qin Yang | 2021 | 0 | 1 | 1 | 0 | 1 | 1 | 1 | 0 | 1 | 1 | 1 | 8 |
| RRMM | CD28 | CRS | Qin Yang | 2021 | 0 | 1 | 1 | 0 | 1 | 1 | 1 | 0 | 1 | 1 | 1 | 8 |
| RRMM | 2^nd^ generation | CRS | Qin Yang | 2021 | 0 | 1 | 1 | 0 | 1 | 1 | 1 | 0 | 1 | 1 | 1 | 8 |
| RRMM | 3^rd^ generation | CRS | Qin Yang | 2021 | 0 | 1 | 1 | 0 | 1 | 1 | 1 | 0 | 1 | 1 | 1 | 8 |
| RRMM | BCMA | CRS | Qin Yang | 2021 | 0 | 1 | 1 | 0 | 1 | 1 | 1 | 0 | 1 | 1 | 1 | 8 |
| RRMM | NO BCMA | CRS | Qin Yang | 2021 | 0 | 1 | 1 | 0 | 1 | 1 | 1 | 0 | 1 | 1 | 1 | 8 |
| RRMM | BCMA contained | CRS | Qin Yang | 2021 | 0 | 1 | 1 | 0 | 1 | 1 | 1 | 0 | 1 | 1 | 1 | 8 |
| RRMM | BCMA uncontained | CRS | Qin Yang | 2021 | 0 | 1 | 1 | 0 | 1 | 1 | 1 | 0 | 1 | 1 | 1 | 8 |
| RRMM | Single-target | CRS | Qin Yang | 2021 | 0 | 1 | 1 | 0 | 1 | 1 | 1 | 0 | 1 | 1 | 1 | 8 |
| RRMM | Dual -target | CRS | Qin Yang | 2021 | 0 | 1 | 1 | 0 | 1 | 1 | 1 | 0 | 1 | 1 | 1 | 8 |
| RRMM | Eastern | Neurotoxicity | Qin Yang | 2021 | 0 | 1 | 1 | 0 | 1 | 1 | 1 | 0 | 1 | 1 | 1 | 8 |
| RRMM | Western | Neurotoxicity | Qin Yang | 2021 | 0 | 1 | 1 | 0 | 1 | 1 | 1 | 0 | 1 | 1 | 1 | 8 |
| RRMM | Single center | Neurotoxicity | Qin Yang | 2021 | 0 | 1 | 1 | 0 | 1 | 1 | 1 | 0 | 1 | 1 | 1 | 8 |
| RRMM | mutiple center | Neurotoxicity | Qin Yang | 2021 | 0 | 1 | 1 | 0 | 1 | 1 | 1 | 0 | 1 | 1 | 1 | 8 |
| RRMM | Status completed | Neurotoxicity | Qin Yang | 2021 | 0 | 1 | 1 | 0 | 1 | 1 | 1 | 0 | 1 | 1 | 1 | 8 |
| RRMM | Status ongoning | Neurotoxicity | Qin Yang | 2021 | 0 | 1 | 1 | 0 | 1 | 1 | 1 | 0 | 1 | 1 | 1 | 8 |
| RRMM | Human scfv | Neurotoxicity | Qin Yang | 2021 | 0 | 1 | 1 | 0 | 1 | 1 | 1 | 0 | 1 | 1 | 1 | 8 |
| RRMM | No Human scfv | Neurotoxicity | Qin Yang | 2021 | 0 | 1 | 1 | 0 | 1 | 1 | 1 | 0 | 1 | 1 | 1 | 8 |
| RRMM | 41bb | Neurotoxicity | Qin Yang | 2021 | 0 | 1 | 1 | 0 | 1 | 1 | 1 | 0 | 1 | 1 | 1 | 8 |
| RRMM | CD28 | Neurotoxicity | Qin Yang | 2021 | 0 | 1 | 1 | 0 | 1 | 1 | 1 | 0 | 1 | 1 | 1 | 8 |
| RRMM | 2^nd^ generation | Neurotoxicity | Qin Yang | 2021 | 0 | 1 | 1 | 0 | 1 | 1 | 1 | 0 | 1 | 1 | 1 | 8 |
| RRMM | 3^rd^ generation | Neurotoxicity | Qin Yang | 2021 | 0 | 1 | 1 | 0 | 1 | 1 | 1 | 0 | 1 | 1 | 1 | 8 |
| RRMM | BCMA | Neurotoxicity | Qin Yang | 2021 | 0 | 1 | 1 | 0 | 1 | 1 | 1 | 0 | 1 | 1 | 1 | 8 |
| RRMM | NO BCMA | Neurotoxicity | Qin Yang | 2021 | 0 | 1 | 1 | 0 | 1 | 1 | 1 | 0 | 1 | 1 | 1 | 8 |
| RRMM | BCMA contained | Neurotoxicity | Qin Yang | 2021 | 0 | 1 | 1 | 0 | 1 | 1 | 1 | 0 | 1 | 1 | 1 | 8 |
| RRMM | BCMA uncontained | Neurotoxicity | Qin Yang | 2021 | 0 | 1 | 1 | 0 | 1 | 1 | 1 | 0 | 1 | 1 | 1 | 8 |
| RRMM | Single-target | Neurotoxicity | Qin Yang | 2021 | 0 | 1 | 1 | 0 | 1 | 1 | 1 | 0 | 1 | 1 | 1 | 8 |
| RRMM | Dual -target | Neurotoxicity | Qin Yang | 2021 | 0 | 1 | 1 | 0 | 1 | 1 | 1 | 0 | 1 | 1 | 1 | 8 |
| MM | no ASCT+BCMA CART | MRD- CRS OR OS  NT CR | Jia Zhang | 2024 | 0 | 1 | 1 | 0 | 1 | 1 | 1 | 0 | 1 | 1 | 1 | 8 |
| MM | ASCT+BCMA CART | MRD- CRS OR OS  NT CR | Jia Zhang | 2024 | 0 | 1 | 1 | 0 | 1 | 1 | 1 | 0 | 1 | 1 | 1 | 8 |
| MM | NKG2D | MRD- CRS OR OS  NT CR | Jia Zhang | 2024 | 0 | 1 | 1 | 0 | 1 | 1 | 1 | 0 | 1 | 1 | 1 | 8 |
| MM | BCMA | MRD- CRS OR OS  NT CR | Jia Zhang | 2024 | 0 | 1 | 1 | 0 | 1 | 1 | 1 | 0 | 1 | 1 | 1 | 8 |
| MM | k light chain | MRD- CRS OR OS  NT CR | Jia Zhang | 2024 | 0 | 1 | 1 | 0 | 1 | 1 | 1 | 0 | 1 | 1 | 1 | 8 |
| MM | BCMA+CD19 Lentiviral/4–1BB/ human | MRD- CRS OR OS  NT CR | Jia Zhang | 2024 | 0 | 1 | 1 | 0 | 1 | 1 | 1 | 0 | 1 | 1 | 1 | 8 |
| MM | BCMA+CD38 Lentiviral/4–1BB/ human | MRD- CRS OR OS  NT CR | Jia Zhang | 2024 | 0 | 1 | 1 | 0 | 1 | 1 | 1 | 0 | 1 | 1 | 1 | 8 |
| MM | BCMA lymphodeletion melphalan | MRD- CRS OR OS  NT CR | Jia Zhang | 2024 | 0 | 1 | 1 | 0 | 1 | 1 | 1 | 0 | 1 | 1 | 1 | 8 |
| MM | BCMA lymphodeletion flu/cy | MRD- CRS OR OS  NT CR | Jia Zhang | 2024 | 0 | 1 | 1 | 0 | 1 | 1 | 1 | 0 | 1 | 1 | 1 | 8 |
| MM | BCMA lymphodeletion cy | MRD- CRS OR OS  NT CR | Jia Zhang | 2024 | 0 | 1 | 1 | 0 | 1 | 1 | 1 | 0 | 1 | 1 | 1 | 8 |
| MM | BCMA nolymphodeltion | MRD- CRS OR OS  NT CR | Jia Zhang | 2024 | 0 | 1 | 1 | 0 | 1 | 1 | 1 | 0 | 1 | 1 | 1 | 8 |
| MM | BCMA 41BB Lentiviral/4–1BB/ human | MRD- CRS OR OS  NT CR | Jia Zhang | 2024 | 0 | 1 | 1 | 0 | 1 | 1 | 1 | 0 | 1 | 1 | 1 | 8 |
| MM | BCMA 41BB Lentiviral/4–1BB/ murine | MRD- CRS OR OS  NT CR | Jia Zhang | 2024 | 0 | 1 | 1 | 0 | 1 | 1 | 1 | 0 | 1 | 1 | 1 | 8 |
| MM | CAR-T | ORR | Rita Pereira | 2024 | 0 | 1 | 1 | 0 | 1 | 1 | 1 | 0 | 1 | 1 | 1 | 8 |
| MM | CAR-T | MRD- | Rita Pereira | 2024 | 0 | 1 | 1 | 0 | 1 | 1 | 1 | 0 | 1 | 1 | 1 | 8 |
| MM | CAR-T | mDOR | Rita Pereira | 2024 | 0 | 1 | 1 | 0 | 1 | 1 | 1 | 0 | 1 | 1 | 1 | 8 |
| MM | CAR-T | ≥3 CRS | Rita Pereira | 2024 | 0 | 1 | 1 | 0 | 1 | 1 | 1 | 0 | 1 | 1 | 1 | 8 |
| MM | CAR-T | ≥3 Neurotoxicity | Rita Pereira | 2024 | 0 | 1 | 1 | 0 | 1 | 1 | 1 | 0 | 1 | 1 | 1 | 8 |
| MM | CAR-T | Any grade infection | Rita Pereira | 2024 | 0 | 1 | 1 | 0 | 1 | 1 | 1 | 0 | 1 | 1 | 1 | 8 |
| MM | CAR-T | ≥3 infection | Rita Pereira | 2024 | 0 | 1 | 1 | 0 | 1 | 1 | 1 | 0 | 1 | 1 | 1 | 8 |
| MM | Anti-MM ≥5 | ORR | Rita Pereira | 2024 | 0 | 1 | 1 | 0 | 1 | 1 | 1 | 0 | 1 | 1 | 1 | 8 |
| MM | Anti-MM＜5 | ORR | Rita Pereira | 2024 | 0 | 1 | 1 | 0 | 1 | 1 | 1 | 0 | 1 | 1 | 1 | 8 |
| MM | No BCMA prior | ORR | Rita Pereira | 2024 | 0 | 1 | 1 | 0 | 1 | 1 | 1 | 0 | 1 | 1 | 1 | 8 |
| MM | BCMA prior | ORR | Rita Pereira | 2024 | 0 | 1 | 1 | 0 | 1 | 1 | 1 | 0 | 1 | 1 | 1 | 8 |
| MM | High risk cytogenetics≥39% | ORR | Rita Pereira | 2024 | 0 | 1 | 1 | 0 | 1 | 1 | 1 | 0 | 1 | 1 | 1 | 8 |
| MM | High risk cytogenetics＜39% | ORR | Rita Pereira | 2024 | 0 | 1 | 1 | 0 | 1 | 1 | 1 | 0 | 1 | 1 | 1 | 8 |
| MM | Extramedullary disease＜28% | ORR | Rita Pereira | 2024 | 0 | 1 | 1 | 0 | 1 | 1 | 1 | 0 | 1 | 1 | 1 | 8 |
| MM | Extramedullary disease≥28% | ORR | Rita Pereira | 2024 | 0 | 1 | 1 | 0 | 1 | 1 | 1 | 0 | 1 | 1 | 1 | 8 |
| RRMM | BMCA 41BB PiggyBac retrovirus murine | ORR CRR MRD- CRS NT PFS OS DOR | Dingyuan Hu | 2023 | 1 | 1 | 1 | 0 | 1 | 1 | 1 | 0 | 1 | 1 | 1 | 9 |
| RRMM | ≥55 | ORR | Dingyuan Hu | 2023 | 1 | 1 | 1 | 0 | 1 | 1 | 1 | 0 | 1 | 1 | 1 | 9 |
| RRMM | ＜55 | ORR | Dingyuan Hu | 2023 | 1 | 1 | 1 | 0 | 1 | 1 | 1 | 0 | 1 | 1 | 1 | 9 |
| RRMM | ＞2e8 cells | ORR | Dingyuan Hu | 2023 | 1 | 1 | 1 | 0 | 1 | 1 | 1 | 0 | 1 | 1 | 1 | 9 |
| RRMM | ＜2e8 cells | ORR | Dingyuan Hu | 2023 | 1 | 1 | 1 | 0 | 1 | 1 | 1 | 0 | 1 | 1 | 1 | 9 |
| RRMM | Human Scfv | ORR | Dingyuan Hu | 2023 | 1 | 1 | 1 | 0 | 1 | 1 | 1 | 0 | 1 | 1 | 1 | 9 |
| RRMM | murine Scfv | ORR | Dingyuan Hu | 2023 | 1 | 1 | 1 | 0 | 1 | 1 | 1 | 0 | 1 | 1 | 1 | 9 |
| RRMM | LIama Scfv | ORR | Dingyuan Hu | 2023 | 1 | 1 | 1 | 0 | 1 | 1 | 1 | 0 | 1 | 1 | 1 | 9 |
| RRMM | 41BB | ORR | Dingyuan Hu | 2023 | 1 | 1 | 1 | 0 | 1 | 1 | 1 | 0 | 1 | 1 | 1 | 9 |
| RRMM | Others | ORR | Dingyuan Hu | 2023 | 1 | 1 | 1 | 0 | 1 | 1 | 1 | 0 | 1 | 1 | 1 | 9 |
| RRMM | Lenti | ORR | Dingyuan Hu | 2023 | 1 | 1 | 1 | 0 | 1 | 1 | 1 | 0 | 1 | 1 | 1 | 9 |
| RRMM | Retro | ORR | Dingyuan Hu | 2023 | 1 | 1 | 1 | 0 | 1 | 1 | 1 | 0 | 1 | 1 | 1 | 9 |
| RRMM | Media time≥4 years | ORR | Dingyuan Hu | 2023 | 1 | 1 | 1 | 0 | 1 | 1 | 1 | 0 | 1 | 1 | 1 | 9 |
| RRMM | Media time＜4 years | ORR | Dingyuan Hu | 2023 | 1 | 1 | 1 | 0 | 1 | 1 | 1 | 0 | 1 | 1 | 1 | 9 |
| RRMM | ≥8 lines | ORR | Dingyuan Hu | 2023 | 1 | 1 | 1 | 0 | 1 | 1 | 1 | 0 | 1 | 1 | 1 | 9 |
| RRMM | ＜8 lines | ORR | Dingyuan Hu | 2023 | 1 | 1 | 1 | 0 | 1 | 1 | 1 | 0 | 1 | 1 | 1 | 9 |
| RRMM | ≥75% ASCT | ORR | Dingyuan Hu | 2023 | 1 | 1 | 1 | 0 | 1 | 1 | 1 | 0 | 1 | 1 | 1 | 9 |
| RRMM | ＜75% ASCT | ORR | Dingyuan Hu | 2023 | 1 | 1 | 1 | 0 | 1 | 1 | 1 | 0 | 1 | 1 | 1 | 9 |
| RRMM | ≥48% High risk | ORR | Dingyuan Hu | 2023 | 1 | 1 | 1 | 0 | 1 | 1 | 1 | 0 | 1 | 1 | 1 | 9 |
| RRMM | ＜48% High risk | ORR | Dingyuan Hu | 2023 | 1 | 1 | 1 | 0 | 1 | 1 | 1 | 0 | 1 | 1 | 1 | 9 |
| RRMM | ≥29% extradisease | ORR | Dingyuan Hu | 2023 | 1 | 1 | 1 | 0 | 1 | 1 | 1 | 0 | 1 | 1 | 1 | 9 |
| RRMM | ＜29% extradisease | ORR | Dingyuan Hu | 2023 | 1 | 1 | 1 | 0 | 1 | 1 | 1 | 0 | 1 | 1 | 1 | 9 |
| RRMM | ≥3% ECOG | ORR | Dingyuan Hu | 2023 | 1 | 1 | 1 | 0 | 1 | 1 | 1 | 0 | 1 | 1 | 1 | 9 |
| RRMM | ＜3% ECOG | ORR | Dingyuan Hu | 2023 | 1 | 1 | 1 | 0 | 1 | 1 | 1 | 0 | 1 | 1 | 1 | 9 |
| RRMM | ≥28% ISS≥3 | ORR | Dingyuan Hu | 2023 | 1 | 1 | 1 | 0 | 1 | 1 | 1 | 0 | 1 | 1 | 1 | 9 |
| RRMM | ＜28% ISS≥3 | ORR | Dingyuan Hu | 2023 | 1 | 1 | 1 | 0 | 1 | 1 | 1 | 0 | 1 | 1 | 1 | 9 |
| RRMM | ≥39% mAb exposed | ORR | Dingyuan Hu | 2023 | 1 | 1 | 1 | 0 | 1 | 1 | 1 | 0 | 1 | 1 | 1 | 9 |
| RRMM | ＜39% mAb exposed | ORR | Dingyuan Hu | 2023 | 1 | 1 | 1 | 0 | 1 | 1 | 1 | 0 | 1 | 1 | 1 | 9 |
| RRMM | ≥55 | CRS | Dingyuan Hu | 2023 | 1 | 1 | 1 | 0 | 1 | 1 | 1 | 0 | 1 | 1 | 1 | 9 |
| RRMM | ＜55 | CRS | Dingyuan Hu | 2023 | 1 | 1 | 1 | 0 | 1 | 1 | 1 | 0 | 1 | 1 | 1 | 9 |
| RRMM | Human Scfv | CRS | Dingyuan Hu | 2023 | 1 | 1 | 1 | 0 | 1 | 1 | 1 | 0 | 1 | 1 | 1 | 9 |
| RRMM | murine Scfv | CRS | Dingyuan Hu | 2023 | 1 | 1 | 1 | 0 | 1 | 1 | 1 | 0 | 1 | 1 | 1 | 9 |
| RRMM | LIama Scfv | CRS | Dingyuan Hu | 2023 | 1 | 1 | 1 | 0 | 1 | 1 | 1 | 0 | 1 | 1 | 1 | 9 |
| RRMM | 41BB | CRS | Dingyuan Hu | 2023 | 1 | 1 | 1 | 0 | 1 | 1 | 1 | 0 | 1 | 1 | 1 | 9 |
| RRMM | Others | CRS | Dingyuan Hu | 2023 | 1 | 1 | 1 | 0 | 1 | 1 | 1 | 0 | 1 | 1 | 1 | 9 |
| RRMM | Lenti | CRS | Dingyuan Hu | 2023 | 1 | 1 | 1 | 0 | 1 | 1 | 1 | 0 | 1 | 1 | 1 | 9 |
| RRMM | Retro | CRS | Dingyuan Hu | 2023 | 1 | 1 | 1 | 0 | 1 | 1 | 1 | 0 | 1 | 1 | 1 | 9 |
| RRMM | Media time≥4 years | CRS | Dingyuan Hu | 2023 | 1 | 1 | 1 | 0 | 1 | 1 | 1 | 0 | 1 | 1 | 1 | 9 |
| RRMM | Media time＜4 years | CRS | Dingyuan Hu | 2023 | 1 | 1 | 1 | 0 | 1 | 1 | 1 | 0 | 1 | 1 | 1 | 9 |
| RRMM | ≥8 lines | CRS | Dingyuan Hu | 2023 | 1 | 1 | 1 | 0 | 1 | 1 | 1 | 0 | 1 | 1 | 1 | 9 |
| RRMM | ＜8 lines | CRS | Dingyuan Hu | 2023 | 1 | 1 | 1 | 0 | 1 | 1 | 1 | 0 | 1 | 1 | 1 | 9 |
| RRMM | ≥75% ASCT | CRS | Dingyuan Hu | 2023 | 1 | 1 | 1 | 0 | 1 | 1 | 1 | 0 | 1 | 1 | 1 | 9 |
| RRMM | ＜75% ASCT | CRS | Dingyuan Hu | 2023 | 1 | 1 | 1 | 0 | 1 | 1 | 1 | 0 | 1 | 1 | 1 | 9 |
| RRMM | ≥48% High risk | CRS | Dingyuan Hu | 2023 | 1 | 1 | 1 | 0 | 1 | 1 | 1 | 0 | 1 | 1 | 1 | 9 |
| RRMM | ＜48% High risk | CRS | Dingyuan Hu | 2023 | 1 | 1 | 1 | 0 | 1 | 1 | 1 | 0 | 1 | 1 | 1 | 9 |
| RRMM | ≥29% extradisease | CRS | Dingyuan Hu | 2023 | 1 | 1 | 1 | 0 | 1 | 1 | 1 | 0 | 1 | 1 | 1 | 9 |
| RRMM | ＜29% extradisease | CRS | Dingyuan Hu | 2023 | 1 | 1 | 1 | 0 | 1 | 1 | 1 | 0 | 1 | 1 | 1 | 9 |
| RRMM | ≥3% ECOG | CRS | Dingyuan Hu | 2023 | 1 | 1 | 1 | 0 | 1 | 1 | 1 | 0 | 1 | 1 | 1 | 9 |
| RRMM | ＜3% ECOG | CRS | Dingyuan Hu | 2023 | 1 | 1 | 1 | 0 | 1 | 1 | 1 | 0 | 1 | 1 | 1 | 9 |
| RRMM | ≥28% ISS≥3 | CRS | Dingyuan Hu | 2023 | 1 | 1 | 1 | 0 | 1 | 1 | 1 | 0 | 1 | 1 | 1 | 9 |
| RRMM | ＜28% ISS≥3 | ORR | Dingyuan Hu | 2023 | 1 | 1 | 1 | 0 | 1 | 1 | 1 | 0 | 1 | 1 | 1 | 9 |
| RRMM | ≥39% mAb exposed | ORR | Dingyuan Hu | 2023 | 1 | 1 | 1 | 0 | 1 | 1 | 1 | 0 | 1 | 1 | 1 | 9 |
| RRMM | ＜39% mAb exposed | ORR | Dingyuan Hu | 2023 | 1 | 1 | 1 | 0 | 1 | 1 | 1 | 0 | 1 | 1 | 1 | 9 |
| MM | BCMA CAR-T | CR | Nico Gagelmann | 2019 | 0 | 1 | 1 | 1 | 1 | 1 | 1 | 0 | 1 | 1 | 1 | 9 |
| MM | BCMA CAR-T | MRD | Nico Gagelmann | 2019 | 0 | 1 | 1 | 1 | 1 | 1 | 1 | 0 | 1 | 1 | 1 | 9 |
| MM | BCMA CAR-T | relapsed after car-t | Nico Gagelmann | 2019 | 0 | 1 | 1 | 1 | 1 | 1 | 1 | 0 | 1 | 1 | 1 | 9 |
| MM | BCMA CAR-T | relapsed after car-t | Nico Gagelmann | 2019 | 0 | 1 | 1 | 1 | 1 | 1 | 1 | 0 | 1 | 1 | 1 | 9 |
| MM | BCMA CAR-T | relapsed | Nico Gagelmann | 2019 | 0 | 1 | 1 | 1 | 1 | 1 | 1 | 0 | 1 | 1 | 1 | 9 |
| MM | BCMA CAR-T | CRS | Nico Gagelmann | 2019 | 0 | 1 | 1 | 1 | 1 | 1 | 1 | 0 | 1 | 1 | 1 | 9 |
| MM | BCMA CAR-T | Neurotoxicity | Nico Gagelmann | 2019 | 0 | 1 | 1 | 1 | 1 | 1 | 1 | 0 | 1 | 1 | 1 | 9 |
| RRMM | CAR-T | ORR | Han Xu | 2024 | 1 | 1 | 1 | 0 | 1 | 1 | 1 | 0 | 1 | 1 | 1 | 9 |
| RRMM | CAR-T | CR+sCR | Han Xu | 2024 | 1 | 1 | 1 | 0 | 1 | 1 | 1 | 0 | 1 | 1 | 1 | 9 |
| RRMM | CAR-T | MRD- | Han Xu | 2024 | 1 | 1 | 1 | 0 | 1 | 1 | 1 | 0 | 1 | 1 | 1 | 9 |
| RRMM | CAR-T | CRS | Han Xu | 2024 | 1 | 1 | 1 | 0 | 1 | 1 | 1 | 0 | 1 | 1 | 1 | 9 |
| RRMM | CAR-T | CRS≥3 | Han Xu | 2024 | 1 | 1 | 1 | 0 | 1 | 1 | 1 | 0 | 1 | 1 | 1 | 9 |
| RRMM | CAR-T | Neurotoxicity | Han Xu | 2024 | 1 | 1 | 1 | 0 | 1 | 1 | 1 | 0 | 1 | 1 | 1 | 9 |
| RRMM | CAR-T | 1 year recurrence | Han Xu | 2024 | 1 | 1 | 1 | 0 | 1 | 1 | 1 | 0 | 1 | 1 | 1 | 9 |
| RRMM | CAR-T | 2year recurrence | Han Xu | 2024 | 1 | 1 | 1 | 0 | 1 | 1 | 1 | 0 | 1 | 1 | 1 | 9 |
| RRMM | murine Scfv | ORR | Lina Zhang | 2021 | 1 | 1 | 1 | 0 | 1 | 1 | 1 | 0 | 1 | 1 | 1 | 9 |
| RRMM | Human Scfv | ORR | Lina Zhang | 2021 | 1 | 1 | 1 | 0 | 1 | 1 | 1 | 0 | 1 | 1 | 1 | 9 |
| RRMM | Dual-epitope Scfv | ORR | Lina Zhang | 2021 | 1 | 1 | 1 | 0 | 1 | 1 | 1 | 0 | 1 | 1 | 1 | 9 |
| RRMM | murine Scfv | CR | Lina Zhang | 2021 | 1 | 1 | 1 | 0 | 1 | 1 | 1 | 0 | 1 | 1 | 1 | 9 |
| RRMM | Human Scfv | CR | Lina Zhang | 2021 | 1 | 1 | 1 | 0 | 1 | 1 | 1 | 0 | 1 | 1 | 1 | 9 |
| RRMM | Dual-epitope Scfv | CR | Lina Zhang | 2021 | 1 | 1 | 1 | 0 | 1 | 1 | 1 | 0 | 1 | 1 | 1 | 9 |
| RRMM | Human -low doses | ORR | Lina Zhang | 2021 | 1 | 1 | 1 | 0 | 1 | 1 | 1 | 0 | 1 | 1 | 1 | 9 |
| RRMM | Human -high doses | ORR | Lina Zhang | 2021 | 1 | 1 | 1 | 0 | 1 | 1 | 1 | 0 | 1 | 1 | 1 | 9 |
| RRMM | older | ORR | Lina Zhang | 2021 | 1 | 1 | 1 | 0 | 1 | 1 | 1 | 0 | 1 | 1 | 1 | 9 |
| RRMM | Young | ORR | Lina Zhang | 2021 | 1 | 1 | 1 | 0 | 1 | 1 | 1 | 0 | 1 | 1 | 1 | 9 |
| RRMM | Relapsed | ORR | Lina Zhang | 2021 | 1 | 1 | 1 | 0 | 1 | 1 | 1 | 0 | 1 | 1 | 1 | 9 |
| RRMM | Multiple Relapsed | ORR | Lina Zhang | 2021 | 1 | 1 | 1 | 0 | 1 | 1 | 1 | 0 | 1 | 1 | 1 | 9 |
| RRMM | murine Scfv | ORR | Lina Zhang | 2021 | 1 | 1 | 1 | 0 | 1 | 1 | 1 | 0 | 1 | 1 | 1 | 9 |
| RRMM | Human Scfv | ORR | Lina Zhang | 2021 | 1 | 1 | 1 | 0 | 1 | 1 | 1 | 0 | 1 | 1 | 1 | 9 |
| RRMM | Dual-epitope Scfv | ORR | Lina Zhang | 2021 | 1 | 1 | 1 | 0 | 1 | 1 | 1 | 0 | 1 | 1 | 1 | 9 |
| RRMM | older | ORR | Lina Zhang | 2021 | 1 | 1 | 1 | 0 | 1 | 1 | 1 | 0 | 1 | 1 | 1 | 9 |
| RRMM | Young | ORR | Lina Zhang | 2021 | 1 | 1 | 1 | 0 | 1 | 1 | 1 | 0 | 1 | 1 | 1 | 9 |
| RRMM | Relapsed | ORR | Lina Zhang | 2021 | 1 | 1 | 1 | 0 | 1 | 1 | 1 | 0 | 1 | 1 | 1 | 9 |
| RRMM | Multiple Relapsed | ORR | Lina Zhang | 2021 | 1 | 1 | 1 | 0 | 1 | 1 | 1 | 0 | 1 | 1 | 1 | 9 |
| RRMM | murine Scfv | ORR | Lina Zhang | 2021 | 1 | 1 | 1 | 0 | 1 | 1 | 1 | 0 | 1 | 1 | 1 | 9 |
| RRMM | Human Scfv | ORR | Lina Zhang | 2021 | 1 | 1 | 1 | 0 | 1 | 1 | 1 | 0 | 1 | 1 | 1 | 9 |
| RRMM | Dual-epitope Scfv | ORR | Lina Zhang | 2021 | 1 | 1 | 1 | 0 | 1 | 1 | 1 | 0 | 1 | 1 | 1 | 9 |
| MM | BCMA 65years up | ORR | Othman Salim Akhtar | 2024 | 0 | 1 | 1 | 0 | 1 | 1 | 1 | 0 | 1 | 0 | 1 | 8 |
| MM | BCMA 65years down | ORR | Othman Salim Akhtar | 2024 | 0 | 1 | 1 | 0 | 1 | 1 | 1 | 0 | 1 | 0 | 1 | 8 |
| MM | BCMA 65years up | 3 CRS | Othman Salim Akhtar | 2024 | 0 | 1 | 1 | 0 | 1 | 1 | 1 | 0 | 1 | 0 | 1 | 8 |
| MM | BCMA 65years down | 3 CRS | Othman Salim Akhtar | 2024 | 0 | 1 | 1 | 0 | 1 | 1 | 1 | 0 | 1 | 0 | 1 | 8 |
| MM | BCMA 65years up | ICANS | Othman Salim Akhtar | 2024 | 0 | 1 | 1 | 0 | 1 | 1 | 1 | 0 | 1 | 0 | 1 | 8 |
| MM | BCMA 65years down | ICANS | Othman Salim Akhtar | 2024 | 0 | 1 | 1 | 0 | 1 | 1 | 1 | 0 | 1 | 0 | 1 | 8 |
| AMSTAR, a measurement tool to assess systematic reviews; RRMM relapsed or refractory multiple myeloma; R/R ALL: relapsed or refractory acute lymphoblastic leukemia; CLL chronic lymphocytic leukemia; R/R DLBCL relapsed or refractory diffuse large B-cell lymphoma; LBCL relapsed/refractory large B-cell lymphoma; B-NHL: B-cell non-Hodgkin lymphoma; CNSL central nervous system lymphoma; CAR-T chimeric antigen receptor T; GCB: Germinal Center B-cell-like; n-GCB: Non-Germinal Center B-cell-like; HGBL: High-grade B-cell lymphoma; MCL: Mantle Cell Lymphoma; PCL: Primary Cutaneous Lymphoma; FL: Follicular Lymphoma; Fup: Follow-up; RD response duration; OVS overall survival; BOR: Best Overall Response；ORR: overall response rate; ICANS: immune cell-effector-associated neurotoxicity syndrome; CRS: cytokine release syndrome; CRR: complete response rate; MRD: minimal residual disease negativity; OS: overall survival; GVHD:The graft-versus-host disease;NT: neurotoxicity;ASCT: autologous stem-cell transplantation; Lenti, Lentiviral vector; BBz, 41BB receptor with Zeta chain; BENDAM: Bendamustine; Flu/cy: Fludarabine and Cyclophosphamide; Alem-tuzumab: Alemtuzumab; IFOS: Ifosfamide. | | | | | | | | | | | | | | | | |
