# Supplementary material for: Efficacy and safety of chimeric antigen receptor T-cell in the treatment of hematologic malignancy: an umbrella review of systematic review and meta-analysis
Source: Front Immunol. 2025 Nov 19;16:1608768. doi: 10.3389/fimmu.2025.1608768 (PMC12672455; doi:10.3389/fimmu.2025.1608768)
Supplement: Supplementary file 3 [file Table3.docx]

| Supplementary Table S2. Assessments of GRADE scores. | | | | | | | | | | | | | |
| --- | --- | --- | --- | --- | --- | --- | --- | --- | --- | --- | --- | --- | --- |
| Cancer | CAR-T | Outcomes | Study | No. of  studies | Risk of  bias | Inconsistency | Indirectness | Imprecision | Publication bias | Plausible confounding | Magnitude of  effect | Dose-response gradient | Quality |
| CNSL | Age ＞60 vs ＜60 | DOR | Zhou2024 | 25 | no serious  risk | no serious inconsistency | no serious  indirectness | serious  imprecision | undetected | would not  reduce effect | no | no | Moderate |
| CNSL | 41BB+CD28 vs CD28 | DOR | Zhou2024 | 30 | no serious  risk | no serious inconsistency | no serious  indirectness | serious  imprecision | undetected | would not  reduce effect | yes | no | High |
| CNSL | 41BB+CD28 vs 41BB | DOR | Zhou2024 | 30 | no serious  risk | no serious inconsistency | no serious  indirectness | serious  imprecision | undetected | would not  reduce effect | yes | no | High |
| CNSL | Line therapy ＞5 vs ＜5 | DOR | Zhou2024 | 29 | no serious  risk | no serious inconsistency | no serious  indirectness | serious  imprecision | undetected | would not  reduce effect | no | no | Moderate |
| CNSL | Prior ASCT vs no | DOR | Zhou2024 | 27 | no serious  risk | no serious inconsistency | no serious  indirectness | serious  imprecision | undetected | would not  reduce effect | no | no | Moderate |
| CNSL | CAR-T：SD or PD vs PR | DOR | Zhou2024 | 28 | no serious  risk | serious  inconsistency | no serious  indirectness | serious  imprecision | undetected | would not  reduce effect | yes | no | Moderate |
| CNSL | Isolated vs systemic CNS | DOR | Zhou2024 | 18 | no serious  risk | serious  inconsistency | no serious  indirectness | serious  imprecision | undetected | would not  reduce effect | yes | no | Moderate |
| CNSL | CAR-Tvs ASCT+CAR-T | DOR | Zhou2024 | 31 | no serious  risk | serious  inconsistency | no serious  indirectness | serious  imprecision | undetected | would not  reduce effect | yes | no | Moderate |
| CNSL | CAR-TvsCAR-T+maintenance | DOR | Zhou2024 | 29 | no serious  risk | serious  inconsistency | no serious  indirectness | serious  imprecision | undetected | would not  reduce effect | yes | no | Moderate |
| CNSL | CAR-T：noPR vs PR | DOR | Zhou2024 | 44 | no serious  risk | serious  inconsistency | no serious  indirectness | serious  imprecision | undetected | would not  reduce effect | yes | no | Moderate |
| CNSL Multi | CAR-Tvs ASCT+CAR-T | DOR | Zhou2024 | 17 | no serious  risk | serious  inconsistency | no serious  indirectness | serious  imprecision | undetected | would not  reduce effect | yes | no | Moderate |
| CNSL Multi | CAR-TvsCAR-T+maintenance | DOR | Zhou2024 | 4 | no serious  risk | serious  inconsistency | no serious  indirectness | serious  imprecision | undetected | would not  reduce effect | yes | no | Moderate |
| R/R DLBCL | CD28 CD19,CD20CAR-T | CR | Cao 2020 | 7 | no serious  risk | no serious inconsistency | no serious  indirectness | serious  imprecision | undetected | would not  reduce effect | no | no | Moderate |
| R/R DLBCL | 41BB CD19,CD20CAR-T | CR | Cao 2020 | 6 | no serious  risk | no serious inconsistency | no serious  indirectness | serious  imprecision | undetected | would not  reduce effect | no | no | Moderate |
| R/R DLBCL | CD28 VS 41BB CD19,CD20 | CR | Cao 2020 | 13 | no serious  risk | no serious inconsistency | no serious  indirectness | serious  imprecision | undetected | would not  reduce effect | no | no | Moderate |
| R/R DLBCL | CD19 CAR-T | CR | Cao 2020 | 9 | no serious  risk | no serious inconsistency | no serious  indirectness | serious  imprecision | undetected | would not  reduce effect | no | no | Moderate |
| R/R DLBCL | CD20 CAR-T | CR | Cao 2020 | 3 | no serious  risk | no serious inconsistency | no serious  indirectness | serious  imprecision | strongly suspected | would not  reduce effect | no | no | low |
| R/R DLBCL | CD20 CAR-T | CR | Cao 2020 | 12 | no serious  risk | no serious inconsistency | no serious  indirectness | serious  imprecision | undetected | would not  reduce effect | no | no | Moderate |
| DLBCL | Axi-cel vs Tisa-ce; | OR | Gagelmann2024 | 7 | no serious  risk | serious  inconsistency | no serious  indirectness | imprecision | undetected | would not  reduce effect | no | no | High |
| DLBCL | Axi-cel vs Tisa-ce; | CR | Gagelmann2024 | 7 | no serious  risk | serious  inconsistency | no serious  indirectness | imprecision | undetected | would not  reduce effect | no | no | High |
| DLBCL | Axi-cel vs Tisa-ce; | PFS | Gagelmann2024 | 7 | no serious  risk | serious  inconsistency | no serious  indirectness | imprecision | undetected | would not  reduce effect | no | no | High |
| DLBCL | Axi-cel vs Tisa-ce; | OS | Gagelmann2024 | 7 | no serious  risk | serious  inconsistency | no serious  indirectness | serious  imprecision | undetected | would not  reduce effect | no | no | Moderate |
| DLBCL | Axi-cel vs Tisa-ce; | Any CRS | Gagelmann2024 | 7 | no serious  risk | serious  inconsistency | no serious  indirectness | imprecision | undetected | would not  reduce effect | yes | no | High |
| DLBCL | Axi-cel vs Tisa-ce; | Severe CRS | Gagelmann2024 | 7 | no serious  risk | no serious inconsistency | no serious  indirectness | serious  imprecision | strongly suspected | would not  reduce effect | no | no | Low |
| DLBCL | Axi-cel vs Tisa-ce; | Any ICANS | Gagelmann2024 | 7 | no serious  risk | serious  inconsistency | no serious  indirectness | imprecision | undetected | would not  reduce effect | yes | no | High |
| DLBCL | Axi-cel vs Tisa-ce; | Severe ICANS | Gagelmann2024 | 7 | no serious  risk | serious  inconsistency | no serious  indirectness | imprecision | undetected | would not  reduce effect | yes | no | High |
| DLBCL | Axi-cel vs Tisa-ce; | Severe neutropenia | Gagelmann2024 | 7 | no serious  risk | serious  inconsistency | no serious  indirectness | imprecision | undetected | would not  reduce effect | yes | no | High |
| ALL | Cy/flu vs other | MRD- | Nagle 2019 | 6 | no serious  risk | no serious inconsistency | no serious  indirectness | serious  imprecision | NA | would not  reduce effect | no | no | Moderate |
| ALL | retro vs lentivirus | MRD- | Nagle 2019 | 6 | no serious  risk | no serious inconsistency | no serious  indirectness | serious  imprecision | NA | would not  reduce effect | no | no | Moderate |
| ALL | Cy/flu vs other | sCRS | Nagle 2019 | 5 | no serious  risk | no serious inconsistency | no serious  indirectness | serious  imprecision | NA | would not  reduce effect | no | no | Moderate |
| ALL | retro vs lentivirus | sCRS | Nagle 2019 | 6 | no serious  risk | no serious inconsistency | no serious  indirectness | serious  imprecision | NA | would not  reduce effect | no | no | Moderate |
| ALL | CAR-T | sCRS | Nagle 2019 | 5 | no serious  risk | no serious inconsistency | no serious  indirectness | serious  imprecision | undetected | would not  reduce effect | no | no | Moderate |
| ALL | CD19 CAR-T | Neurotoxicity | Nagle 2019 | 3 | no serious  risk | no serious inconsistency | no serious  indirectness | serious  imprecision | undetected | would not  reduce effect | no | no | Moderate |
| B- ALL | CD19 NO HSCTvs + HSCT | Relapse rate | Willyanto2024 | 2 | no serious  risk | no serious inconsistency | no serious  indirectness | serious  imprecision | NA | would not  reduce effect | yes | no | High |
| B- ALL | CD22 NO HSCTvs + HSCT | Relapse rate | Willyanto2024 | 1 | no serious  risk | no serious inconsistency | no serious  indirectness | serious  imprecision | NA | would not  reduce effect | yes | no | High |
| B- ALL | HSCT vs CAR-T+ HSCT | Relapse rate | Willyanto2024 | 1 | no serious  risk | no serious inconsistency | no serious  indirectness | serious  imprecision | NA | would not  reduce effect | no | no | Moderate |
| R/R ALL | CD19 VS DLI | CRR | Saiz 2023 | 2 | no serious  risk | no serious inconsistency | no serious  indirectness | serious  imprecision | NA | would not  reduce effect | yes | no | High |
| R/R ALL | CD19 VS SoC | AEs | Saiz 2023 | 2 | no serious  risk | no serious inconsistency | no serious  indirectness | serious  imprecision | NA | would not  reduce effect | no | no | Moderate |
| R/R ALL | CD19 VS SoC | PR | Saiz 2023 | 2 | no serious  risk | no serious inconsistency | no serious  indirectness | serious  imprecision | NA | would not  reduce effect | no | no | Moderate |
| AEs: Adverse Events; ALL: Acute Lymphoblastic Leukemia; ASCT: Autologous Stem Cell Transplant; Axi-cel: Axicabtagene Ciloleucel; B-ALL: B-cell Acute Lymphoblastic Leukemia; CAR-T: Chimeric Antigen Receptor T-cell Therapy; CNSL: Central Nervous System Lymphoma; CI: Confidence Interval; CR: Complete Response; CRR: Complete Response Rate; CRS: Cytokine Release Syndrome; Cy/flu: Cyclophosphamide/Fludarabine; DLI: Donor Lymphocyte Infusion; DLBCL: Diffuse Large B-Cell Lymphoma; DOR: Duration of Response; Δ: Final Value - Baseline Value; GRADE: Grading of Recommendations Assessment, Development, and Evaluation; HR: Hazard Ratio; HSCT: Haematopoietic Stem Cell Transplantation; ICANS: Immune Effector Cell-Associated Neurotoxicity Syndrome; Multi: Multiple; MRD-: Minimal Residual Disease Negative; NA: Not Available; Neurotoxicity: Neurological Adverse Effects; OR: Odds Ratio; OS: Overall Survival; P: Population-Based Case-Control and/or Cross-Sectional Studies; PD: Progressive Disease; PFS: Progression-Free Survival; PR: Partial Remission; R/R ALL: Relapsed/Refractory Acute Lymphoblastic Leukemia; R/R DLBCL: Relapsed/Refractory Diffuse Large B-Cell Lymphoma; sCRS: Severe Cytokine Release Syndrome; SD: Stable Disease; SoC: Standard of Care; T: Total Number of Studies; Tisa-cel: Tisagenlecleucel. | | | | | | | | | | | | | |
